# Putting the behavior into animal movement modeling: Improved activity budgets from use of ancillary tag information

**DOI:** 10.1002/ece3.2530

**Published:** 2016-10-20

**Authors:** Sophie Bestley, Ian Jonsen, Robert G. Harcourt, Mark A. Hindell, Nicholas J. Gales

**Affiliations:** ^1^ Australian Antarctic DivisionDepartment of EnvironmentKingstonTas.Australia; ^2^ Institute for Marine and Antarctic StudiesUniversity of TasmaniaHobartTas.Australia; ^3^ Antarctic Climate and Ecosystems Co‐operative Research CentreHobartTas.Australia; ^4^ Department of Biological SciencesMacquarie UniversitySydneyNSWAustralia

**Keywords:** ancillary information, animal movement, Antarctic seals, behavioral switching, foraging behavior, Integrated Marine Observing System, marine predators, satellite tracking, state‐space model

## Abstract

Animal movement research relies on biotelemetry, and telemetry‐based locations are increasingly augmented with ancillary information. This presents an underutilized opportunity to enhance movement process models. Given tags designed to record specific behaviors, efforts are increasing to update movement models beyond reliance solely upon horizontal movement information to improve inference of space use and activity budgets. We present two state‐space models adapted to incorporate ancillary data to inform three discrete movement states: directed, resident, and an activity state. These were developed for two case studies: (1) a “haulout” model for Weddell seals, and (2) an “activity” model for Antarctic fur seals which intersperse periods of diving activity and inactivity. The methodology is easily implementable with any ancillary data that can be expressed as a proportion (or binary) indicator. A comparison of the models augmented with ancillary information and unaugmented models confirmed that many behavioral states appeared mischaracterized in the latter. Important changes in subsequent activity budgets occurred. Haulout accounted for 0.17 of the overall Weddell seal time budget, with the estimated proportion of time spent in a resident state reduced from a posterior median of 0.69 (0.65–0.73; 95% HPDI) to 0.54 (0.50–0.58 HPDI). The drop was more dramatic in the Antarctic fur seal case, from 0.57 (0.52–0.63 HPDI) to 0.22 (0.20–0.25 HPDI), with 0.35 (0.31–0.39 HPDI) of time spent in the inactive (nondiving) state. These findings reinforce previously raised contentions about the drawbacks of behavioral states inferred solely from horizontal movements. Our findings have implications for assessing habitat requirements; estimating energetics and consumption; and management efforts such as mitigating fisheries interactions. Combining multiple sources of information within integrated frameworks should improve inference of relationships between movement decisions and fitness, the interplay between resource and habitat dependencies, and their changes at the population and landscape level.

## 1 Introduction

In both aquatic and terrestrial realms, the study of animal movement increasingly relies on the use of biotelemetry methods (Hussey et al., 2015; Kays, Crofoot, Jetz, & Wikelski, 2015). For many highly migratory and cryptic marine species, telemetry provides the only practical means to determine critical components of life‐history and ecological strategies, for example, spatial usage, migration pathways, site fidelity, foraging behavior, and resource dependence. Behavioral strategies used by individuals and their fitness consequences have potentially important ecological, evolutionary, and conservation implications (Bolnick et al., 2003; Morales et al., 2010; Sutherland, 1996). Yet there has been a struggle to obtain sufficiently large data sets, in terms of number of individuals tagged, to truly help address applied management questions at population and ecosystem levels, such as those of spatial conservation planning and ecosystem‐based fisheries management. However, with modern technological advances reducing tagging costs (Ropert‐Coudert & Wilson, 2005), large‐scale international collaborative projects (Biuw et al., 2007; Block et al., 2011) and state‐of‐the‐art analytical methods (Morales et al., 2010; Patterson, Thomas, Wilcox, Ovaskainen, & Matthiopoulos, 2008) are together beginning to realize these efforts.

State‐space models are process‐based models that are now widely used to draw population‐level inferences about hidden behaviors from noisy and complex individual‐based telemetry time‐series observations (Jonsen et al., 2013; Schick et al., 2008). In general, research questions are moving beyond simply where groups of animals go, to what they are doing there. A commonly used approach (Jonsen, Myers, & Flemming, 2003; Langrock et al., 2012; Morales, Haydon, Frair, Holsiner, & Fryxell, 2004) models the movement process using some type of biased and/or correlated random walk (CRW) model. This may incorporate switching between two behavioral states (nominally directed or resident behaviors) as described by a mixture of CRWs, each having an associated set of governing parameters. Increasingly, efforts are being directed toward more sophisticated process models addressing, for example, behavioral associations, environmental influences, and the role of memory (Fagan et al., 2013; Langrock et al., 2014; McClintock et al., 2012). While precise Global Positioning System (GPS) location data can yield highly detailed insight into animal movement, even with error‐free location data it can be important to consider additional information as different behaviors could have similar movement signatures at the scale of observation. Less precise location data, such as obtained via the Argos satellite system, often do not contain sufficient information alone to support inference of more than two behavioral states. However, many double tagging efforts (i.e.*,* using more than one type of telemetry device) and/or increasingly sophisticated telemetry devices mean that a great deal of useful ancillary information exists which can be used to support more complicated behavioral models (Dean et al., 2013; McClintock, Russell, Matthiopoulos, & King, 2013; Russell et al., 2015).

Achieving greater biological realism in animal movement models is essential for making correct inferences about space use and behavior, and developing activity or energy budgets. For example, movement models frequently applied to marine mammals (Jonsen, Flemming, & Myers, 2005; Morales et al., 2004) may oversimplify complex behaviors (Beatty, Jay, & Fischbach, 2016; Ramasco, Barraquand, Biuw, McConnell, & Nilssen, 2015). Time spent in activities other than transit or forage can be important, for example, resting, predator evasion, or social behavior, so an increased harnessing of activity information may yield greater biological realism in movement process models. Models incorporating ancillary activity data may also be of significant practical use. Researchers often undertake the laborious task of breaking tracks into discrete trips, removing apparent resting, or haulout periods prior to subsequent analysis (Breed, Jonsen, Myers, Bowen, & Leonard, 2009). Provision of a wider array of model structures will allow the biotelemetry community greater flexibility in appropriately addressing the ecology of their study species. The widespread uptake of such analytical approaches, however, can only be facilitated by making them readily available, understandable, and easy to implement.

Here, we present two state‐space model formulations that incorporate ancillary telemetry data to inform activity state. Our approach builds on Bayesian behavioral switching state‐space models widely used for error‐prone Argos locations (Jonsen et al., 2005) and GPS locations (Morales et al., 2004). Inference of a third behavioral state is supported by the ancillary information. The two model formulations are developed for particular case studies: (1) Weddell seals, which regularly haul out onto ice in Antarctic waters, and (2) Antarctic fur seals, which demonstrate a (potentially inconsistent) diel pattern in their diving activity and inactivity. These examples provide clear demonstrations of how harnessing of activity information can deliver more robust behavioral inference. We discuss the broader utility and implications with reference to the assessment of present and future habitat requirements; estimation of energetics and consumption; and management efforts such as mitigating fisheries interactions.

## 2 Methods

### 2.1 State‐space model formulation: the movement process

The Bayesian state‐space models described here comprise two components: (i) the process model which statistically describes how the behavioral and location states evolve over time in a first‐order Markov process framework, and (ii) the observation model which describes how the irregularly observed and error‐prone locations are generated, conditional on the state of the system at regular time intervals. The process and observational models have been fully described elsewhere (Jonsen et al., 2005) and are reformulated in Appendix S1. Here, we focus on how the details specifying the process model differ between the typical two‐ or three‐state model, and the three‐state formulations augmented with ancillary telemetry information. The SSMs are implemented using the freely available software JAGS (Plummer, 2013; http://mcmc-jags.sourceforge.net) and the R package *rjags* (Plummer, 2015). The code for each model formulation is provided in the Supporting Information online (Appendix S2) together with worked examples (Data S1).

Consider the case where an animal may be in one of two unobservable behavioral states at time *t*, labeled here as “directed” (*D*_*t*_, i.e.*,* more transitory) and “resident” (*R*_*t*_, i.e., more localized). The time dependence in the behavioral process is described by a Markov chain, specified by the probability of switching states from *D* at time *t*−1 to *R* at time *t*. The model is written in terms of a transition matrix determining the switching probabilities (φ):

$$\varphi =\left(\begin{array}{cc} \text{Pr}(D_{t}|D_{t-1}) & \text{Pr}(R_{t}|D_{t-1})\\ \text{Pr}(D_{t}|R_{t-1}) & \text{Pr}(R_{t}|R_{t-1}) \end{array}\right)$$

whose elements are constrained so that the rows sum to 1. The state variable **b**_*t*_ represents the state at any time: $b_{t}=\left[\text{Pr}\left(D_{t}\right)\;\text{Pr}\left(R_{t}\right)\right]$. The probability of each behavioral state is updated through time by $b_{t}=\varphi \cdot b_{t-1}$. Extending this formulation to three or more states is in principle straightforward. Let us generalize to label the states using *s*_*i*_, including *i* as the behavioral state index i∈[1,2,3] where 1 = “directed,” 2 = “resident,” and 3 = “activity” (*A*_*t*_) a third behavioral state. The switching probabilities are then determined by:

$$\varphi =\left(\begin{array}{ccc} \text{Pr}(S_{1,t}|S_{1,t-1}) & \text{Pr}(S_{2,t}|S_{1,t-1}) & \text{Pr}(S_{3,t}|S_{1,t-1})\\ \text{Pr}(S_{1,t}|S_{2,t-1}) & \text{Pr}(S_{2,t}|S_{2,t-1}) & \text{Pr}(S_{3,t}|S_{2,t-1})\\ \text{Pr}(S_{1,t}|S_{3,t-1}) & \text{Pr}(S_{2,t}|S_{3,t-1}) & \text{Pr}(S_{3,t}|S_{3,t-1}) \end{array}\right)$$

Now $b_{t}=\left[\text{Pr}\left(D_{t}\right)\;\text{Pr}\left(R_{t}\right)\;\text{Pr}\left(A_{t}\right)\right]=\left[\text{Pr}\left(S_{1,t}\right)\;\text{Pr}\left(S_{2,t}\right)\;\text{Pr}\left(S_{3,t}\right)\right]$. In the state‐space model employed here, each behavioral state is associated with a distinct CRW behavior (Jonsen et al., 2005); see Appendix S2 models (i) and (ii)). The CRWs, and the states they describe at time *t*, differ in their values of mean turning angle ($\vartheta_{i}$), which controls the rotational component, and movement persistence (γ_*i*_). The latter parameter describes the degree to which the random walk is (first order) autocorrelated in both direction and move speed, where γ = 0 yields a simple random walk and 0 < γ < 1 yields a correlation in both direction and speed. The process model has a hierarchical formulation insofar as the parameters are estimated across multiple individual animals; examining individual variation in behavior via the implementation of a fully hierarchical model (Jonsen, Myers, & James, 2006) is a feasible future extension. The probabilities of switching from one state to another ($\varphi_{i,t}$) are static, although they may also be formulated to vary with time in relation to other behavioral and/or environmental covariate influences (Bestley, Jonsen, Hindell, Guinet, & Charrassin, 2013).

The process model is specified without reference to the observed data. In practice, however, Argos location fixes are characterized by a heavy‐tailed error distribution (Costa et al., 2010; Vincent, McConnell, Ridoux, & Fedak, 2002). This is aggravated by the life‐history strategies of diving marine animals, which typically result in a high proportion of low‐quality (inaccurate) location classes. Obtaining good model fits can prove challenging, depending on the nature of the movements displayed by different species, such that it may be problematic to infer three (or more) behavioral states based solely on horizontal movement characteristics (Bestley, Jonsen, Hindell, Harcourt, & Gales, 2015). To support the inference of more complex behavior, from such noisy observations, we therefore turn to the rich auxiliary activity information that is often available in tagging studies.

### 2.2 Case study 1: a haulout model for Weddell seals

Weddell seals (*Leptonychotes weddellii*) are an ice‐obligate Antarctic species that inhabits sea‐ice year round (Heerah et al., 2013). As well as requiring fast ice as a substrate for breeding and molting, Weddell seals actively forage beneath the ice and can spend substantial periods hauled out upon it (Andrews‐Goff, Hindell, Field, Wheatley, & Charrassin, 2010). Their movement tracks are typically highly localized within coastal bays and along the Antarctic continental shelf, so the latter two activities are not easily inferred from error‐prone Argos locations.

To include haulout as an activity state in our behavioral process model, we incorporate ancillary information available from the Satellite Relayed Data Loggers (SRDLs) manufactured by Sea Mammal Research Unit (SMRU, University of St Andrews, Scotland, UK). This tag provides the start and end times of a sequence of known haulout periods (recognizing a haulout when the tag was dry for 10 min, and ending the haulout once wet for 40 s). This enables the proportion of time spent hauled out to be calculated for each regular time interval within the movement model. We note that, depending on the uplink frequency and the specific cycling and ranking of data types awaiting transmission, this may not comprise a complete record. For the demonstration here, we consider it sufficient; however, a more complete treatment could also model the missing haulouts. The tagging data used (*n *=* *7 adult females, *N *=* *9,238 Argos locations, *N *=* *793 haulout observations) form part of the Australian Integrated Marine Observing System Davis station 2011 deployments (previously published in Bestley et al. 2015), and are publicly available (http://www.imos.org.au).

The important aspect for defining the structure of this process model is that the activity state (haulout) precludes any other behavior (i.e., directed or resident). In this case, reference is first made to the ancillary data to define Pr(*A*_*t*_) and $a_{t}=\left[\text{Pr}\left(A_{t}\right)\;1-\text{Pr}\left(A_{t}\right)\right]$ the haulout status where $a_{t}\in \left[1,2\right]$ and 1 = “haulout,” 2 = “not hauled out.” The switch probabilities into *s*_*3*_ (i.e. $\varphi_{1,3}$ and $\varphi_{2,3}$) therefore need not be estimated within the model; in practice, this is achieved by employing Dirichlet priors for φ and setting these two components to zero (see Appendix S2 model (iii)). Hence: $b_{t}=\left[0\;0\;1\right]$ where $a_{t}=1$ and $b_{t}=\left[\text{Pr}\left(D_{t}\right)\;\text{Pr}\left(R_{t}\right)\;0\right]$ where $a_{t}=2$.

### 2.3 Case study 2: an activity model for Antarctic fur seals

Antarctic fur seals (*Arctocephalus gazella*; AFS) are one of the most abundant top predators in the Southern Ocean, feeding on krill and/or myctophid fish (Raymond et al., 2011; Staniland et al., 2010). While female AFS typically forage nocturnally, and are relatively inactive during the day, comparatively little is known about males and the available evidence suggests their dive behavior may exhibit greater variability (Staniland & Robinson, 2008).

We seek to capture the active versus inactive diving periods (putatively foraging and nonforaging) to more accurately determine the activity budget, that is, the time allocation per state. This can have implications for how we infer habitat use: for example, identifying key Southern Ocean forage grounds, and also for scaling up to construct estimates of prey consumption on these forage grounds. The ancillary data used for this can comprise any time‐series of diving behavior, such as is commonly available from time–depth recorders (TDRs). We again use SMRU SRDL data, this time the 6 h binned summaries of the proportion of time spent diving. These tagging data (*n *=* *5 adult males, *N *=* *4,597 Argos locations, *N *=* *1,800 dive summary observations) form part of the 2004 Heard Island Predators and Prey Ecosystem Study (Frydman & Gales, 2007) and are also publicly available (https://data.aad.gov.au).

Using the process model described above for Weddell seals would be sufficient if we were primarily interested in discriminating the inactive behavioral state, across a complete migration path. In principle, the activity state (inactive, meaning nondiving) does not preclude any other behavior (i.e., an animal may be nondiving while in a directed or resident state). It was, however, our objective to hone in specifically on the Southern Ocean foraging grounds and discriminate within the apparently resident phase when and where an animal is actively diving or inactive (nondiving). Construction of this process model is therefore subtly different. In practice, we estimate all switching probabilities, but switches into the directed state ($\varphi_{i,1}$) are estimated from the horizontal movements (see Appendix S2 model (iv)). For the remainder (i.e.*,* $\varphi_{i,2}$ and $\varphi_{i,3}$), the ancillary data are used to define the activity status Pr(*A*_*t*_) and $a_{t}=\left[\text{Pr}\left(A_{t}\right)\;1-\text{Pr}\left(A_{t}\right)\right]$ where $a_{t}\in \left[1,2\right]$ and 1 = “inactive,” 2 = “active diving.” Hence: $b_{t}=\left[0\;0\;1\right]$ where $a_{t}=1$ and $b_{t}=\left[0\;1\;0\right]$ where $a_{t}=2$.

For air‐breathing divers, operating within some physiological threshold, the proportion of time spent diving can never be 100% for a time step sufficiently long (Russell et al., 2014, 2015). However, the proportion of time spent nondiving (inactive) may be, within reasonable bounds. Our approach allocated the probability of being in an “inactive” state per time step as Pr(*A*_*t*_) = 1 only where no diving behavior was recorded at all. Any dive behavior served to rescale Pr(*A*_*t*_) < 0.5 with the highest proportional time spent diving serving to drive Pr(*A*_*t*_)~0 (see Data S1). Missing data were treated as uninformative (i.e. Pr(*A*_*t*_) = 0.5).

### 2.4 Model implementation

For both case studies, the CRW time step was set at 6 h to match the availability of the ancillary tag data. To fit the SSMs, two Markov chain Monte Carlo (MCMC) chains of 40,000 iterations were run with a burn‐in of 20 000. Each chain was thinned so that one in every 20 samples was retained for a final posterior sample size of 2000. The deviance ($\overset{¯}{D}$) and effective number of parameters (*pD*) were monitored along with the movement parameters, and here, we report mainly on $\vartheta_{i}$, $\gamma_{i}$, $\varphi_{i,j}$, and **b**_*t*_.

In general, weak priors were adopted throughout (Appendix S3, Table S3.1); however, in order to prevent state‐flipping in the Weddell case study slightly more informative priors were used for $\vartheta_{2}$. To assess model convergence, the chains of the primary movement parameters were visually inspected to detect poor mixing and excessive autocorrelation and the Gelman‐Rubin diagnostic (potential scale reduction factor $\hat{r}$) values checked (these should be less than ~1.1; Gelman & Rubin, 1992).

## 3 Results

For both case studies, the results are presented for (i) the original two‐state behavioral switching model, (ii) the unaugmented three‐state model that is informed solely by horizontal location data, and (iii) the three‐state models incorporating ancillary tag information (i.e., the “haulout” and “activity” models incorporating data on haulout status and vertical diving activity, respectively). For completeness, the model outputs are comprehensively documented in Appendix S4.

### 3.1 Case study 1: Weddell seals

For the Weddell seal case study, no major convergence problems were diagnosed for the three model formulations from either the $\hat{r}$ diagnostics (Appendix S3, Table S3.2), nor inspection of the MCMC chains (Appendix S4), although there was some indication of poor mixing in the γ estimates from the unaugmented three‐state model (Appendix S4, Fig. S4.2 P3). The general properties of the estimated state movement parameters ($\vartheta_{i}$ and $\gamma_{i}$) were similar between the unaugmented three‐state and haulout models (Appendix S4, Figs S4.2 and S4.3 P5).

**Figure 1:**
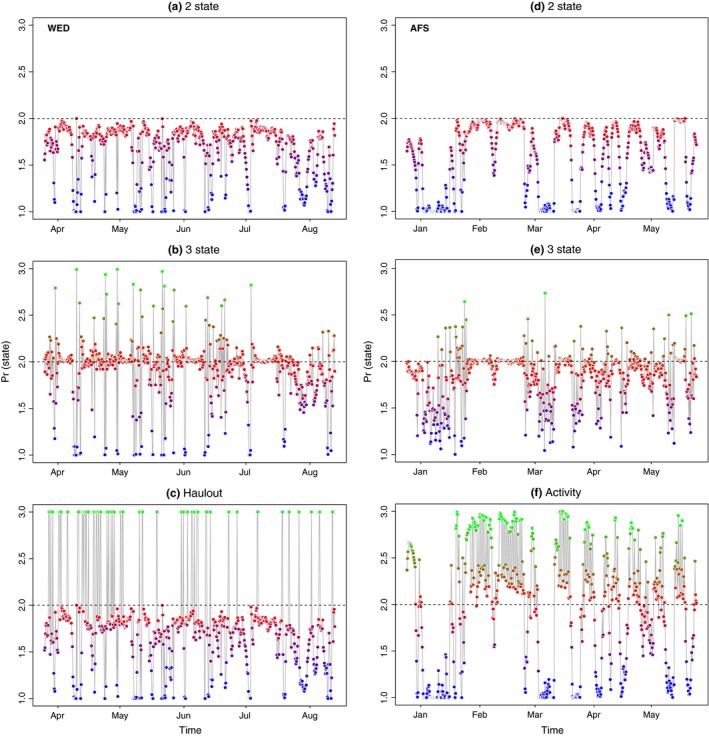
Example time‐series of behavioral state estimates for the Weddell (a–c) and Antarctic fur seal (d–f) case studies. The posterior means are presented for the (a, d) two‐state, (b, e) three‐state and (c, f) three‐state augmented models (haulout and activity, respectively). Colors are scaled from blue (1 = “directed”), through red (2 = “resident”) to green (3 = “haulout/inactive”). Time‐series for a single individual seal are shown in each case for clarity (WED896 and AFS07), but the full results are available in Appendix S4

**Figure 2:**
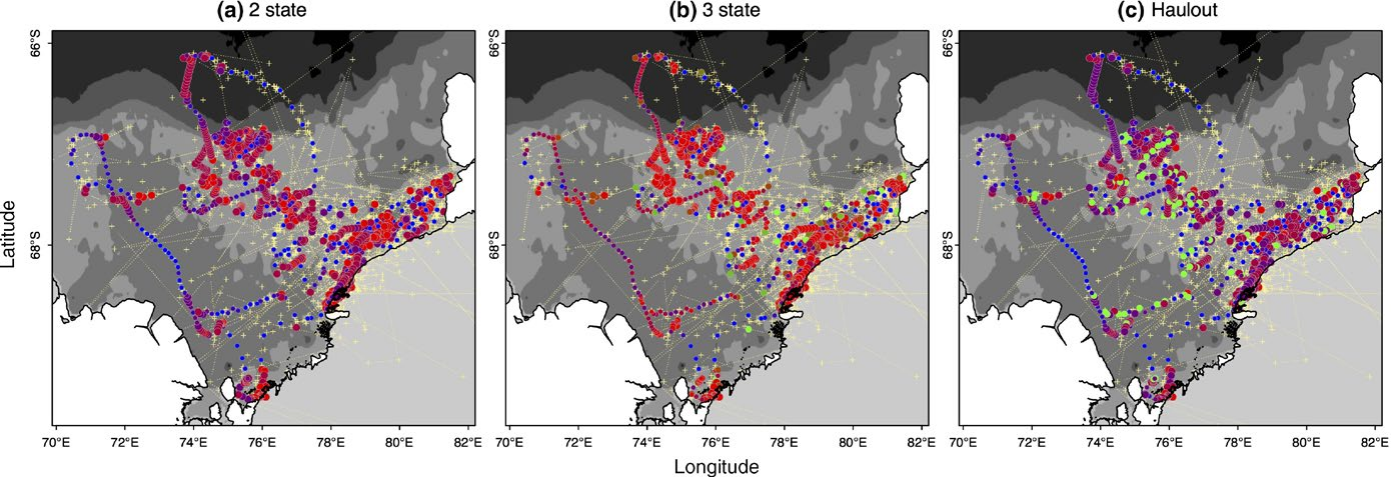
Map of estimated positions and inferred behavioral states for the Weddell seal (*N *=* *7) case study. Results are presented from the (a) two‐state, (b) three‐state, and (c) haulout models. Positions are colored as in Figure 1. For clarity, only three individual seals are shown (WED IDs 880, 882 and 896), but the full results are available in Appendix S4. The Antarctic continent is shown in gray, Antarctic coastline in black, and positions of the major ice shelves in white. Bathymetric contours are at 500 m, 1,000, 2,000, and 3,000 m depth (gray shading). Yellow crosses connected by a dotted line indicate the irregular Argos observations

Comparison of the allocation of behavioral states, relative to the known behavior from the ancillary data, clearly showed the unaugmented three‐state model mischaracterized the major proportion of known haulout time steps (Table 1). Examination of the estimated state time‐series (Figure 1a,b) and the spatial distribution of these (Figure 2a,b) indicated a portion of time steps, previously characterized as resident under the simplest two‐state model, were fairly evenly redistributed among the other two states (see also Appendix S4, Figs S4.1 and S4.2 P1‐2); in fact, these largely comprised those time steps with lowest haulout probabilities (i.e.*,* less than 0.45, Table 1). Because the ancillary information is prioritized in the haulout model formulation, this extra level of behavioral complexity was represented without mischaracterization (Table 1). The behavioral time‐series and mapped behavioral states subsequently showed both good maintenance of directed and resident behavioral characterization and correct allocation of systematic haulout activity (Figures 1c and 2c and Appendix S4 Fig. S4.3 P1‐2). If deviance information criteria (DIC) values were to be used as a guide for model selection (Table 1), then the addition of the ancillary haulout information implies an improvement in model fit relative to the unaugmented three‐state model.

**Table 1:** Model assessment: deviance information criteria (DIC) and the characterization of behavior in relation to observed proportions

					Percentage (%) of time steps								
Weddell seals					Pr(h) = 0–0.45			Pr(h) = 0.45–0.55			Pr(h) = 0.55–1		
Observed					81.8%			2.1%			16.1%		
Modeled	*pD*	$\overset{¯}{D}$	DIC^a^	ΔDIC	s1	s2	s3	s1	s2	s3	s1	s2	s3
Two state^b^	4,585	−49,968	−45,383	38	17.2	64.5	NA	0.2	2.0	NA	1.4	14.7	NA
Three state	4,576	−49,958	−45,382	39	26.7	44.2	10.8	0.4	1.6	0.2	3.8	10.1	2.2
Haulout	4,581	−50,002	−**45,421**	0	17.9	63.9	0	0.1	1.0	1.1	0	0	16.1

Antarctic fur seals					Pr(i) = 0–0.45			Pr(i) = 0.45–0.55			Pr(i) = 0.55–1		
Observed					48.8%			10.8%			40.4%		
Modeled					s1	s2	s3	s1	s2	s3	s1	s2	s3
Two state	3,273	−40,946	−**37,673**	0	19.9	28.9	NA	5.3	5.5	NA	13.0	27.3	NA
Three state	3,277	−40,931	−37,654	18	21.1	25.8	1.9	5.3	5.0	0.5	12.7	25.0	2.7
Activity	3,277	−40,939	−37,662	11	21.6	27.1	0.1	5.9	3.1	1.9	12.8	0	27.5

a The DIC (Spiegelhalter, Best, Carlin, & van der Linde, 2002) is calculated from $\overset{¯}{D}$ + *pD* and can be used to compare the fit of the models; like AIC, BIC, and similar criterion, lower values indicate better fit. The *pD* value is the effective number of parameters and is used to penalize models with more parameters. $\overset{¯}{D}$ is the posterior mean of the deviance.

b States refer to: directed (state 1), resident (state 2), and haulout/inactive (state 3).

Lowest DIC values are shown in bold.

The ΔDIC column (column four in upper and lower part of table) will then line up properly underneath each other.

Pr(h), Pr(haulout); Pr(i), Pr(inactive).

### 3.2 Case study 2: Antarctic fur seals

**Figure 3:**
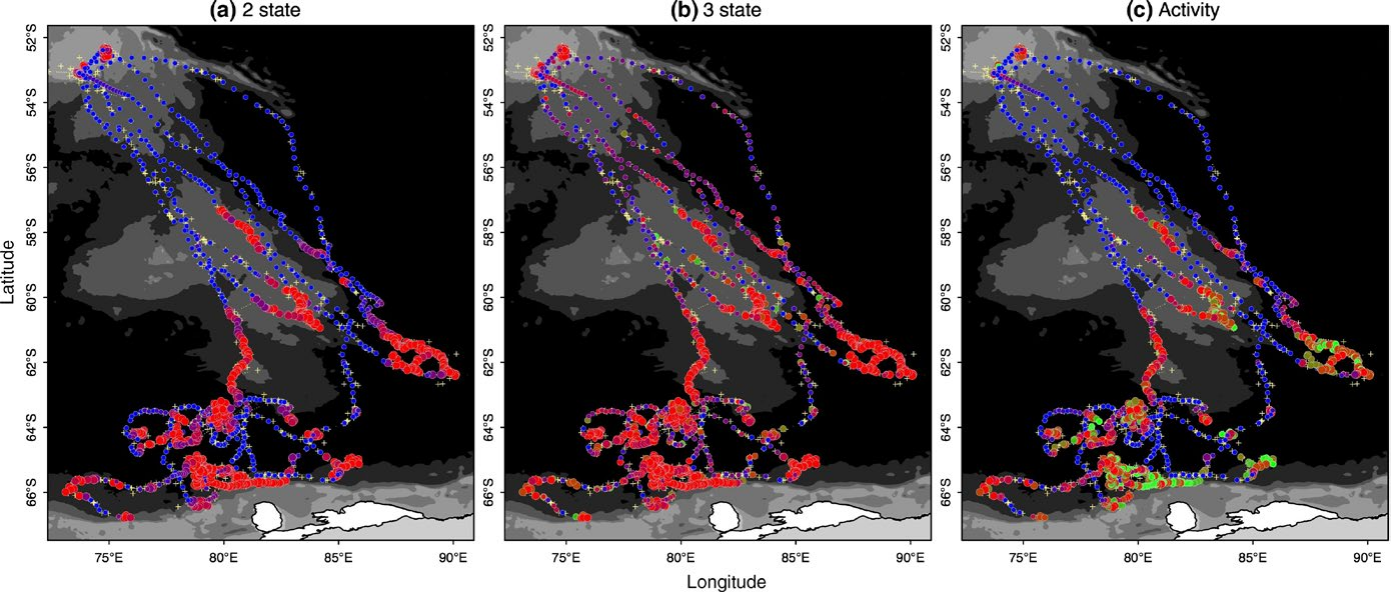
Map of estimated positions and inferred behavioral states for the Antarctic fur seal (*N *=* *5) case study. Results are presented as in Figure 2 from the (a) two‐state, (b) three‐state, and (c) activity models

There was a clear lack of convergence in the unaugmented three‐state model for the AFS case study. Diagnostics for this model formulation showed $\hat{r}$ values well above 1.1 (Appendix S3, Table S3.2) specifically in the $\gamma_{1}$, $\gamma_{3}$, and $\vartheta_{3}$ parameters, and nonconvergence was visible in these MCMC chains (Appendix S4 Fig. S4.5 P3‐4). The state time‐series estimates (Figure 1e) and the mapped distributions (Figure 3b) showed a very low allocation of state 3 (inactive) behavior overall (see also Appendix S4, Fig. S4.5 P1‐2) and also that directed behavior was very poorly defined in comparison with the original two‐state model (Figures 1d and 3a).

The “activity” model, informed by the ancillary dive data, discriminated all three states well with periods of diving inactivity regularly interspersed within the resident state (Figures 1f and 3c). Characterization of the state three inactivity did not suffer from false‐positive mischaracterization (Table 1) with the majority of known inactive periods clearly and correctly allocated. Note here again that diving inactivity within directed periods (12.8%) is permitted and classified as state 1 (see Methods). If the DIC values were to be used as a guide, the original two‐state model provided the apparently best fit (Table 1). As the initial objective was to discriminate activity status at apparently high‐focus residential locations, model evaluation may be better addressed based on improvements in state characterization, which may not necessarily correspond to improvements in fit according to information criterion.

### 3.3 Movement state properties

Compared to the directed (state 1) movements, the resident (state 2) and haulout/inactive (state 3) movements exhibited substantially less movement persistence and higher turn angles for both case study implementations. However, the estimated movement parameters were more strongly divergent in the AFS case study.

**Figure 4:**
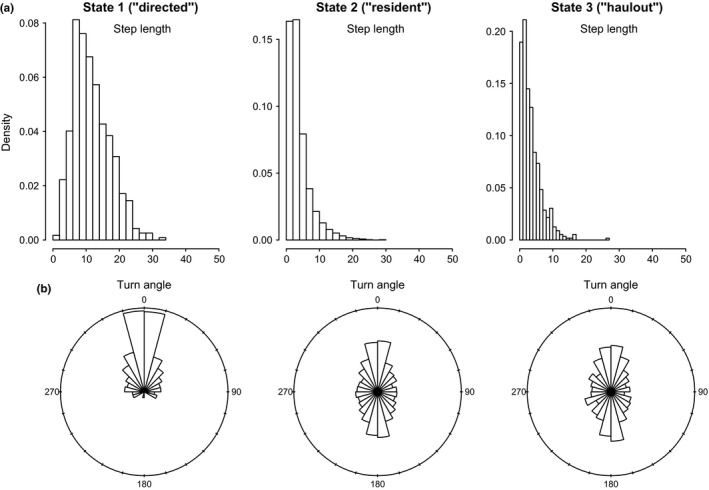
(a) Step length (km per 6‐h time step) and (b) turn angle (0–360°) distributions calculated from the most probable fitted tracks in three behavioral states (directed, resident, and inactive) inferred using the “haulout” model for the Weddell seal case study. These represent the actual modeled move displacements and turn angles which resulted from the correlated random walk process as governed by the state movement parameters (γ and ϑ; these parameter distributions are given in Table 2 and displayed in Appendix S4)

In the haulout model for Weddell seals, the directed movement state was characterized by relatively high estimated persistence values (posterior median for $\gamma_{1}$ = 0.72 (0.66–0.79); 95% highest posterior density interval [HPDI]) and mean turn angles close to zero (Table 3); the persistence parameter estimates steadily declined in the resident and haulout states, and these were both characterized by high mean turn angles close to π (i.e.*,* 180°, Table 2; see also Appendix S4, Fig. S4.3 P5). High persistence values translate to greater movement displacements, that is, greater speed of travel. Step length distributions (Figure 4a) can be empirically derived by application of great circle distance calculations to the resultant most probable fitted tracks, which subsequently gave mean displacements of 11.72 ± 5.52 km per 6‐h time step in the transit state, compared with 3.91 ± 3.51 km and 3.54 ± 3.14 km in the resident and haulout states, respectively. The actual turn angles per modeled move step, also derived by calculation from the most probable fitted tracks, were as expected close to zero in transit, whereas in the other two states these showed a high tendency to reverse direction but also a high variance around this tendency (Figure 4b).

**Table 2:** Posterior distributions of the movement parameters (persistence: $\gamma_{i}$; and turn angle: $\vartheta_{i}$) in three behavioral states (directed, resident, and inactive) inferred using the haulout and activity models (see Methods) for the Weddell and Antarctic fur seal case studies, respectively. Results present the posterior median (lower–upper 95% highest posterior density interval, HPDI)

Species		1. Directed	2. Resident	3. Haulout/Inactive
Weddell seal	$\gamma_{i}$	0.72 (0.66 to to 0.79)	0.48 (0.40 to 0.55)	0.24 (0.15 to 0.34)
	$\vartheta_{i}$	0.01 (−0.05 to 0.03)	3.16 (3.09 to 3.22)	3.04 (2.82 to 3.21)
Antarctic fur seal	$\gamma_{i}$	0.85 (0.81 to 0.89)	0.04 (0.00 to 0.19)	0.04 (0.00 to 0.13)
	$\vartheta_{i}$	−0.06 (−0.09 to −0.02)	2.84 (0.37 to 5.86)	0.56 (−2.08 to 2.40)

**Table 3:** Estimated time allocation budgets for Weddell and Antarctic fur seal case studies. Results are shown as the median proportion of 6‐h time steps (Weddell: *N *=* *3,264, AFS: *N *=* *1,534) assigned to three movement behavior states (lower–upper 95% HPDI). The species case studies use the haulout and activity model formulations, respectively, which each handle periods of behavioral inactivity differently (see Methods). In each case, the third state represents individual animals being hauled out of the water, or being in the water but nondiving, respectively. NA indicates not applicable

Species	Model	Behavioral state time allocation		
		1. Directed	2. Resident	3. Haulout/Inactive
Weddell seal	Two state	0.31 (0.27–0.35)	0.69 (0.65–0.73)	NA
	Three state	0.30 (0.25–0.35)	0.48 (0.41–0.55)	0.22 (0.17–0.28)
	Haulout	0.29 (0.25–0.33)	0.54 (0.50–0.58)	0.17 (0.17–0.17)^a^
Antarctic fur seal	Two state	0.43 (0.37–0.48)	0.57 (0.52–0.63)	NA
	Three state^b^	0.34 (0.24–0.46)	0.48 (0.35–0.57)	0.17 (0.11–0.25)
	Activity	0.42 (0.38–0.48)	0.22 (0.20–0.25)	0.35 (0.31–0.39)

a Recall first reference is made to the ancillary data to define in the haulout model so this state is discriminated without error (see Methods).

b Nonconvergence between chains, refer to Appendix S3, Table S3.1 for Gelman‐Rubin diagnostics (potential scale reduction factors, $\hat{r}$).

**Figure 5:**
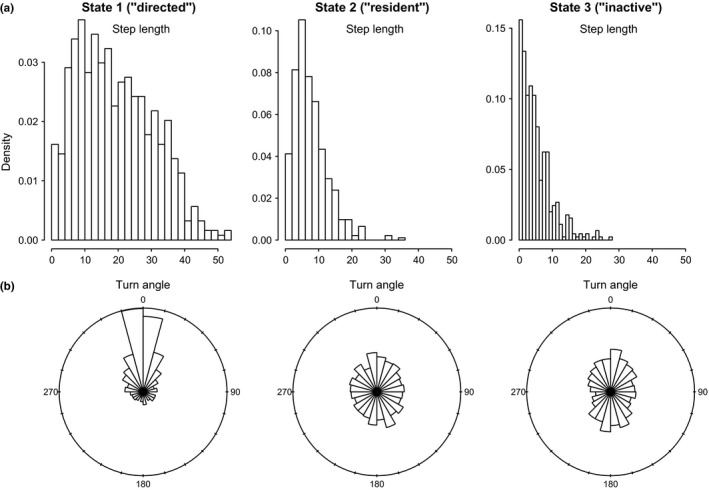
(a) Step length (km per 6‐h time step) and (b) turn angle (0–360°) distributions, calculated as in Figure 4, for three behavioral states (directed, resident, and inactive) inferred using the “activity” model for the Antarctic fur seal case study

In the AFS case study, the directed state persistence parameter was substantially higher ($\gamma_{1}$ = 0.85 (0.81–0.89 HPDI)) and extremely low during the resident and inactive states ($\vartheta_{2}$ and $\vartheta_{3}$ ~ 0.04) (see Table 2 and also Appendix S4, Fig. S4.6 P5). Similarly to Weddell seals, the $\vartheta_{i}$ estimates were close to zero; however, the remaining $\vartheta_{i}$ parameters were not well resolved and remained relatively unconstrained. The AFS empirically derived step lengths (SL) were therefore higher than in the Weddell case study (SL_1_ = 19.22 ± 11.36; SL_2_ = 7.66 ± 5.10; SL_3_ = 5.16 ± 4.67 km per 6‐h time step; see Figure 5a). The derived turn angles during transit were again close to zero but, consistent with the unconstrained $\vartheta_{i}$ estimates, were highly variable for the nontransit states and freely spanned the available range 0–2π (i.e.*,* 0–360°, Figure 5b).

Some differences were found with respect to state transition probabilities although, as the ancillary behavioral data influenced these, not all are informative (Appendix S3, Table S3.3). Weddell seals, however, were highly likely to remain in the resident state $\varphi_{2,2}$ = 0.81 (0.75–0.86) and the switch from transit into residency occurred reasonably often $\varphi_{1,2}$ = 0.34 (0.26–0.41). Similarly, switches into transit state occurred with reasonable frequency ($\varphi_{i,1}∼0.19$). In contrast, the parameter estimates for these switch rates were very low for AFS, likely reflecting the nature of their long migration path (Figure 3). Switches between resident and inactive (nondiving) states, as determined by the ancillary tag data, occurred frequently (Table 3 and Figure 1f). With a 6‐h model time step, the inactive periods show a consistent correlation at lag 4 and a high periodicity at 0.25, strongly indicative of a diurnal cycle (Appendix S3, Fig. S3.1). While the time step is relatively coarse, a comparison with solar position showed 86% of the inactive time steps occurred during day/dawn periods compared with only 14% during dusk/night periods and this is consistent with expectations that AFS are predominantly nocturnal foragers.

### 3.4 Time budget allocation

As expected, substantial differences in the overall time allocation budgets were estimated for the two case studies depending on the specific movement process model. Moving from the simpler two‐state switching model to an augmented three‐state formulation reduced the estimated proportion of time spent in resident state from 0.69 (0.65–0.73 HPDI) to 0.54 (0.50–0.58 HPDI) in the Weddell seal case study (Table 3). Haulout accounted for 0.17 of the overall Weddell seal time budget. The drop in resident time was more dramatically pronounced in the AFS case study, from 0.57 (0.52–0.63 HPDI) to 0.22 (0.20–0.25 HPDI), with 0.35 (0.31–0.39 HPDI) of time spent in the inactive (nondiving) state. Although the prioritization of behavioral data (“haulout” model) or movement data (“activity” model) differed between the model formulations, the overall time estimated in directed state remained relatively stable in both cases: near 0.30 for Weddell seals and slightly above 0.40 for AFS (Table 3). The comparative results indicated AFS spent a much greater portion of time in the inactive and also directed states than Weddell seals and a much smaller portion of time in the resident state overall.

## 4 Discussion

Biotelemetry studies tracking cryptic or migratory species often collect detailed behavioral information in conjunction with location data (Hussey et al., 2015). This presents a real, but largely underutilized, opportunity to extend modeling frameworks for animal movement to capture more biologically meaningful processes. The two model formulations we present here incorporate ancillary information collected by tags about haulout and diving behavior in two seal species. This methodology could be implemented with any other source of data that may usefully serve to inform behavioral state. For example, increasingly sophisticated biotelemetry applications can provide direct information about prey capture attempts (Carroll, Slip, Jonsen, & Harcourt, 2014) or indirect information on internal state condition such as lipid gain (Biuw et al., 2007). Our results highlight that simple but important changes in inference of movement state and subsequent activity budgets can arise by incorporating behavioral data. Model frameworks that combine multiple sources of information will clarify relationships between movement decisions and fitness, and ultimately can be applied to monitoring changes in habitat and prey resources at the land‐ or seascape level.

Weddell seals provide an appealing case study for a number of reasons relating to their ecology. As a highly residential and ice‐obligate species that are rarely recaptured, Weddell seals typically provide satellite tracking data that are difficult to interpret, given both the position error scales relative to their movement scales and their tendency to frequently haulout on sea ice and remain relatively stationary. Our results confirmed a large proportion of time steps were mischaracterized when three behavioral states were inferred from horizontal trajectory alone. However, very good results were obtained in resolving this extra behavioral complexity with the support of the ancillary haulout data.

Many modern marine telemetry studies focus on elucidating where and how marine predators successfully forage in a dynamic and heterogeneous three‐dimensional ocean environment (Block et al., 2011; Hussey et al., 2015; Raymond et al., 2014). Of increasing importance is how these forage resources are likely to change in the future (Hazen et al., 2013). In many cases, species are operating within the confines of both physical and physiological constraints, for example, being physically limited by land‐based breeding and physiologically limited by their diving capacity. In the case of Weddell seals, the dynamic ice habitat adds a further layer of complexity. Availability of suitable fast‐ice substrate for resting, molting, and breeding is likely to be of first‐order importance in developing habitat suitability distribution models (Raymond et al., 2014). Availability of forage areas suitably proximate to fast ice then becomes a necessity for survival. Being able to more accurately discriminate between resident (nominally forage) and inactive/haulout behavior makes it possible to identify key ice areas in regular use for either purpose and presents the opportunity to evaluate the future status of these areas under specific climate change scenarios.

The Antarctic fur seal case study also demonstrated that activity status is likely to have important implications for activity budgets and, potentially, broader efforts to estimate food consumption by marine predators in the Southern Ocean and elsewhere. All of the southern fur seals were historically hunted almost to extinction, but Antarctic fur seal populations are now considered fully recovered with many still increasing (Committee 2011). The species feeds mainly on Euphausiids, including Antarctic and other krill species, as well as a variety of fishes, including icefishes (Channichthyidae and Nototheniidae) and lanternfishes (Myctophidae) (Raymond et al., 2011). Because AFS are dependent on prey targeted by commercial Southern Ocean fisheries (i.e.*,* Antarctic krill and icefish), they are consequently monitored by the Convention for the Conservation of Antarctic Living Marine Resources (CCAMLR) Ecosystem Monitoring Program (CEMP). Attempts to estimate food consumption are central to defining the ecological role of marine predators (Boyd, 2002; Southwell, Emmerson, Forcada, & Southwell, 2015) and assessing predator–prey fishery interactions. This is particularly a priority in CCAMLR Subareas 48.1 and 48.2 near the Antarctic Peninsula where the krill fishery is concentrated.

Consumption estimates (Boyd, 2002; Southwell et al., 2015) rely on many uncertain demographic (e.g.*,* survival rate and offspring production) and individual‐level parameters, one of which is the measurement of metabolic rate. Metabolic rate is challenging to measure directly in wild animals (Iverson, Sparling, Williams, Lang, & Bowen, 2010) and has not been measured directly in male AFS. The daily gross energy requirement of an individual varies across particular stages of the annual cycle (e.g.*,* breeding ashore, at sea, molting) and is generally expected to be high (i.e., using some multiplier above baseline) while at sea. Our results clearly demonstrate that AFS energetic costs are likely to vary among at‐sea movement states, with the highest costs expected during transit but potentially significant energy savings expected during diurnal inactivity while on their forage grounds. Movement rates near 0.24 m/s are consistent with rates that might be expected from passive drift (Meijers, Klocker, Bindoff, Williams, & Marsland, 2010), and at least some of these inactivity periods are likely to involve use of sea ice for haulout, as in Weddell seals. Ignoring reduced energy expenditure from these movement switches between activity/inactivity may lead to a gross positive bias in field metabolic rate and the estimate of the mean amount of food consumed (Boyd, 2002).

The approach outlined here has applications well beyond the case studies presented. Conservation and management of seabird interactions with fisheries would very likely benefit from incorporating ancillary tag information into modeling of at‐sea activity budgets. Recent attempts have quantified the influence of fishing activity (vessel distance, type, and activity: drifting, fishing, or steaming) on state‐switching probabilities of Northern gannets (*Morus bassanus*) between “commuting” and “foraging” behaviors (Bodey et al., 2014). Ancillary tag information, from miniature saltwater immersion switches and/or TDRs (Weimerskirch, Corre, Jaquemet, & Marsac, 2005), can further discriminate the time engaged in active foraging from periods of inactivity when birds are sitting on the sea surface (Dean et al., 2013). By combining movement and activity data from multiple loggers to provide finer scale estimation of at‐sea behaviors, there is a high likelihood of improved quantification of spatiotemporal patterns in active fishing interactions. Moreover, further practical applications include distinguishing specific behavioral characteristics and their timing, such as nocturnal patterns in at‐sea resting or other environmental associations with periods of high landings and takeoffs, all of which can contribute toward developing seabird‐vessel interaction and bycatch mitigation strategies via altered timing and practice of setting and/or hauling fishery gear (Bull, 2007; Robertson, Candy, Wienecke, & Lawton, 2010).

Our approach is similar in concept to that of (McClintock et al. 2015; McClintock et al., 2013) but relies on a more simplistic incorporation of ancillary tag information. McClintock et al. (2013, 2015) formally incorporate ancillary tag information as an additional likelihood component with additional distributions and parameters that inform behavioral state switching. Our approach directly uses the proportion of time step hauled out or diving to determine the probability of being in the “haulout” or “inactive (nondiving)” state. Our behavioral state estimation has a relatively straightforward hierarchy: in the haulout model, Weddell seals can only transition into the directed or resident state if they are not hauled out, and in the activity model AFS can only transition between inactive (nondiving) and active (diving) states when they are in the resident state. These configurations are suitable for the specific cases treated here, yet it is conceivable that cases may arise with multiple types of ancillary data, for which a more complicated hierarchical structure might be designed. Else, the more generalized approach of McClintock et al. (2013, 2015), treating the ancillary data similarly to the location data, that is, as random variables, could be implemented. Information about vertical movements is one of the most common ancillary data types collected in studies of aquatic movement ecology (Hussey et al., 2015). Furthermore, the SMRU‐SRDL tags documenting haulout, diving, and other behavioral summaries (Photopoulou, Fedak, Matthiopoulos, McConnell, & Lovell, 2015) are being ever more widely deployed across species including seals (Hindell et al., 2016), narwhals (Lydersen, Martin, Gjertz, & Kovacs, 2007), salmon sharks (Block et al., 2011), turtles (Benson et al., 2011), sea lions (Lowther, Harcourt, Page, & Goldsworthy, 2013), and walruses (Beatty et al., 2016). The approach presented here may be easily up‐taken and fit by ecological users across a range of appropriate data sets.

This methodology can be readily implemented with any source of ancillary data that can reasonably be expressed as a proportion (or binary) indicator to inform behavioral state. Irregular location observations typically have a temporal mismatch with tag‐recorded ancillary behavioral data. In the examples presented here, the time step of the modeled CRW was selected to match the temporal resolution (6 h) of the available ancillary data, and to be adequate to represent the movement process. However, the choice of time step exercised by the user should simply be reasonable given the (1) temporal resolution of the available data (location and activity), and (2) temporal scale over which the behaviors are expected to occur. For example, consider a terrestrial species with more frequent and precise location observations (e.g., GPS) together with high‐resolution accelerometry data (Mosser, Avgar, Brown, Walker, & Fryxell, 2014). Within the same SSM framework, the ecologist would update the observation model to use the scale of location error appropriate for the telemetry device, and select a short time interval (e.g., 15‐min time step) appropriate to the fine‐scale nature of the observed movement and activity. Accelerometry is itself a burgeoning discipline warranting new analytical methods (Carroll et al., 2014; Leos‐Barajasa et al., 2016; Nathan et al., 2012), but useful summaries can be made. For example, a proportional index (0–1) for how much “active” time (Mosser et al., 2014) was recorded, or how much a specific activity such as grazing occurred (Yoshitoshi et al., 2013), per time interval. Alternately a binary index (0 or 1) such as whether head jerks, indicative of prey capture attempts, occurred during each time interval (Ydesen et al., 2014). These indices could be directly incorporated within the existing SSM framework as an ancillary data input; or more complex parameterizations (e.g., modeling the actual number of head jerks) explored.

While our study represents a contribution toward modeling more realistic movement processes, it is obvious that animals can have complex ecologies. Flexible approaches that aim to detect an arbitrary number of behavioral shifts may therefore be appealing (Gurarie, Andrews, & Laidre, 2009; Nams, 2014). However, it is worthwhile re‐emphasizing here a clear distinction between differing approaches for analyzing animal movement data. The models provided here, and similar (Forester et al., 2007; Langrock et al., 2012; McClintock et al., 2013; Ovaskainen, 2004; Patterson et al., 2008), explicitly describe movement mechanisms via a stochastic process model. While these are simplified relative to the true biology, they nonetheless rely on biologically meaningful parameters which may be estimated from data. Further, they neatly deal with many of the key statistical issues plaguing analysis of movement data, such as temporal and spatial dependence and errors in location data. The alternative approaches do not provide an explanatory model but an empirical, descriptive distillation of movement data (Gurarie et al., 2009) often requiring biological inference to be conducted post hoc. These empirical approaches are less powerful in their ability to aid direct inference about movement processes but may provide useful synergies in motivating the development of improved mechanistic models.

Our study adds to a growing body of literature working toward inference about animal behavioral states based upon more than just horizontal movement information. Modern biotelemetry instruments now collect a wealth of information about animal behavior, physiology, and the surrounding ocean environment that all provide valuable context about individual space use. Analytical methods that harness this contextual information can provide improved inference of animal movement ecology at individual to population levels. Greater biological realism in movement models should facilitate more robust quantification of environmental and biotic factors predictive of movement behavior, and the implications for foraging success, energy budgets, and more broadly current and future habitat dependencies for key life‐history stages. Movement ecology is fundamental to understanding the role marine predators play within ocean ecosystems, and identifying synthetic patterns across guilds as well as keying in on the unique niches and dependencies exhibited by particular species.

## Conflict of Interest

None declared.

## Data Accessibility

Data from this analysis are available in Supporting Information (Data S1).
