# Supplementary material for: Putting the behavior into animal movement modeling: Improved activity budgets from use of ancillary tag information
Source: Ecol Evol. 2016 Oct 20;6(22):8243–55. doi: 10.1002/ece3.2530 (PMC5108274; doi:10.1002/ece3.2530)
Supplement: Supplementary file 1 [file ECE3-6-8243-s001.pdf]

Appendix S1: State-space formulation of movement models

We model movement as a compound correlated random walk (CRW) that can be decomposed into two or more discrete behavioural states (Morales *et al.*, 2004; Jonsen *et al.*, 2005). Here we describe the basic two-state model originally published in Jonsen *et al.* (2005).

We model two behavioural states: a 'resident' (R) state consisting of relatively slow movements with frequent course reversals and a 'directed' (D) state consisting of relatively fast and more directionally persistent movements. The model is a first-difference CRW that includes stochastic switches between behavioural states, where the states are defined as unique combinations of two movement parameters: the mean turn angle ϑ_{b_t} and the move persistence γ_{b_t}. The subscript b_t denotes the behavioural state at time t, where $b = 1$ or 2. The model has the general form:

$$\mathsf{x}_t = \mathsf{x}_{t-1} + \gamma_{b_t}\mathsf{T}\left(\mathsf{x}_{t-1} - \mathsf{x}_{t-2}\right) + \mathsf{N}(0, \mathbf{\Sigma}) \tag{0.1}$$

where x_t and x_{t-1} are the unobserved true locations of an animal at times t and $t-1$. T is a matrix describing the mean turn angle, ϑ_{b_t}, between displacements $\mathsf{x}_t - \mathsf{x}_{t-1}$ and $\mathsf{x}_{t-1} - \mathsf{x}_{t-2}$:

$$\mathsf{T} = \begin{bmatrix} \cos\vartheta_{b_t} & \text{-}\sin\vartheta_{b_t} \\ \sin\vartheta_{b_t} & \cos\vartheta_{b_t} \end{bmatrix} \tag{0.2}$$

and $\mathbf{\Sigma}$ is a variance-covariance matrix specifying the magnitude of stochasticity in the 2-dimensional movements:

$$\mathbf{\Sigma} = \begin{bmatrix} \sigma^2_{\text{lon}} & \rho\sigma_{\text{lon}}\sigma_{\text{lat}} \\ \rho\sigma_{\text{lon}}\sigma_{\text{lat}} & \sigma^2_{\text{lat}} \end{bmatrix} \tag{0.3}$$

Switching between behavioural states is governed by a Markov chain with fixed transition probabilities:

$$\Pr(b_t = i | b_{t-1} = j) = \varphi_{ji} \tag{0.4}$$

where φ_{ji} is the probability of switching from behavioural state j at time $t-1$ to behavioural state i at time t. In a 2-state context the φ_{ji}'s are elements of a 2 x 2 transition matrix:

$$\boldsymbol{\varphi} = \begin{bmatrix} \Pr(\mathsf{D}_t|\mathsf{D}_{t-1}) & \Pr(\mathsf{R}_t|\mathsf{D}_{t-1}) \\ \Pr(\mathsf{D}_t|\mathsf{R}_{t-1}) & \Pr(\mathsf{R}_t|\mathsf{R}_{t-1}) \end{bmatrix} \tag{0.5}$$

where φ_{11} and φ_{22} are the probabilities of remaining in the directed and resident states, respectively. φ_{12} and φ_{21} are the probabilities of switching from the directed to the resident state and from the resident to the directed state, respectively. These transitions can be estimated assuming a first-order Markov categorical distribution. In practice, only φ_{11} and φ_{21} need to be estimated as the rows of $\boldsymbol{\varphi}$ must sum to 1.

Location uncertainty is accounted for via the observation model. Due to the irregular sampling of locations that is inevitable with many telemetry data types, especially Argos telemetry data, the observation model includes a regularisation that links the irregularly timed observations to the states that occur regularly through time (Jonsen *et al.*, 2005). Here we present an observation model for Argos telemetry data, where the errors in observed locations vary according to a location class descriptor provided by the Argos system (Vincent *et al.*, 2002; Jonsen *et al.*, 2005; Costa *et al.*, 2010). This approach can be modified to accomodate location observations from other telemetry platforms.

$$\mathsf{y}_i = \boldsymbol{\mu}_i + t(0, \psi\boldsymbol{\tau}_{q,i}, \boldsymbol{\nu}_{q,i}) \tag{0.6}$$

where y_i is the ith $(i = 1, \ldots, n)$ observed location with the time interval $t-1$ to t, $\boldsymbol{\mu}_i$ is an estimate of the corresponding true location, $\boldsymbol{\tau}_{q,i}$ and $\boldsymbol{\nu}_{q,i}$ are the scale and df parameters, from a generalised t-distribution, for Argos location class q $(q = 1, \ldots, 6)$ associated with the ith observation. The parameter ψ is estimated to scale the $\boldsymbol{\tau}_{q,i}$'s, accounting for potential differences in performance between tags. The $\boldsymbol{\mu}_i$'s were derived from the estimated location states x_t via:

$$\boldsymbol{\mu}_i = (1 - j_i)\mathsf{x}_{t-1} + j_i\mathsf{x}_t \tag{0.7}$$

where j_i $(0 < j_i < 1)$ is the proportion of the time step between location states x_{t-1} and x_t that elapsed prior to the ith observation. This approach assumes the seals travel in a straight line between times $t-1$ and t.

References

Costa, D. P., Robinson, P. W., Arnould, J. P. Y., Harrison, A.-L., Simmons, S. E., Hassrick, J. L., Hoskins, A. J., Kirkman, S. P., Oosthuizen, H., Villegas-Amtmann, S. & Crocker, D. E. (2010). Accuracy of argos locations of pinnipeds at-sea estimated using fastloc gps. *PLoS ONE*, 5, e8677. URL http://dx.doi.org/10.1371%2Fjournal.pone.0008677.

Jonsen, I. D., Flemming, J. M. & Myers, R. A. (2005). Robust state--space modeling of animal movement data. *Ecology*, 86, 2874--2880. URL http://dx.doi.org/10.1890/04-1852.

Morales, J. M., Haydon, D. T., Frair, J., Holsinger, K. E. & Fryxell, J. M. (2004). Extracting more out of relocation data: Building movement models as mixtures of random walks. *Ecology*, 85, 2436--2445. URL http://dx.doi.org/10.1890/03-0269.

Vincent, C., McConnell, B. J., Fedak, M. A. & Ridoux, V. (2002). Assessment of ARGOS location accuracy from satellite tags deployed on captive grey seals. *Marine Mammal Science*, 18, 301--322.
