# Supplementary material for: Putting the behavior into animal movement modeling: Improved activity budgets from use of ancillary tag information
Source: Ecol Evol. 2016 Oct 20;6(22):8243–55. doi: 10.1002/ece3.2530 (PMC5108274; doi:10.1002/ece3.2530)
Supplement: Supplementary file 2 [file ECE3-6-8243-s002.docx]

**Appendix S2.** **JAGS code** for each state space model formulation (i) 2-state behavioural switching model, (ii) 3-state behavioural switching model, (iii) 3-state ‘haulout’ model, and (iv) 3-state ‘activity’ model.

**File list**

i_hDCRWS_WED.txt – 2-state behavioural switching model (Weddell case study)

i_hDCRWS_AFS.txt – 2-state behavioural switching model (AFS case study)

ii_hDCRW3S_WED.txt – 3-state behavioural switching model (Weddell case study)

ii_hDCRW3S_AFS.txt – 3-state behavioural switching model (AFS case study)

iii_hHaulout_WED.txt – 3-state ‘haulout’ model (Weddell case study)

iv_hActivity_AFS.txt – 3-state ‘activity’ model (AFS case study)
