# Supplementary material for: Putting the behavior into animal movement modeling: Improved activity budgets from use of ancillary tag information
Source: Ecol Evol. 2016 Oct 20;6(22):8243–55. doi: 10.1002/ece3.2530 (PMC5108274; doi:10.1002/ece3.2530)
Supplement: Supplementary file 3 [file ECE3-6-8243-s003.docx]

**Table S3.1. Prior distributions for movement process parameters.** The process model essentially follows that previously published (Jonsen et al 2003, 2005; Bestley et al 2013, 2015), with the exception of the use of Dirichlet priors for the switching probabilities. Subscript *i* denotes the *i*th movement state. To prevent state-flipping the prior distributions nominally constrain the move persistence in state 1 (‘directed’) to be highest $\gamma_{1}>\left[\gamma_{2},\gamma_{3}\right]$ ; additionally, in the Weddell case study slightly more informative priors were used for$\vartheta_{2}$, and in these 3-state models (noting ‘haulout’ is essentially stationary)$\gamma_{1}>\gamma_{2}>\gamma_{3}$. For the Weddell case study, the prior for $\vartheta_{3}$follows that of $\vartheta_{2}$to centre on *π* (i.e. consistent with the prior expectation of non-directed movement); for the AFS case study this expectation was unclear for the ‘inactive’ (non-diving) state 3 but preliminary tests showed improved MCMC chains for $\vartheta_{3}$to follow the prior of $\vartheta_{1}$and centre on zero.

| **Parameter** | **Interpretation** | **Prior distribution** | |
| --- | --- | --- | --- |
|  |  | **Weddell case study** | **AFS case study** |
| $\vartheta_{i}$ | Mean turn angle (radians) | $\vartheta_{1}\text{\textasciitilde}$Beta(10, 10), range -π:π  $\vartheta_{2}\text{\textasciitilde}$Beta(5, 5) , range 0:2π  $\vartheta_{3}\text{\textasciitilde}$Beta(2, 2) , range 0:2π | $\vartheta_{1}\text{\textasciitilde}$Beta(10, 10), range -π:π  $\vartheta_{2}\text{\textasciitilde}$Beta(2, 2) , range 0:2π  $\vartheta_{3}\text{\textasciitilde}$Beta(2, 2) , range -π:π |
| $\gamma_{i}$ | Move persistence: the degree of correlation in both move speed and direction | $\gamma_{1}\text{\textasciitilde}$Beta(2, 1.5)  $\gamma_{2}\text{\textasciitilde}\gamma_{1}*$Beta(1, 1)  $\gamma_{3}\text{\textasciitilde}\gamma_{2}*$Beta(1, 1) | $\gamma_{1}\text{\textasciitilde}$Beta(2, 1.5)  $\gamma_{2}\text{\textasciitilde}\gamma_{1}*$Beta(1, 1)  $\gamma_{3}\text{\textasciitilde}\gamma_{1}*$Beta(1, 1) |
| $\phi_{i,j}$ | The probability of switching from one movement state (*j*) into another (*i*) | $\begin{matrix} \varphi_{1,j} \sim\text{Dir}\left( 1,1,0 \right) \\ \varphi_{2,j} \sim\text{Dir}\left( 1,1,0 \right) \\ \varphi_{3,j} \sim\text{Dir}\left( 1,1,1 \right) \end{matrix}$ | $\begin{matrix} \varphi_{1,j} \sim\text{Dir}\left( 1,1,1 \right) \\ \varphi_{2,j} \sim\text{Dir}\left( 1,1,1 \right) \\ \varphi_{3,j} \sim\text{Dir}\left( 1,1,1 \right) \end{matrix}$ |
| $\Sigma$ | Process variance-covariance matrix (longitude, latitude) | $\Sigma\sim\text{W}^{-1}\left( \text{S}^{-1} = \mathbf{I}, 2 \right)$ where $\text{W}^{-1}$ is the Inverse-Wishart distribution, $\mathbf{I=}\left[ \begin{matrix} 1 & 0 \\ 0 & 1 \end{matrix} \right]$ | |

**Table S3.2. Potential scale reduction factors** $\left(\hat{r}\right)()$ calculated for the movement parameters $\left(\vartheta_{i}\text{ and }\gamma_{i}\right)$. Values are given as point estimates (upper confidence limits). Approximate convergence is diagnosed when the upper limit is close to 1; values indicative of convergence problems are shown in bold. NA indicates not applicable. We ran two MCMC chains with a burn in of 40 000 samples and 20 000 iterations thereafter, thinning to retain 1 in 20 samples. Hence the $\hat{r}$values are calculated based on a comparison of within-chain and between-chain variances using the 1000 samples retained from each chain.

| Species | Model | Movement | Multivariate | Individual $\hat{r}$per behavioural state | | |  |  |
| --- | --- | --- | --- | --- | --- | --- | --- | --- |
|  |  | parameter | $\hat{r}$ | 1. Directed | 2. Resident | 3. Inactive |  |  |
| Weddell seal | 2 state | $\vartheta_{i}$ | 0.999 | 1.00 (1.00) | 1.00 (1.00) | NA |  |  |
| (N = 7) |  | $\gamma_{i}$ | 1.01 | 1.01 (1.03) | 1.00 (1.02) | NA |  |  |
|  | 3 state | $\vartheta_{i}$ | 1.01 | 1.00 (1.01) | 1.01 (1.04) | 1.01 (1.01) |  |  |
|  |  | $\gamma_{i}$ | 1.01 | 1.02 (1.07) | 1.00 (1.00) | 1.01 (1.04) |  |  |
|  | Haulout | $\vartheta_{i}$ | 1.00 | 1.00 (1.01) | 1.00 (1.00) | 1.00 (1.01) |  |  |
|  |  | $\gamma_{i}$ | 1.03 | 1.03 (1.12) | 1.00 (1.02) | 1.01 (1.05) |  |  |
| Antarctic fur seal | 2 state | $\vartheta_{i}$ | 1.00 | 1.00 (1.00) | 1.00 (1.00) | NA |  |  |
| (N = 5) |  | $\gamma_{i}$ | 1.01 | 1.01 (1.04) | 1.00 (1.01) | NA |  |  |
|  | 3 state | $\vartheta_{i}$ | 1.03 | 1.06 (1.19) | 1.00 (1.02) | **1.24 (1.29)** |  |  |
|  |  | $\gamma_{i}$ | **1.32** | **1.49 (2.97)** | 1.01 (1.02) | **1.59 (3.05)** |  |  |
|  | Activity | $\vartheta_{i}$ | 1.01 | 0.999 (1.00) | 1.017 (1.06) | 1.003 (1.00) |  |  |
|  |  | $\gamma_{i}$ | 1.01 | 1.01 (1.03) | 1.01 (1.02) | 1.00 (1.01) |  |  |

|  |
| --- |

**Table S3.3. Estimated behavioural switching probabilities**$\left(\phi\right)$. Results are shown as the median (lower – upper 95% HPDI) probability of switching from one movement state into another. The two case studies use the haulout (WED) activity (AFS) model formulations, respectively (see Methods). NA indicates not applicable.

| Species |  | Behavioural state (*s_t_*) | | |
| --- | --- | --- | --- | --- |
|  | Behavioural  state (*s_t-1_*) | 1. Directed (‘transit’) | 2. Resident (‘forage’) | 3. Inactive (‘resting’) |
| *WED* | 1. Directed (‘transit’) | 0.66 (0.58 – 0.74) | 0.34 (0.26 – 0.41) | NA |
|  | 2. Resident (‘forage’) | 0.19 (0.14 – 0.25) | 0.81 (0.75 – 0.86) | NA |
|  | 3. Inactive (‘haulout’) | 0.19 (0.04 – 0.33) | 0.51 (**0.11 – 0.75**) | 0.29 (**0.01 – 0.85**) |
| AFS | 1. Directed (‘transit’) | **0.63** (**0.17 – 0.90**) | 0.06 (0.02 – 0.11) | **0.31 (0.02 – 0.81)** |
|  | 2. Resident (‘forage’) | 0.07 (0.01 – 0.16) | **0.65** (**0.13 – 0.90**) | **0.27 (0.01 – 0.86)** |
|  | 3. Inactive (‘resting’) | 0.02 (0.00 – 0.07) | **0.69** (**0.16 – 0.96**) | **0.28 (0.01 – 0.83)** |

**Figure S3.1.** **Time-series characteristics of the inactive (non-diving) state** estimated using the ‘activity’ model implemented for the Antarctic fur seal case study. Results show the autocorrelation function (LHS) and periodogram (RHS) computed for the behavioural inactivity time series. Panels show results for individual seals (*n* = 5). For 6h model time steps a consistent correlation at lag 4 and a high spectral density estimate at a frequency of 0.25 is indicative of typically one occurrence per 24h, i.e. a diurnal cycle.
