# Supplementary material for: Putting the behavior into animal movement modeling: Improved activity budgets from use of ancillary tag information
Source: Ecol Evol. 2016 Oct 20;6(22):8243–55. doi: 10.1002/ece3.2530 (PMC5108274; doi:10.1002/ece3.2530)
Supplement: Supplementary file 4 [file ECE3-6-8243-s004.docx]

**Appendix S4.** Full model results presented for each of the state space model formulations implemented in the Weddell (Figures S4.1 – S4.3) and Antarctic fur seal (Figures S4.4 – S4.6) case studies. Each multi-page PDF (created using the script hactssm.plt.r in Data S1) contains the following figures, in sequence:

**P1.** **Maps showing estimated positions and inferred behavioural states** for each individual seal included in the case study. Positions are colored as in Fig 2 (main manuscript), being scaled from blue (1 = ’directed/transit’), through red (2 = ‘resident/forage’) to green (3 = ‘haulout/inactive’). X-axis is Longitude (E) and y-axis is Latitude (S).

**P2.** **Time-series of behavioural state estimates** (posterior means) for each individual seal included in the case study. Colours are scaled as in Fig 2 and previous figure (P1). X-axis is Time and y-axis is behavioural state.

**P3. MCMC summary plots for the movement persistence parameters**$\gamma_{i}$ showing (LHS) the trace of the sampled output n = 1000 each from the two chains and (RHS) a density estimate for each parameter. Panel row number corresponds to the *i*th movement state i.e. row 1 = state 1 (directed/transit) etc. Plot created using library(rjags) (Plummer, 2015).

**P4. MCMC summary plots for the turn angle parameters** $\vartheta_{i}$ presented as in previous figure (P3).

**P5.** **Posterior distributions of the movement parameters** persistence: $\gamma_{i}$; and turn angle: $\vartheta_{i}$; (presented in upper and lower panels, respectively, with the latter shown as a circular statistic: 0-360°). Here, column number corresponds to the *i*th movement state i.e. column 1 = state 1 (directed/transit) etc.

**P6. MCMC summary plots for** $\sigma$ presented as in P3 excepting panel row number corresponds to the entry in the Σ process variance-covariance matrix where $\mathbf{I=}\left[ \begin{matrix} 1 & 0 \\ 0 & 1 \end{matrix} \right]$ (see Appendix S3 Table S3.1) i.e. the upper panel shows Σ*_lon_*, the lowest panel shows Σ*_lat_* and the middle panels show the identical (off diagonal) covariance.

**Reference.** Martyn Plummer (2015). rjags: Bayesian Graphical Models using MCMC. R

package version 3-15. <http://CRAN.R-project.org/package=rjags>.
