# Supplementary material for: Putting the behavior into animal movement modeling: Improved activity budgets from use of ancillary tag information
Source: Ecol Evol. 2016 Oct 20;6(22):8243–55. doi: 10.1002/ece3.2530 (PMC5108274; doi:10.1002/ece3.2530)
Supplement: Supplementary file 5 [file ECE3-6-8243-s005.pdf]

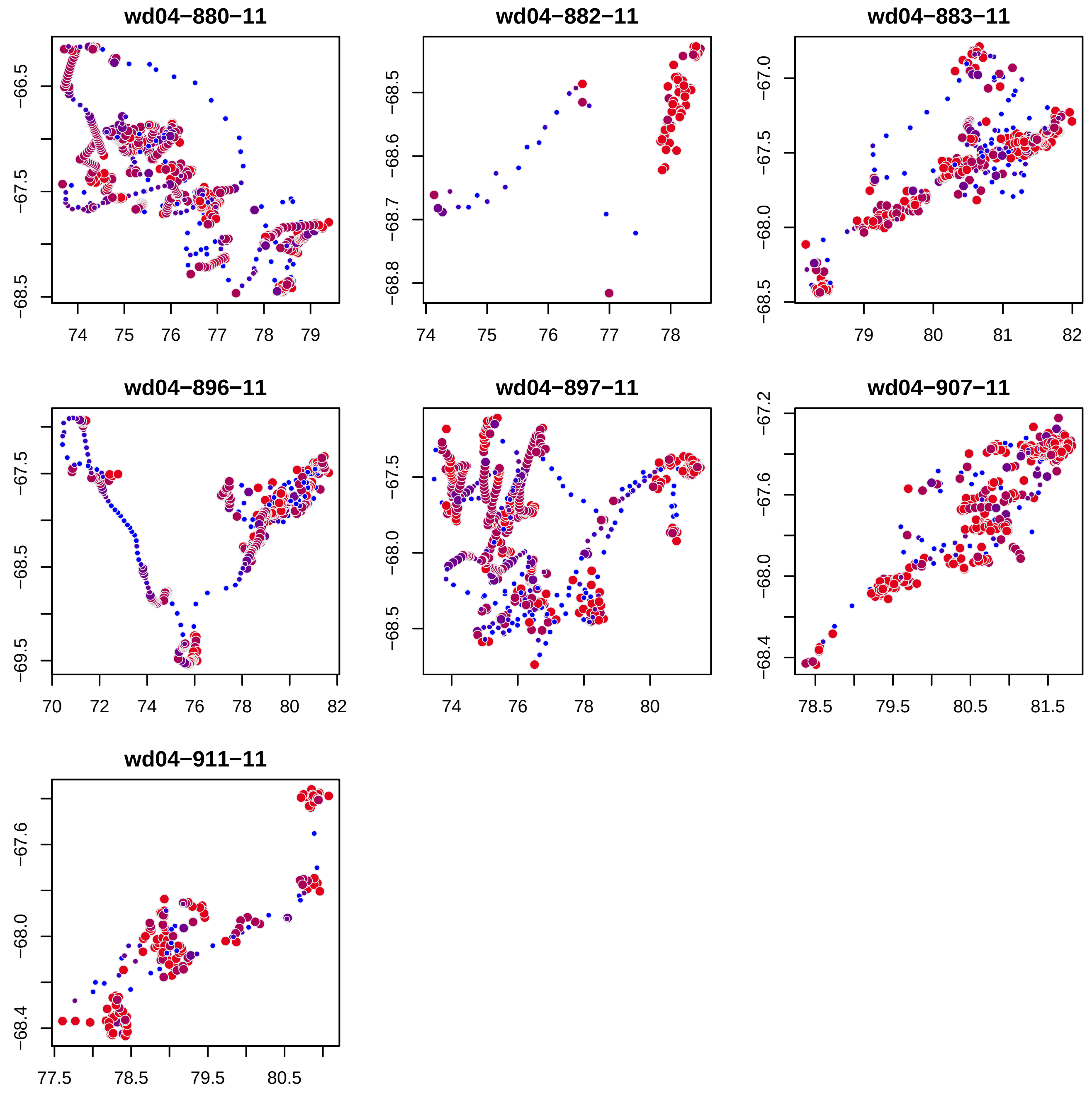
wd04−880−11
−66.5
−67.5
−68.5
74
75
76
77
78
79
wd04−882−11
−68.5
−68.6
−68.7
−68.8
74
75
76
77
78
wd04−883−11
−67.0
−67.5
−68.0
−68.5
79
80
81
82
wd04−896−11
−67.5
−68.5
−69.5
70
72
74
76
78
80
82
wd04−897−11
−67.5
−68.0
−68.5
74
76
78
80
wd04−907−11
−67.2
−67.6
−68.0
−68.4
78.5
79.5
80.5
81.5
wd04−911−11
−67.6
−68.0
−68.4
77.5
78.5
79.5
80.5

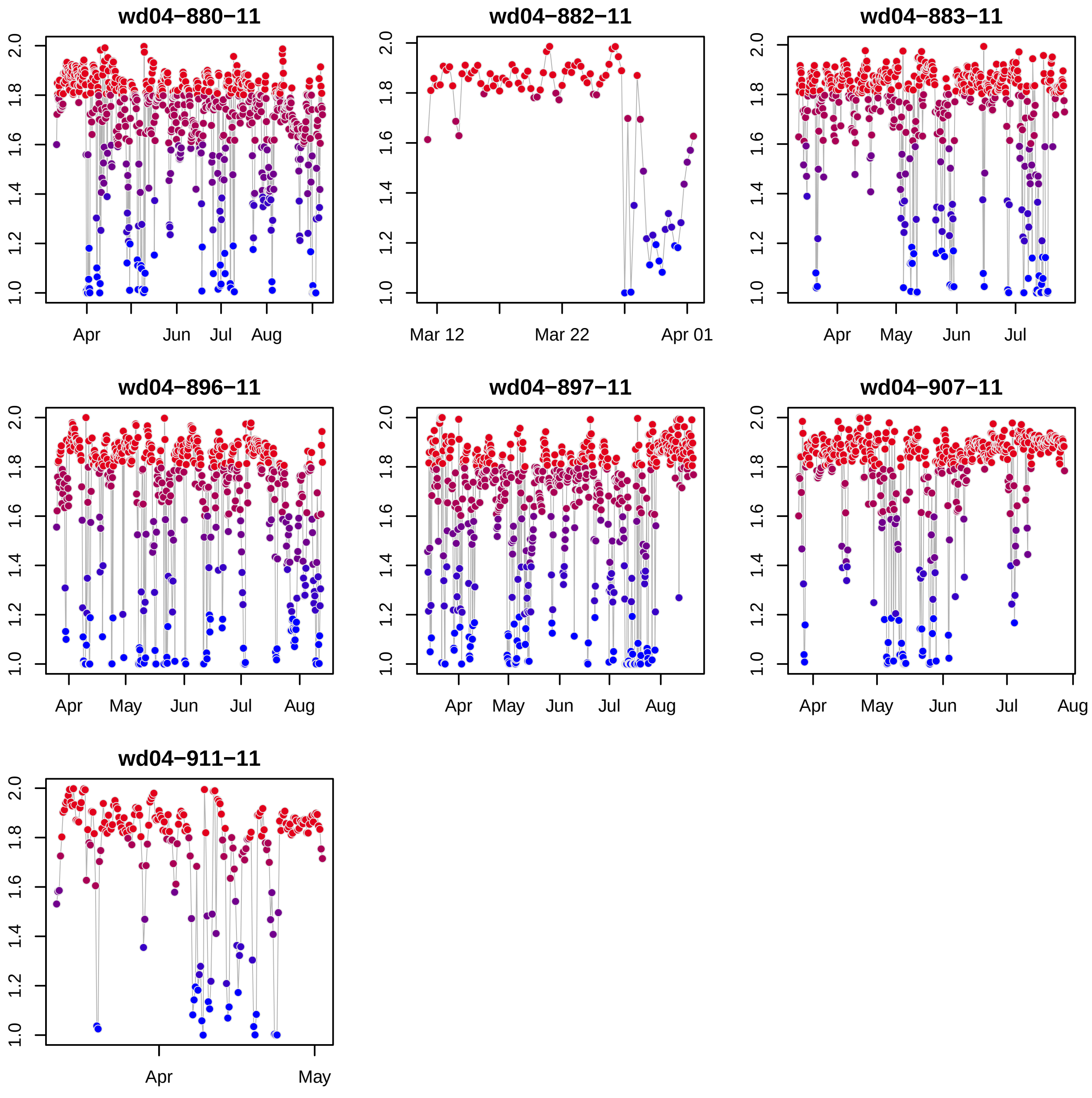
wd04−880−11
Apr
Jun
Jul
Aug
wd04−882−11
Mar 12
Mar 22
Apr 01
wd04−883−11
Apr
May
Jun
Jul
wd04−896−11
Apr
May
Jun
Jul
Aug
wd04−897−11
Apr
May
Jun
Jul
Aug
wd04−907−11
Apr
May
Jun
Jul
Aug
wd04−911−11
Apr
May
1.0
1.2
1.4
1.6
1.8
2.0

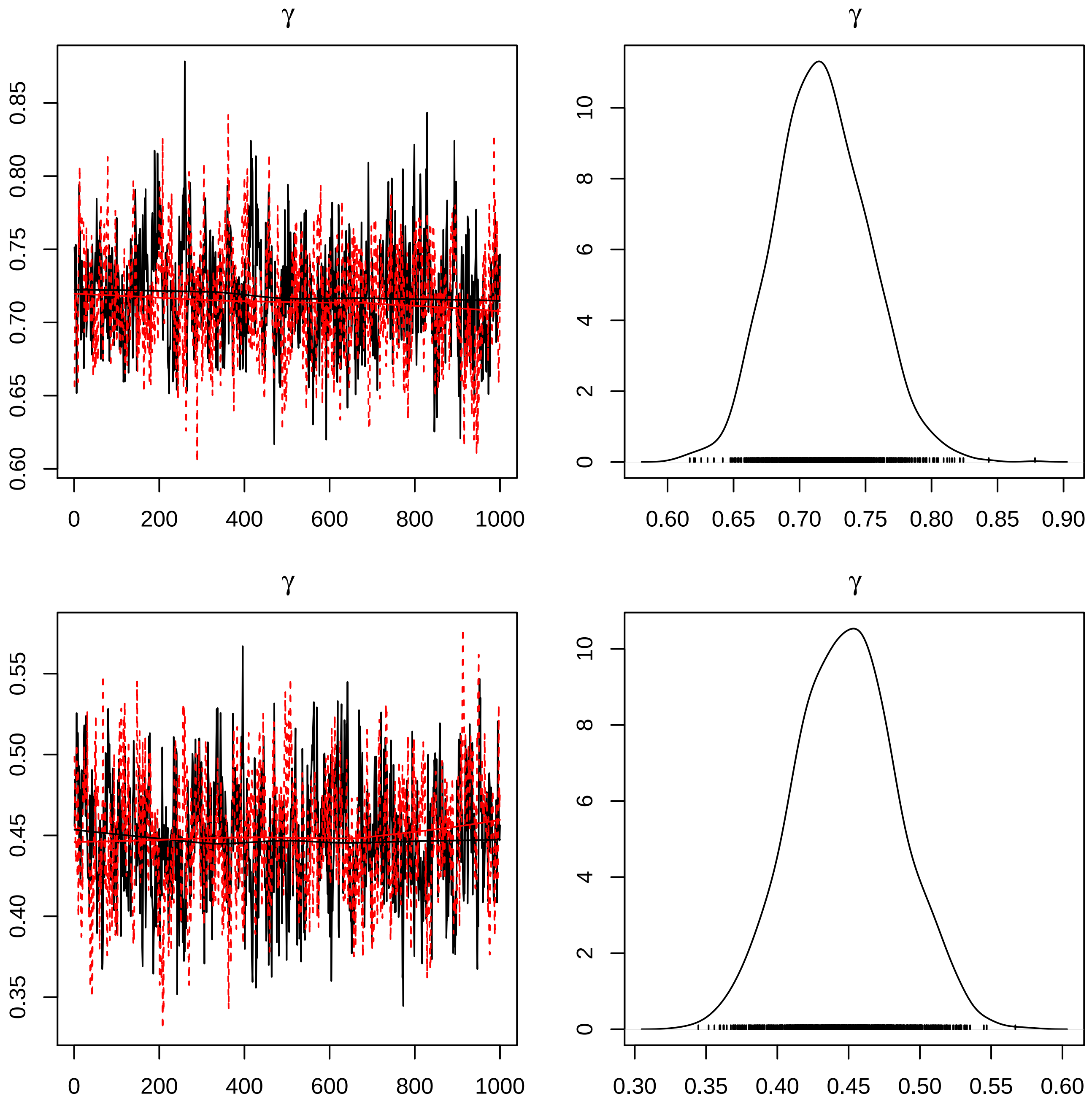
γ
0.60
0.65
0.70
0.75
0.80
0.85
0
200
400
600
800
1000
γ
0
2
4
6
8
10
0.60
0.65
0.70
0.75
0.80
0.85
0.90
γ
0.35
0.40
0.45
0.50
0.55
0
200
400
600
800
1000
γ
0
2
4
6
8
10
0.30
0.35
0.40
0.45
0.50
0.55
0.60

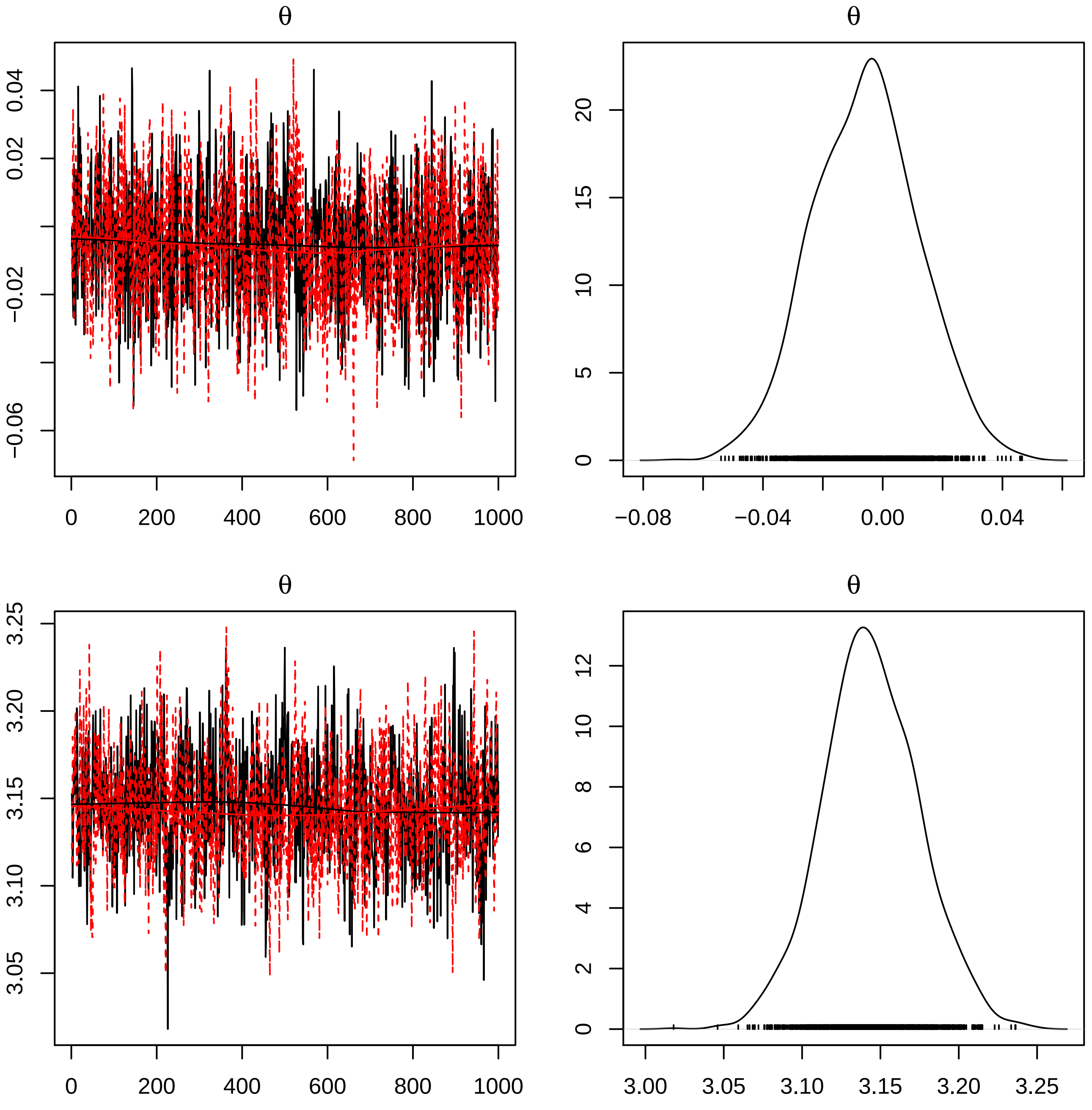
θ
0.04
0.02
−0.02
−0.06
0
200
400
600
800
1000
θ
20
15
10
5
0
−0.08
−0.04
0.00
0.04
θ
3.25
3.20
3.15
3.10
3.05
0
200
400
600
800
1000
θ
12
10
8
6
4
2
0
3.00
3.05
3.10
3.15
3.20
3.25

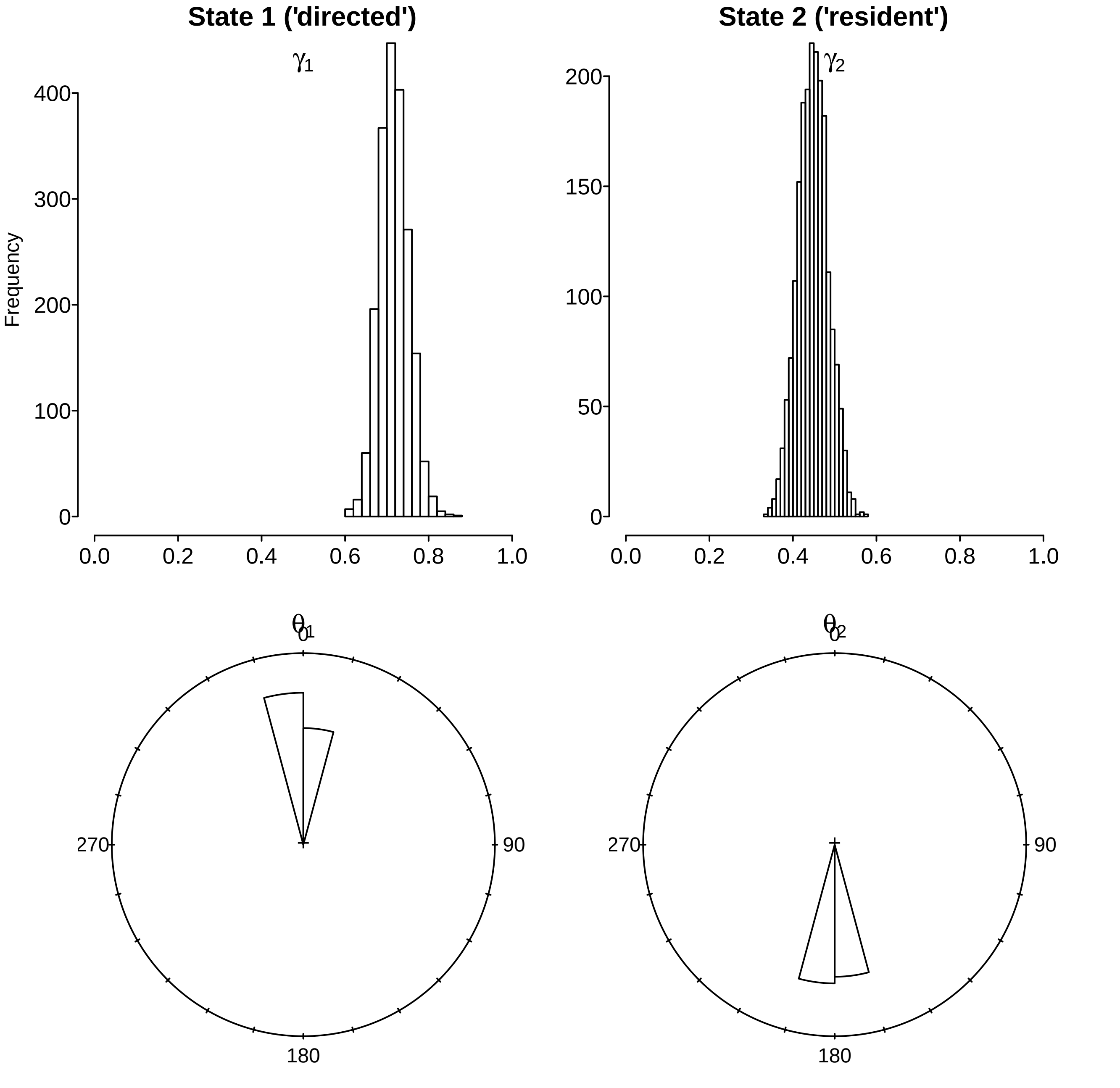

State 1 ('directed')
State 2 ('resident')
Frequency
γ_1
γ_2
0
100
200
300
400
0
50
100
150
200
0.0
0.2
0.4
0.6
0.8
1.0
θ_1
θ_2
0
90
180
270

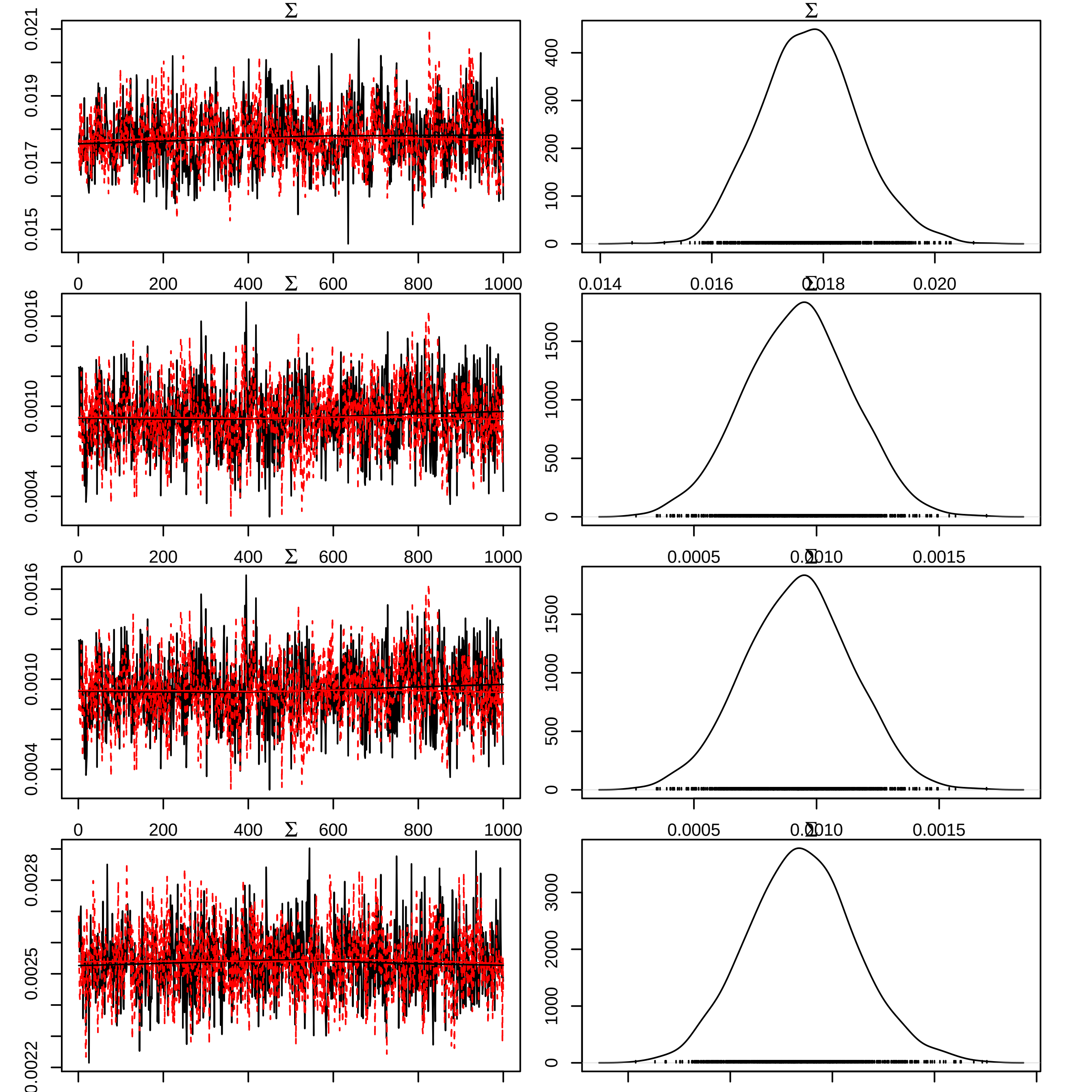
Σ
0.015
0.017
0.019
0.021
0
200
400
600
800
1000
0
100
200
300
400
0.014
0.016
0.018
0.020
0.0004
0.0010
0.0016
0
500
1000
1500
0.0005
0.0010
0.0015
0.0022
0.0025
0.0028
0
1000
2000
3000
