# Supplementary figures and images for: Putting the behavior into animal movement modeling: Improved activity budgets from use of ancillary tag information
Source: Ecol Evol. 2016 Oct 20;6(22):8243–55. doi: 10.1002/ece3.2530 (PMC5108274; doi:10.1002/ece3.2530)

**wd04-880-11**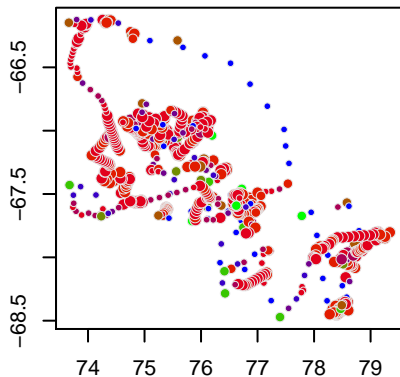**wd04-882-11**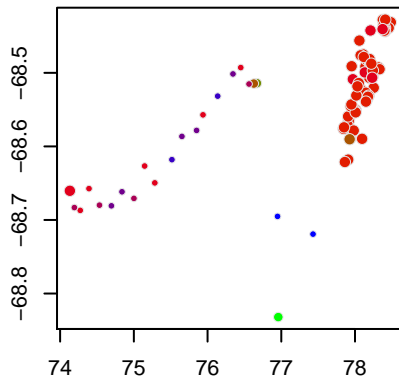**wd04-883-11**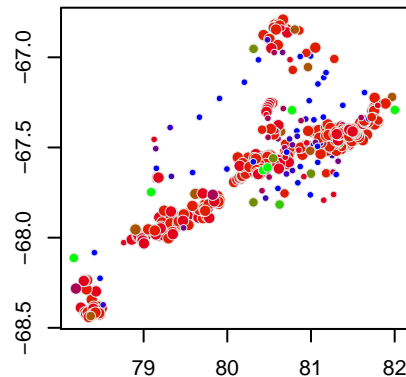**wd04-896-11**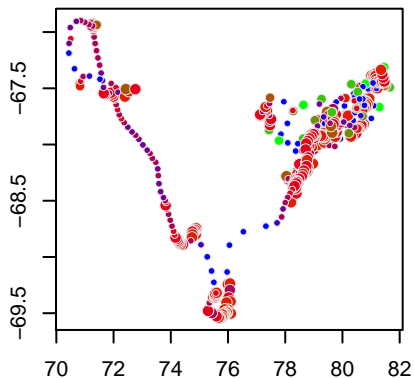**wd04-897-11**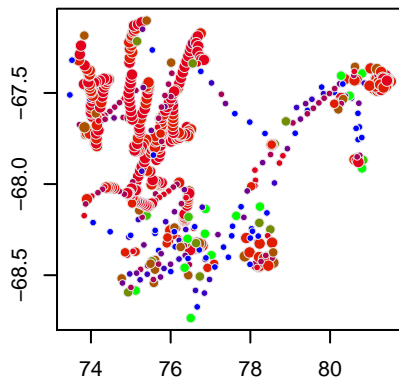**wd04-907-11**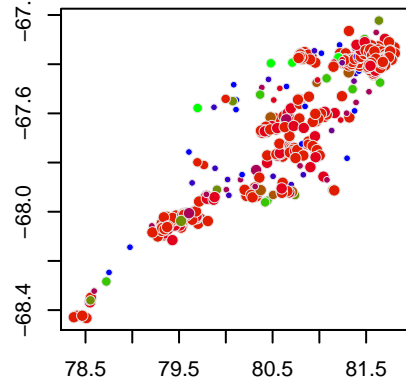**wd04-911-11**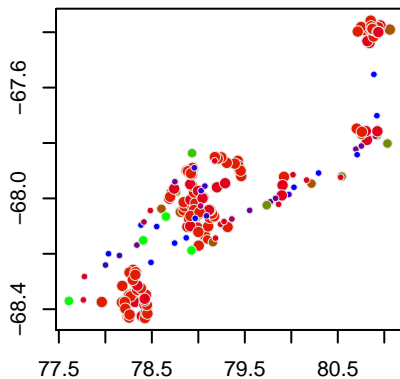

**wd04-880-11**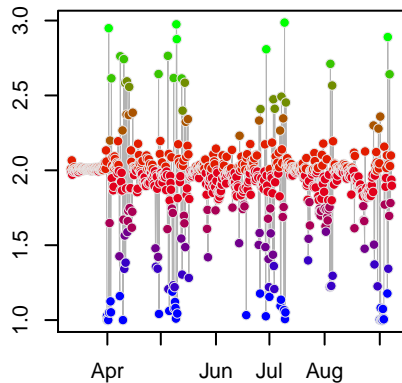**wd04-882-11**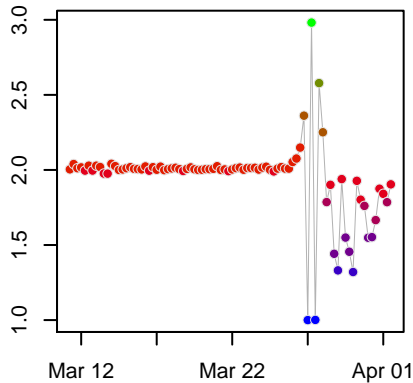**wd04-883-11**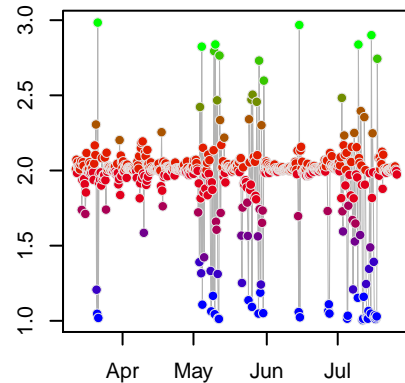**wd04-896-11**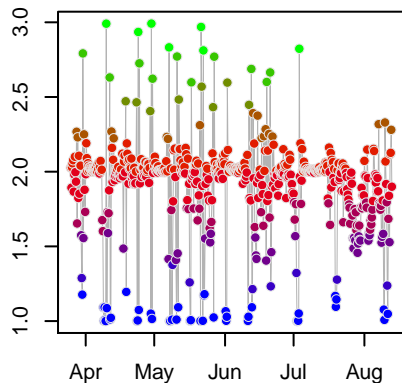**wd04-897-11**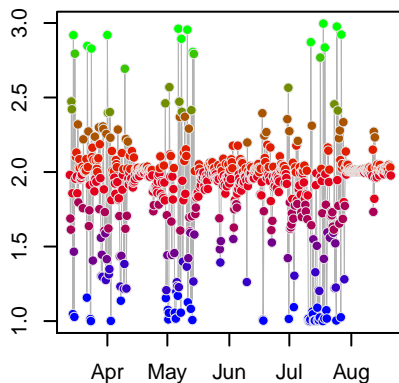**wd04-907-11**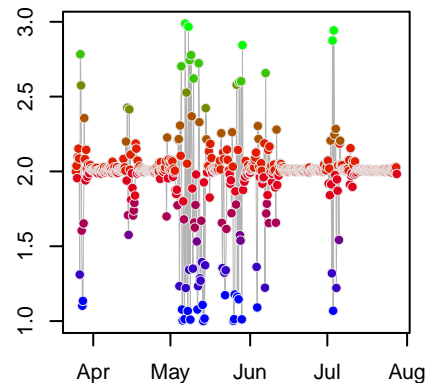**wd04-911-11**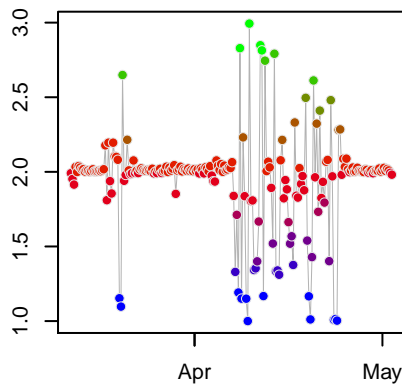

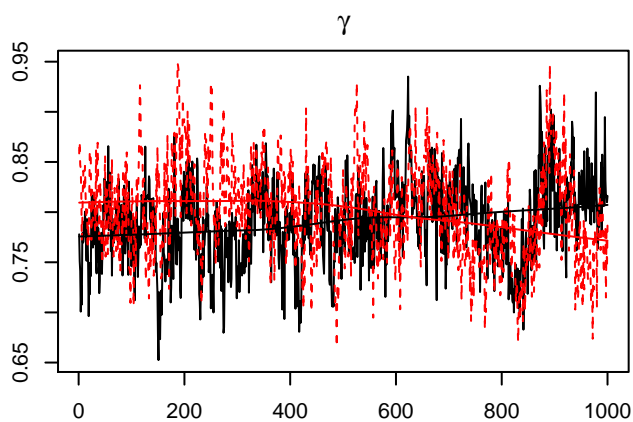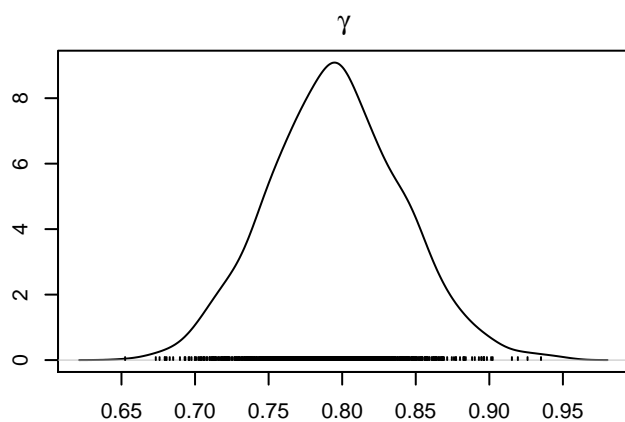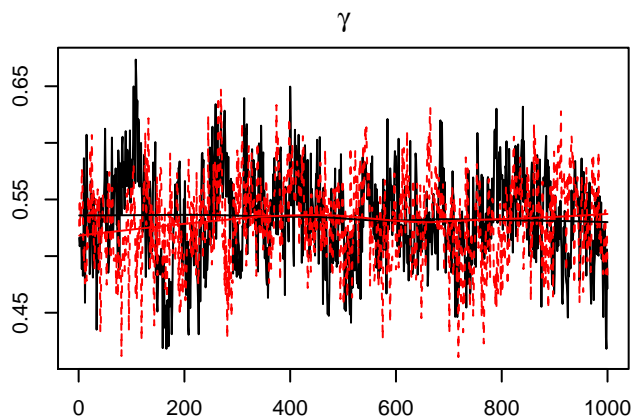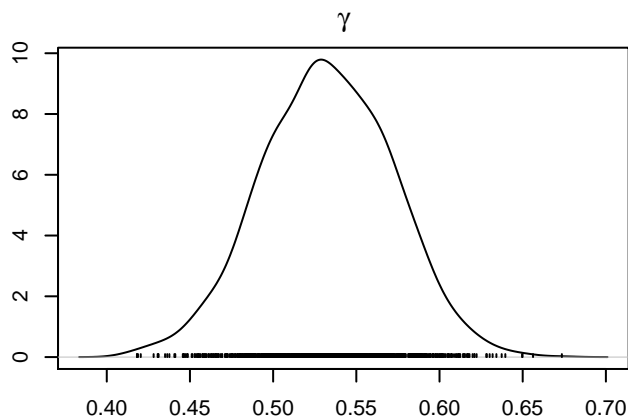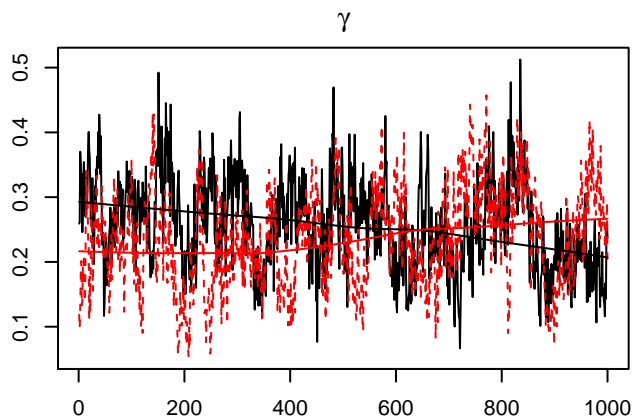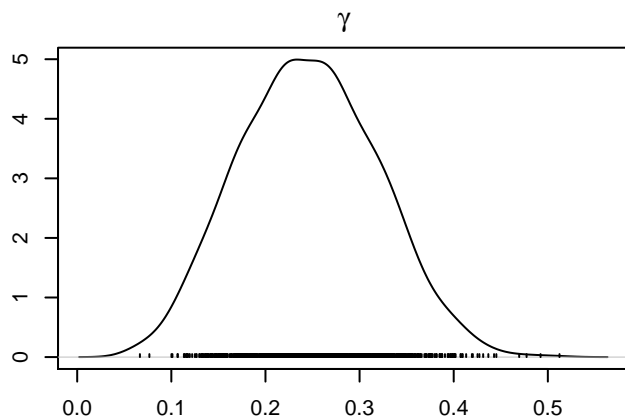

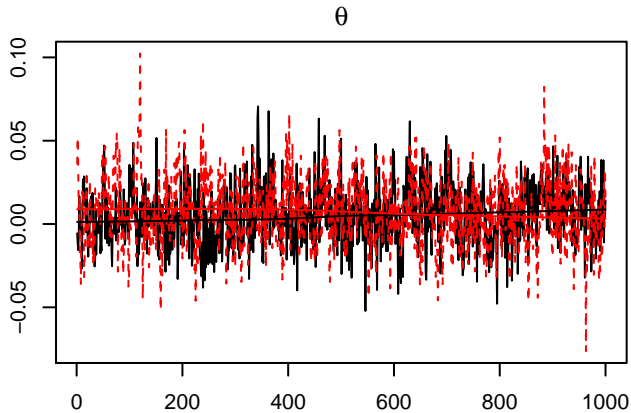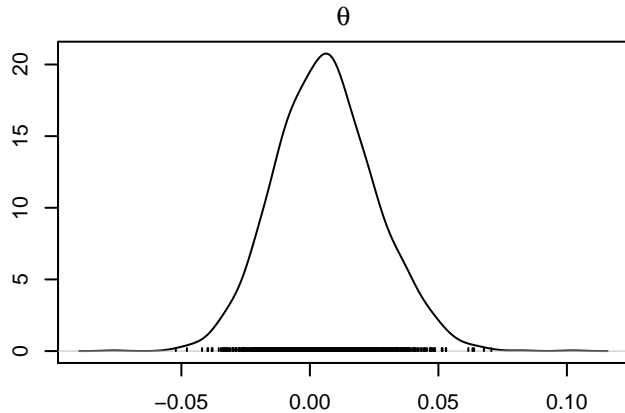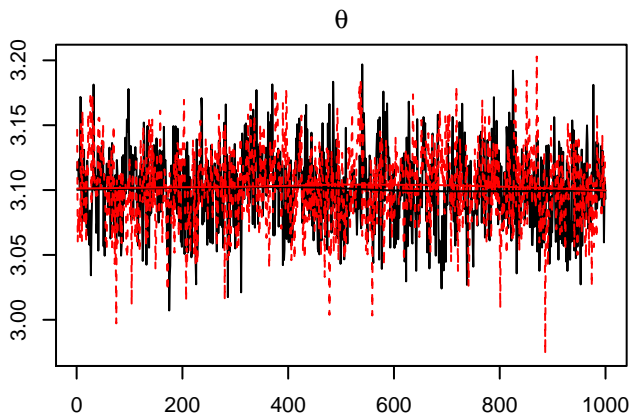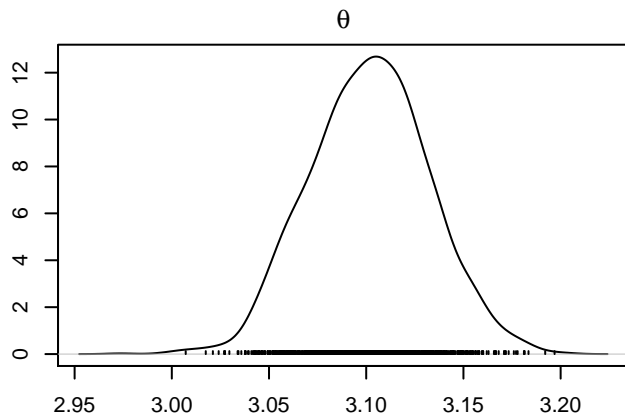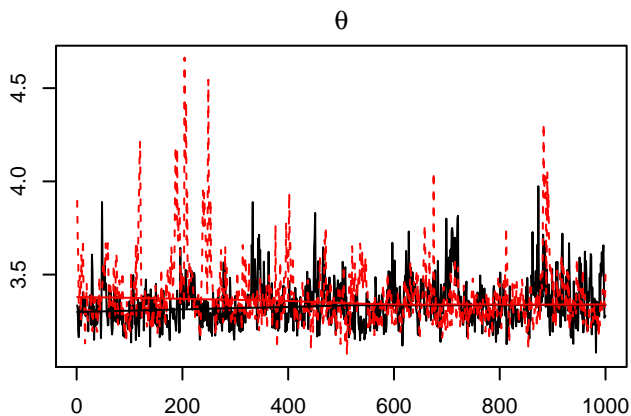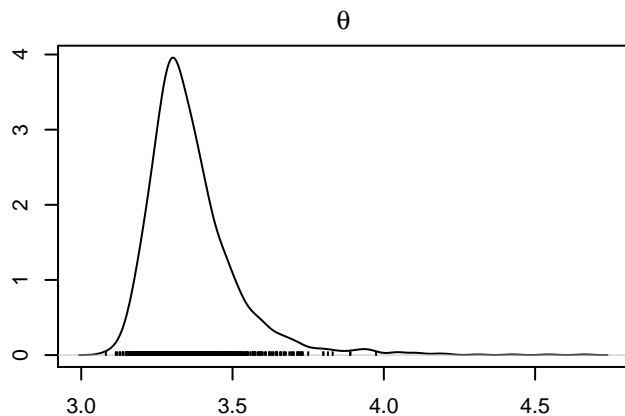

**State 1 ('directed')**

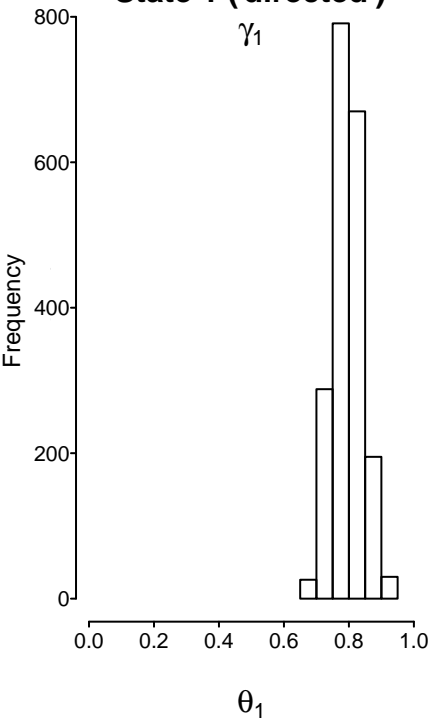

**State 2 ('resident')**

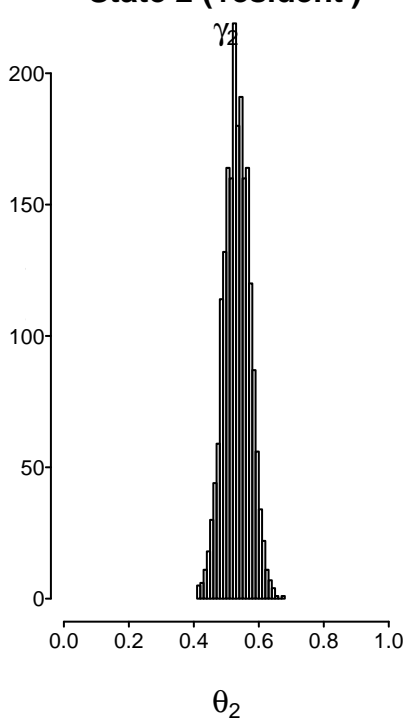

**State 3 ('unaugmented')**

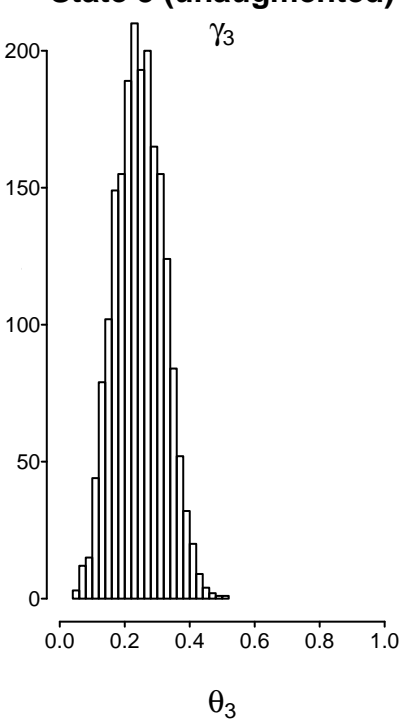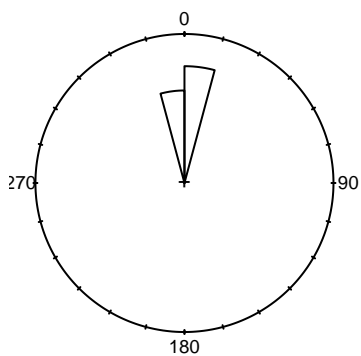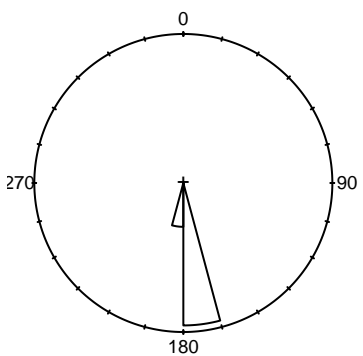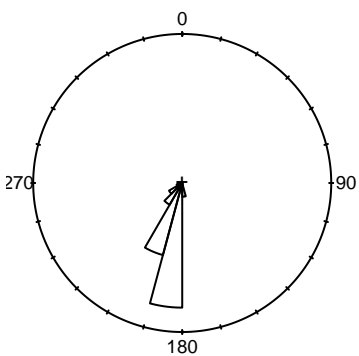

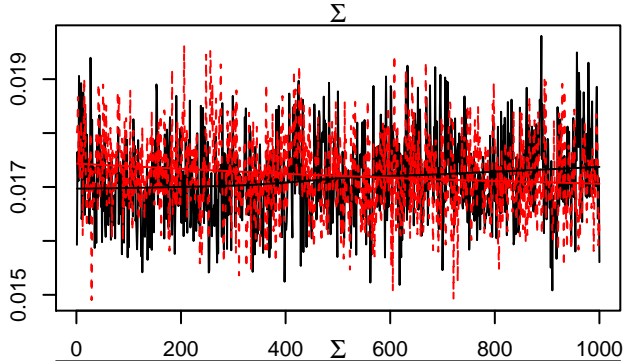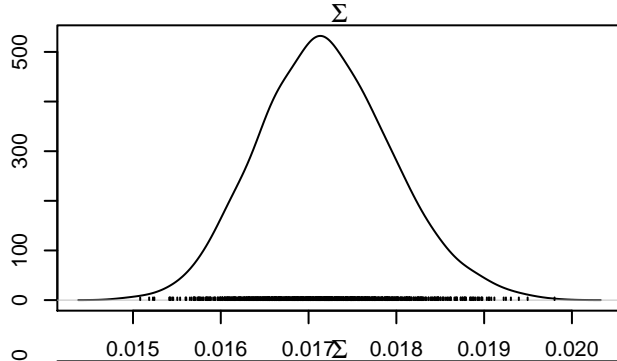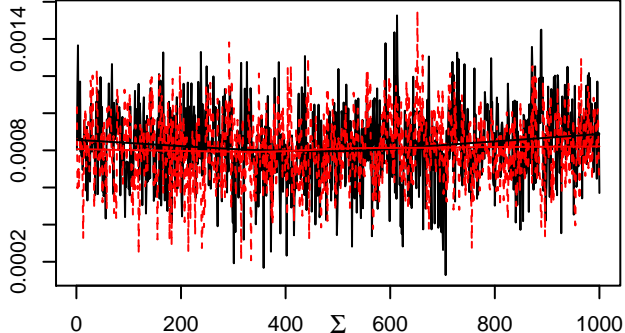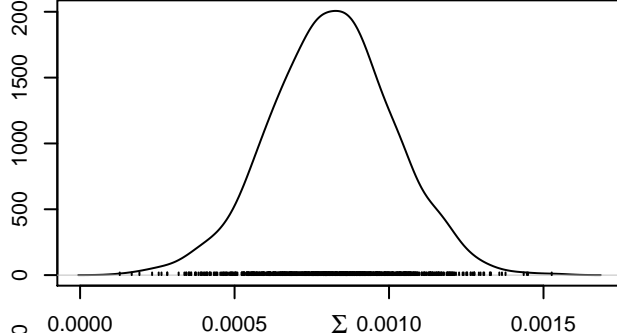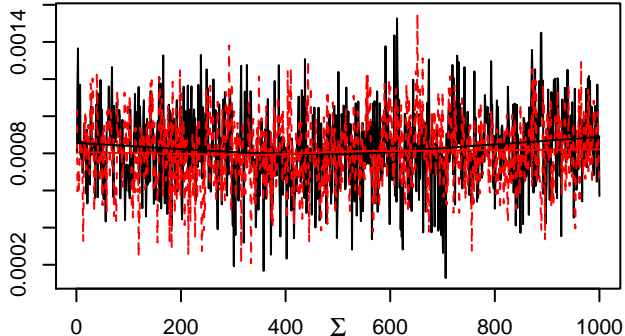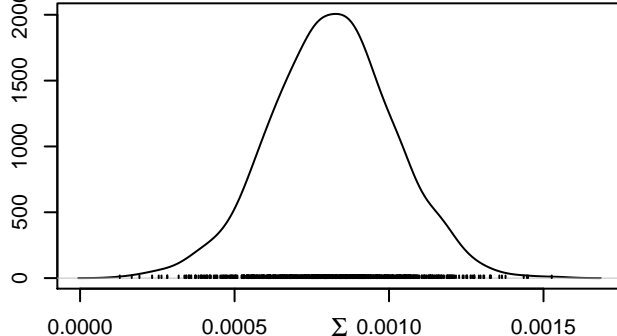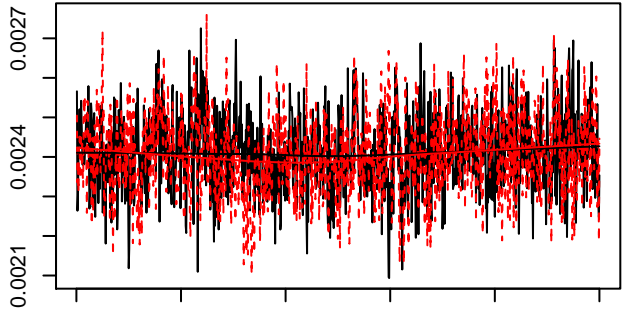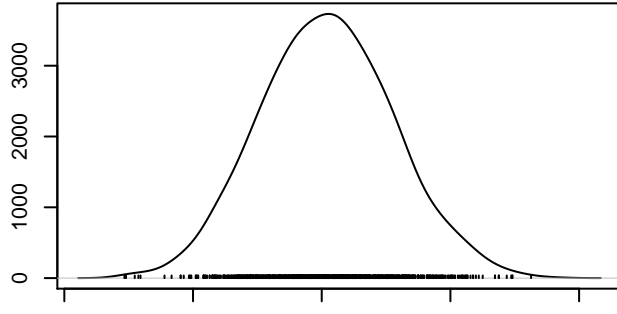

Supplement: Supplementary file 6 [file ECE3-6-8243-s006.pdf]

**wd04-880-11**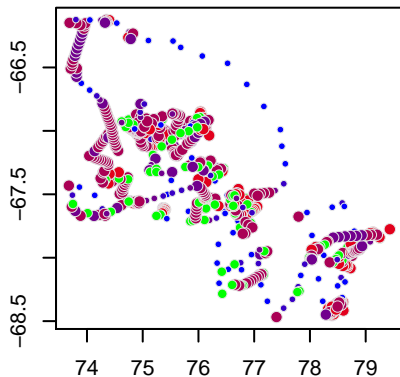**wd04-882-11**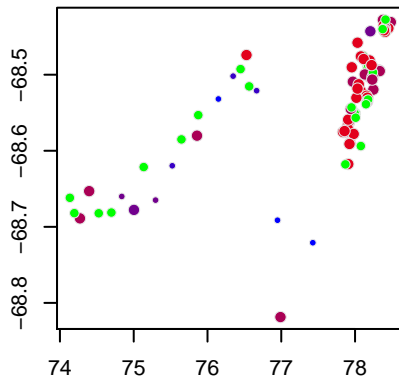**wd04-883-11**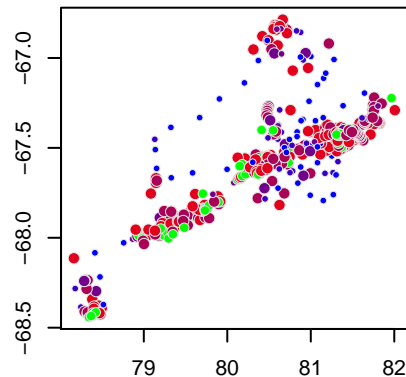**wd04-896-11**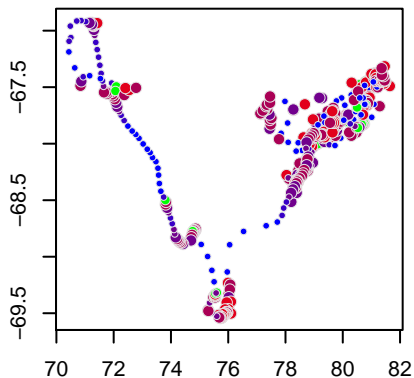**wd04-897-11**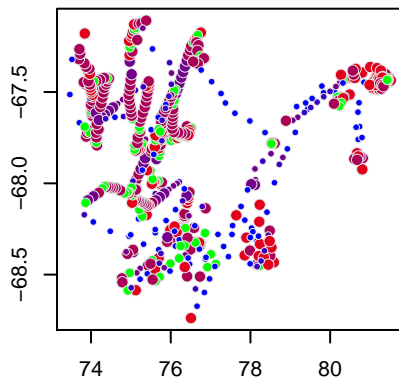**wd04-907-11**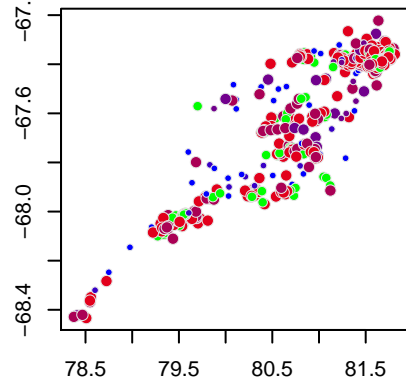**wd04-911-11**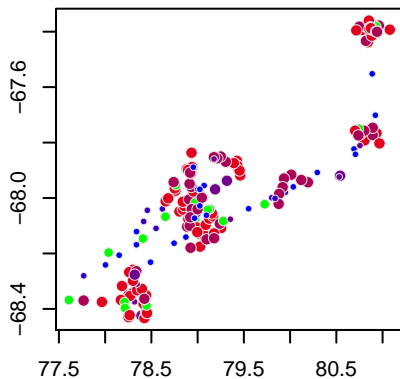

**wd04-880-11**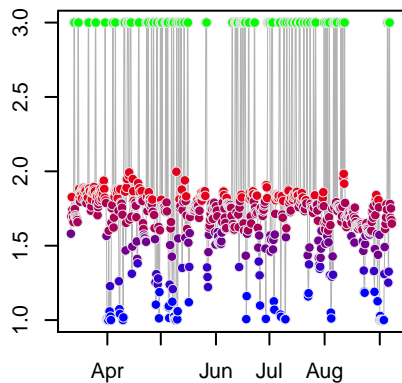**wd04-882-11**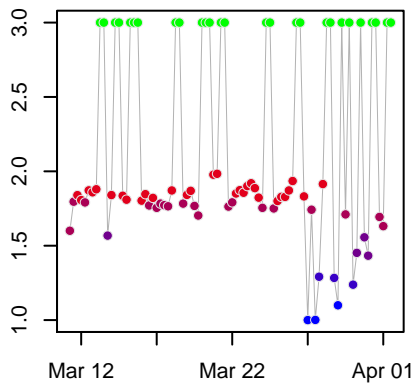**wd04-883-11**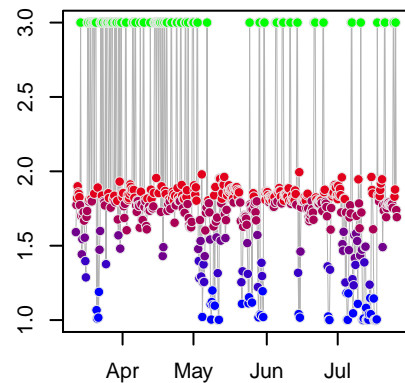**wd04-896-11**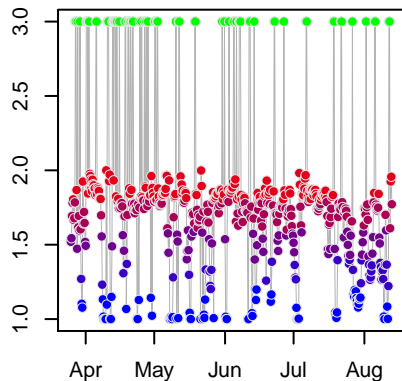**wd04-897-11**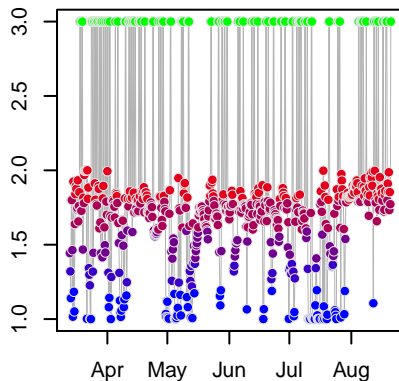**wd04-907-11**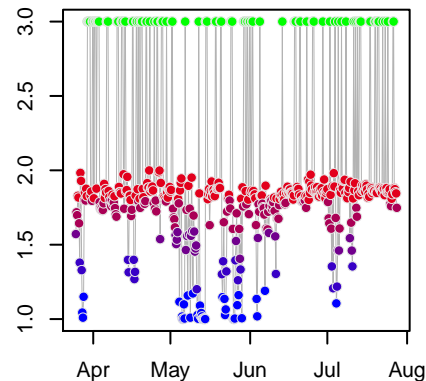**wd04-911-11**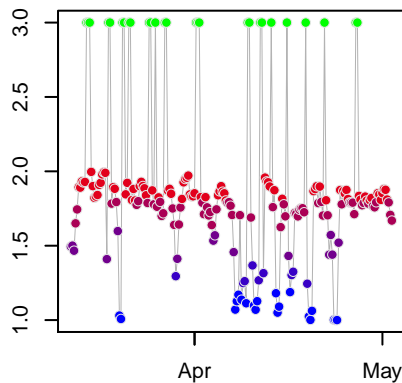

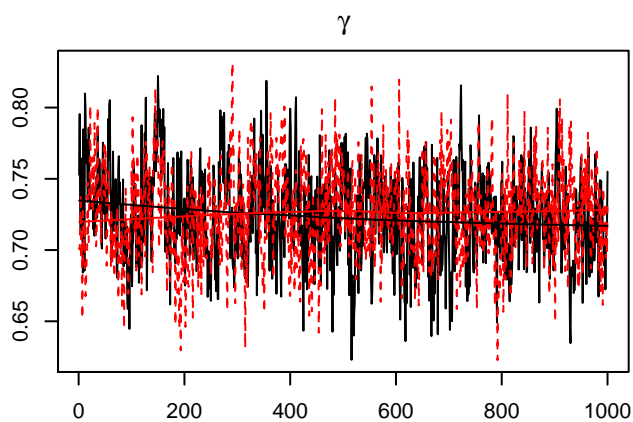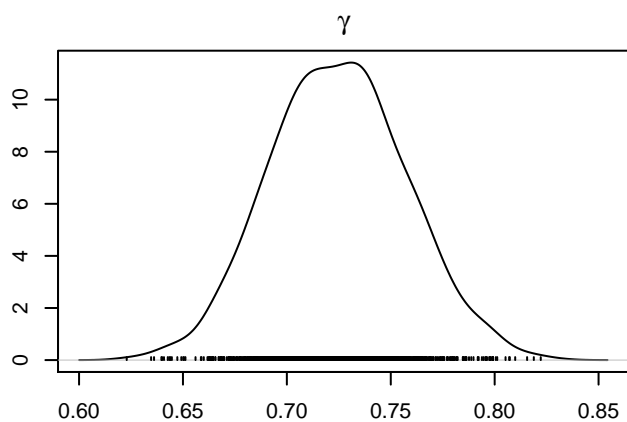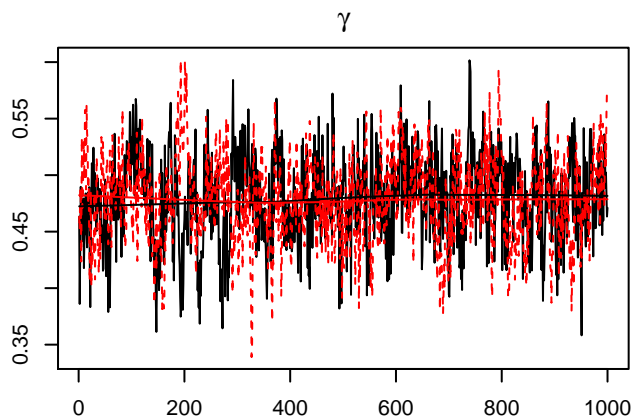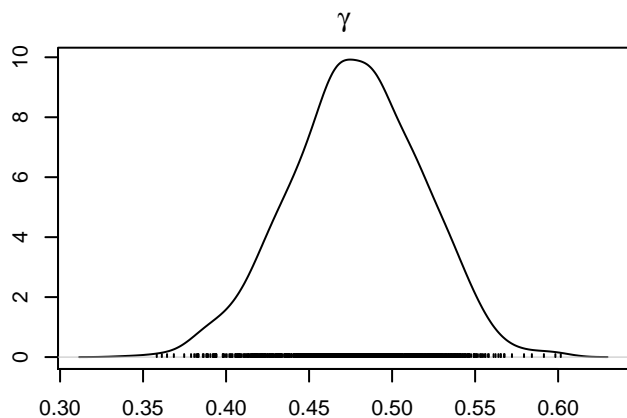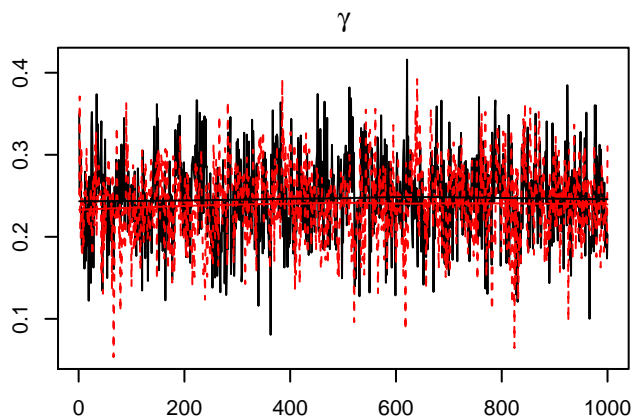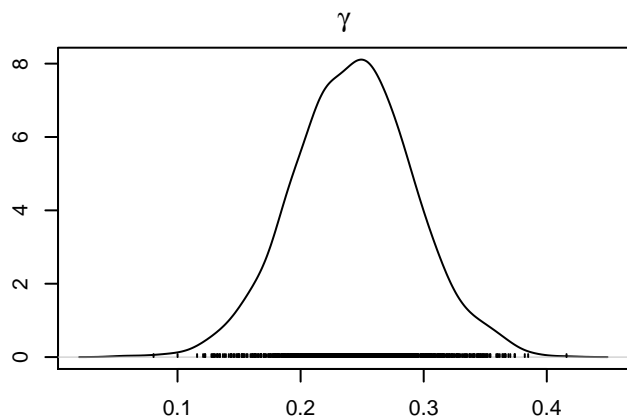

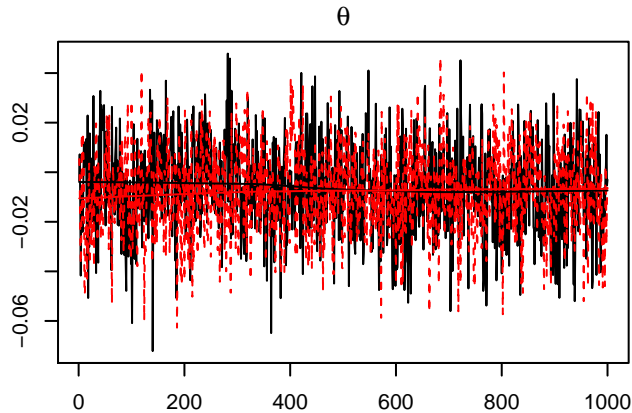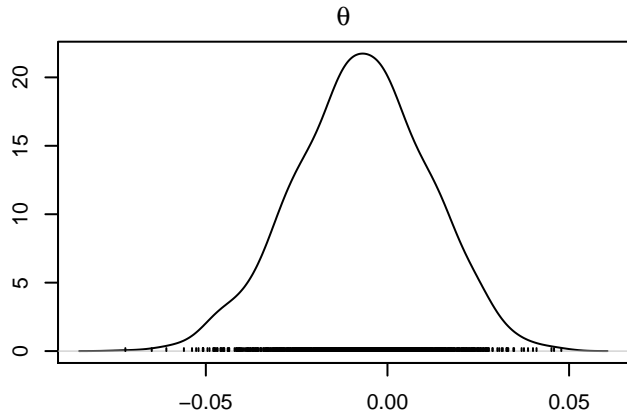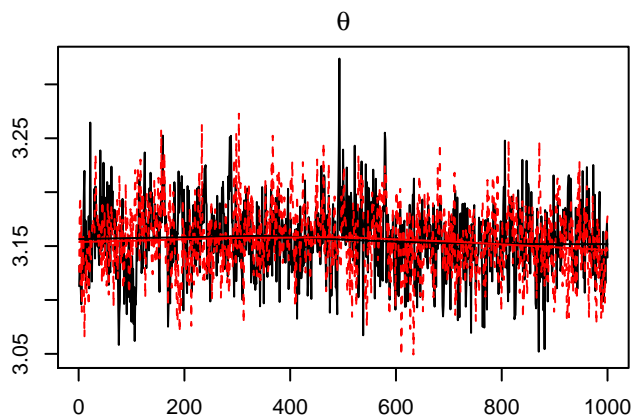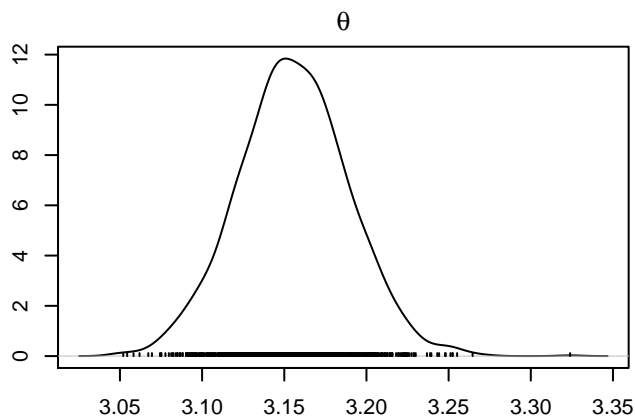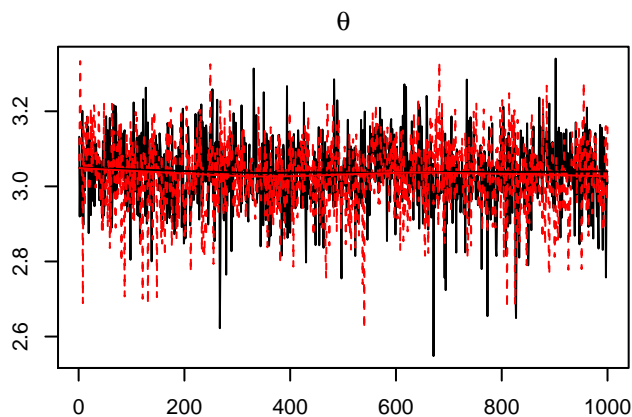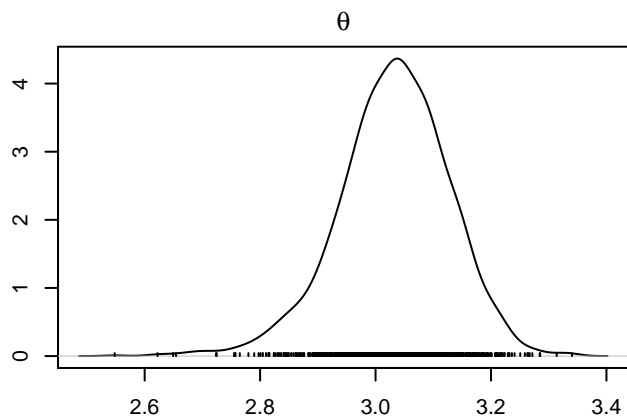

**State 1 ('directed')**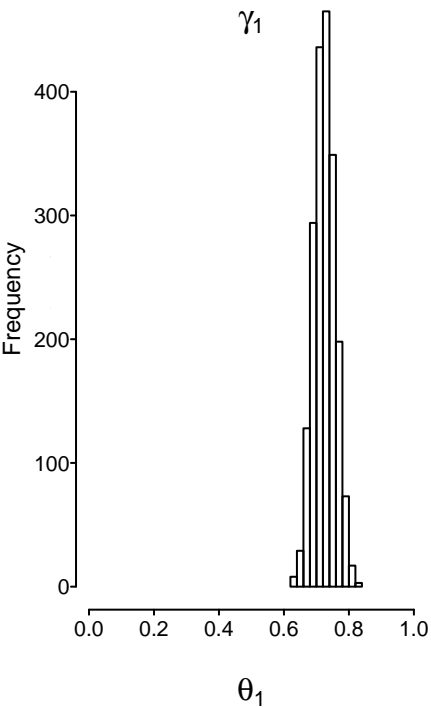**State 2 ('resident')**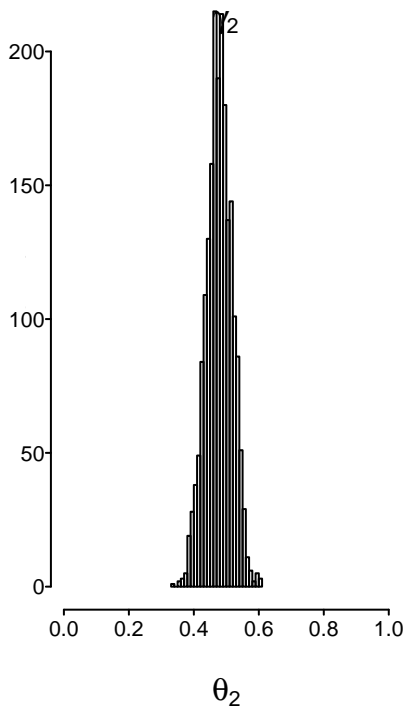**State 3 ('haulout')**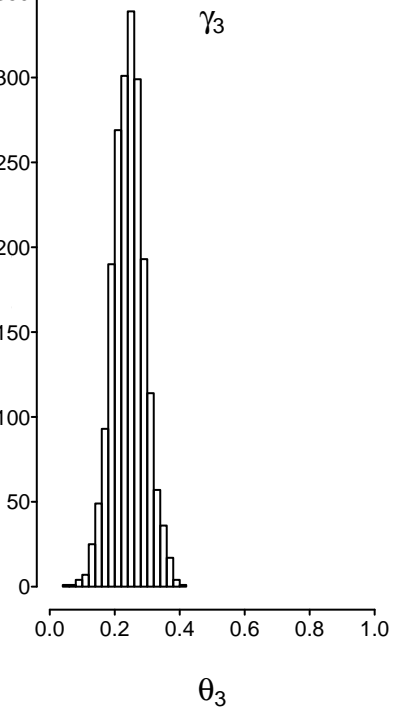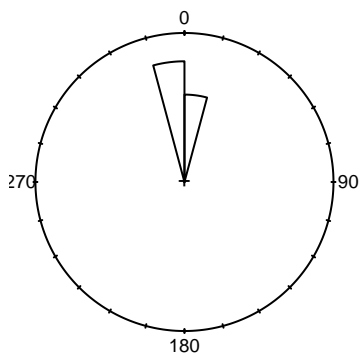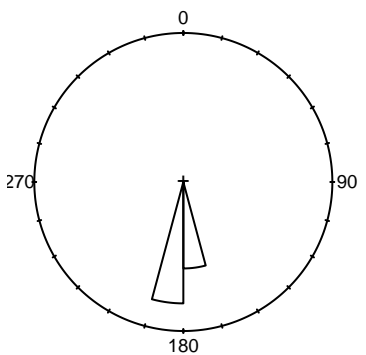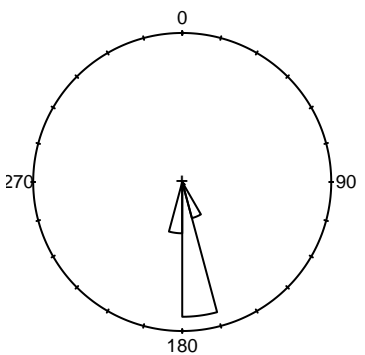

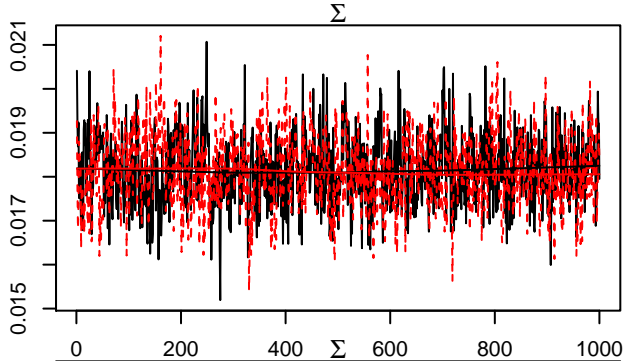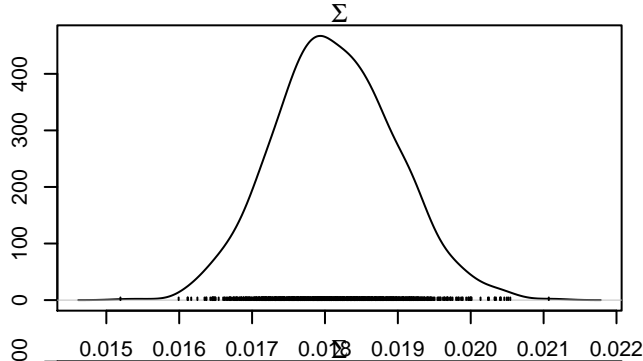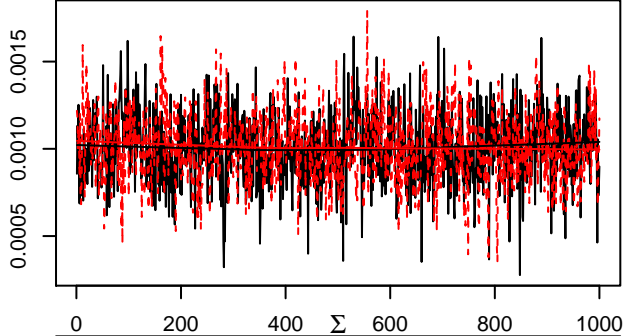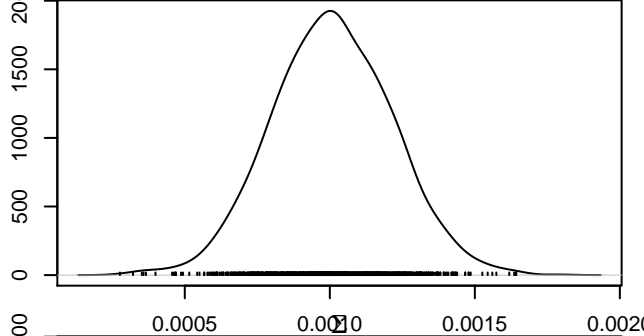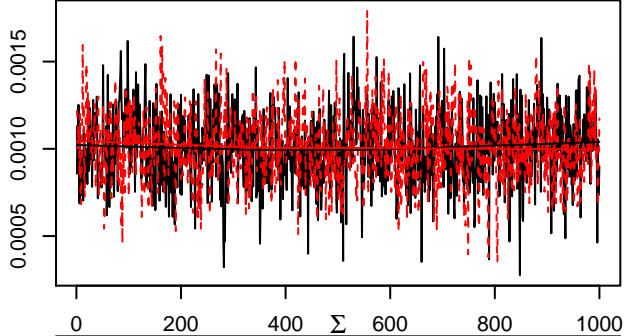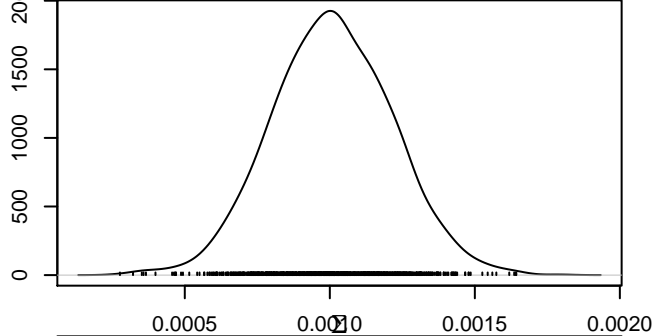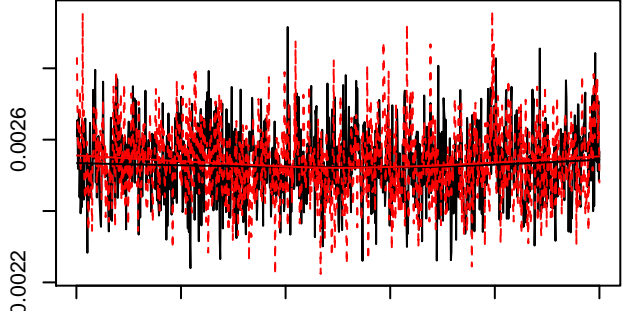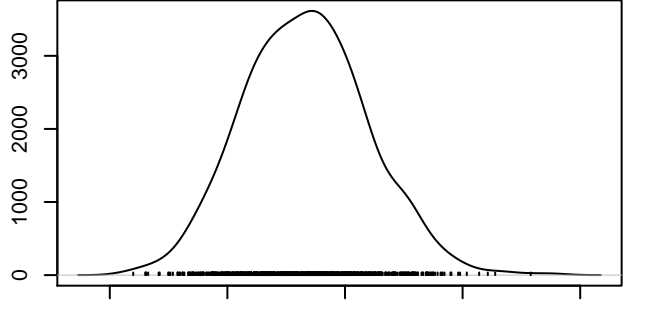

Supplement: Supplementary file 7 [file ECE3-6-8243-s007.pdf]

**FM07-S**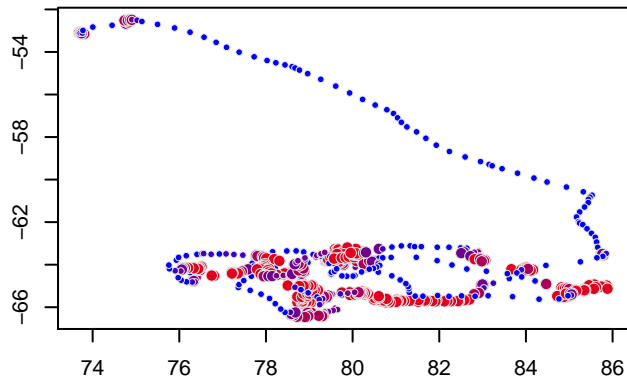**FM10-S**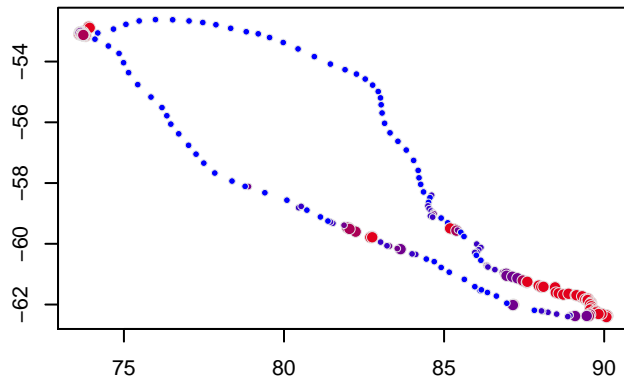**FM11-S**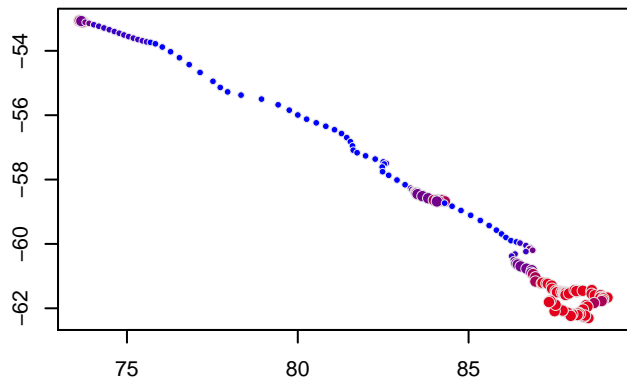**FM17-S**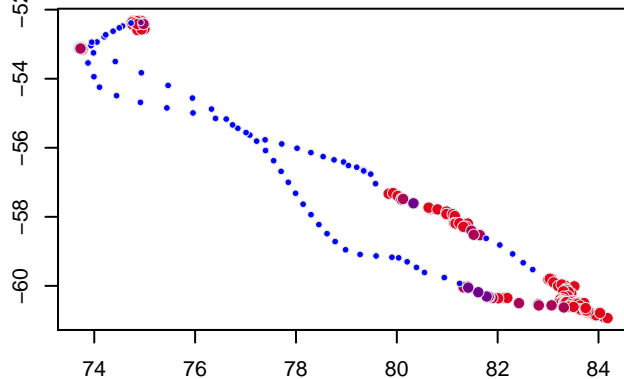**FM19-S**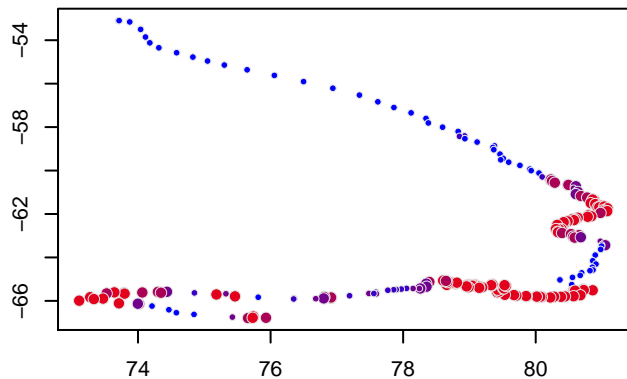

**FM07-S**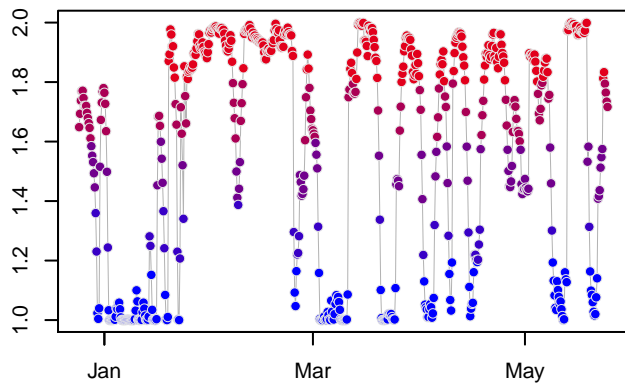**FM10-S**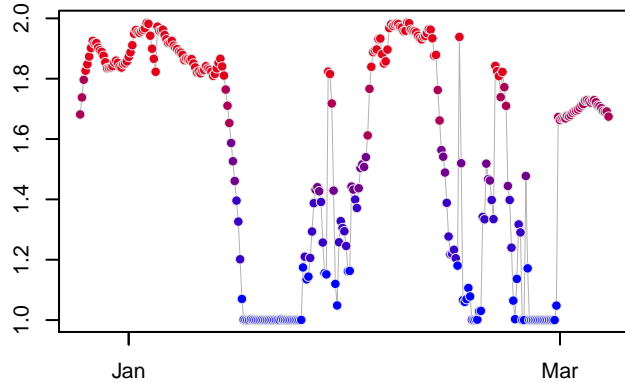**FM11-S**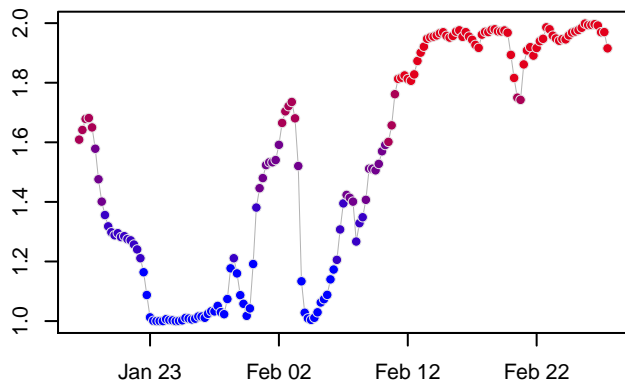**FM17-S**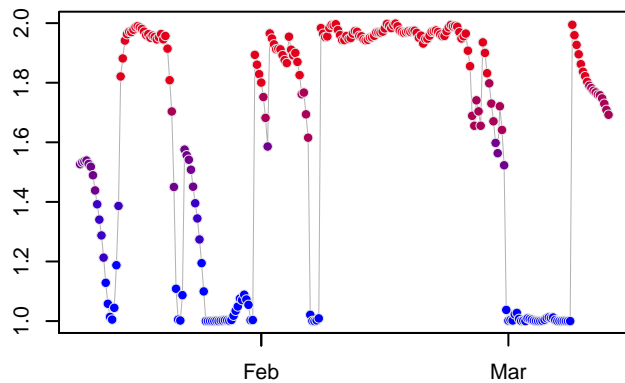**FM19-S**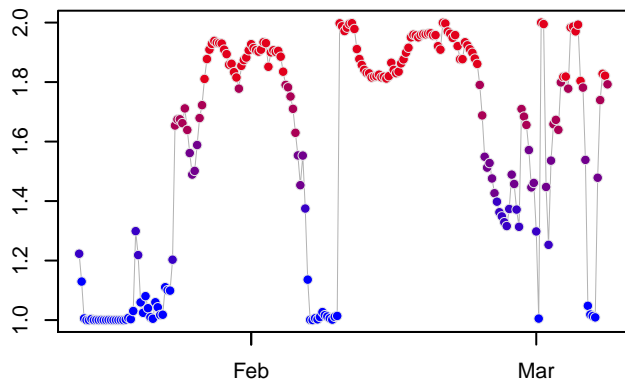

$\gamma$ 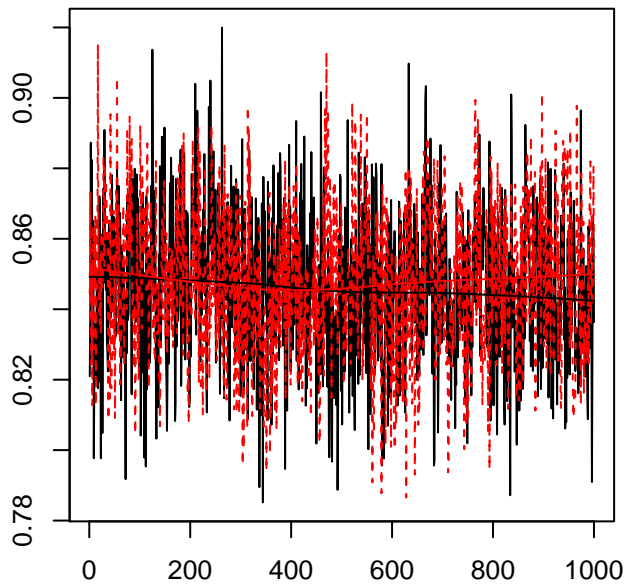 $\gamma$ 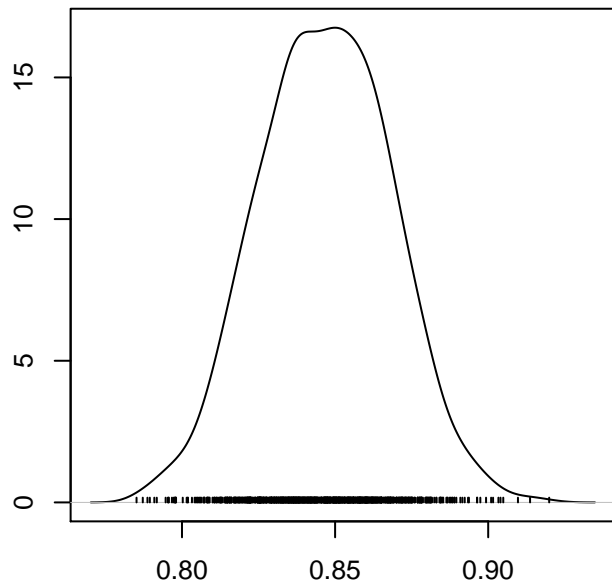 $\gamma$ 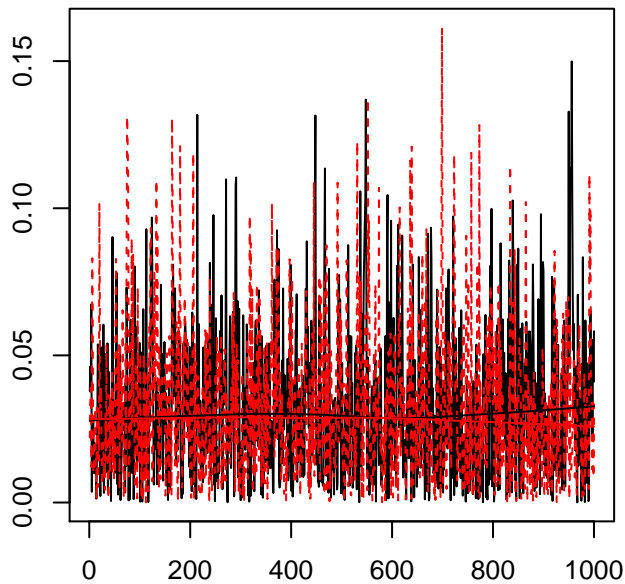 $\gamma$ 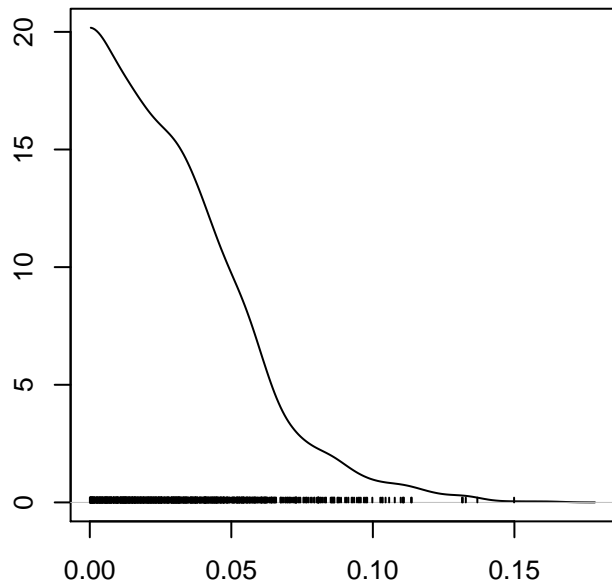

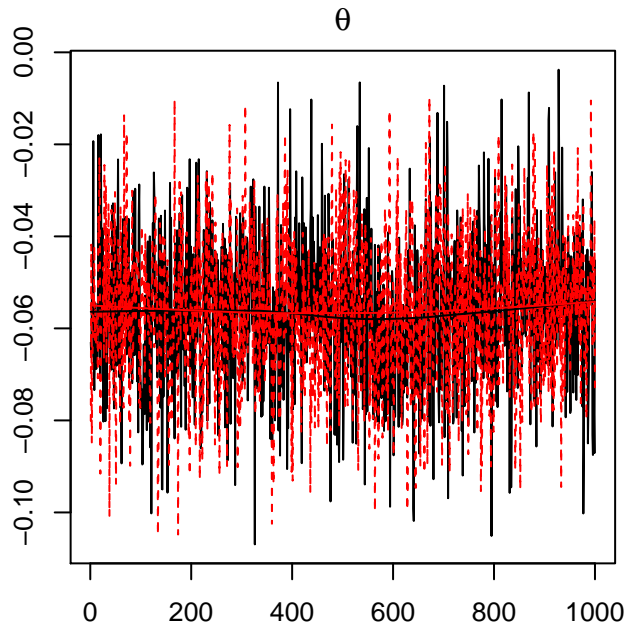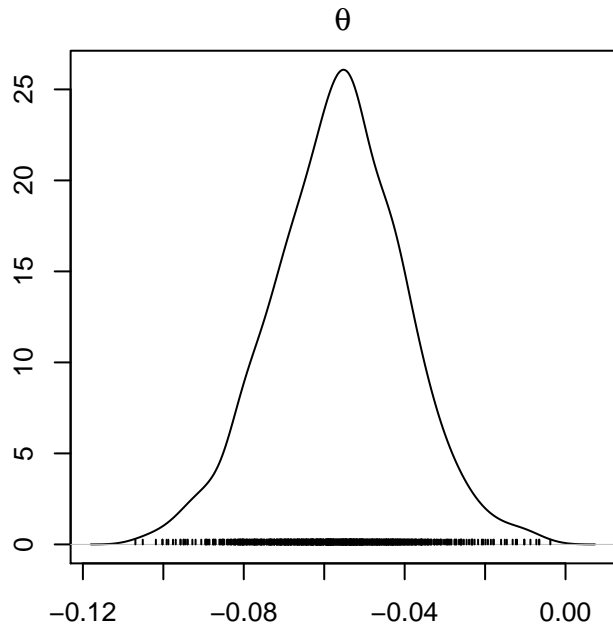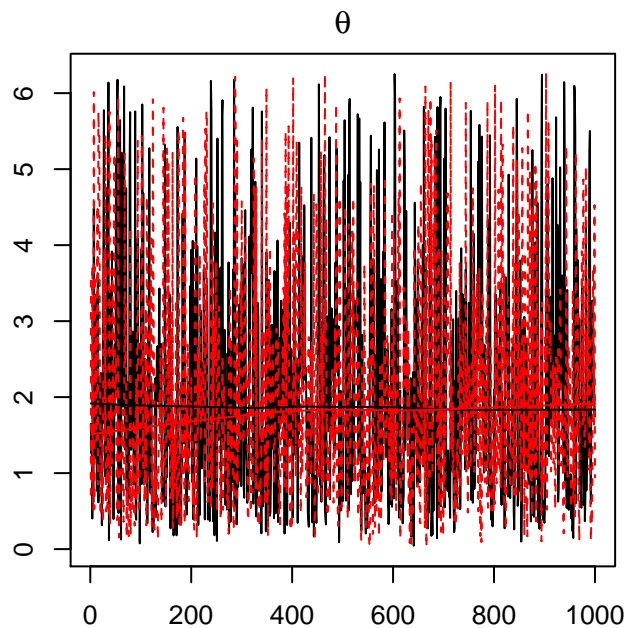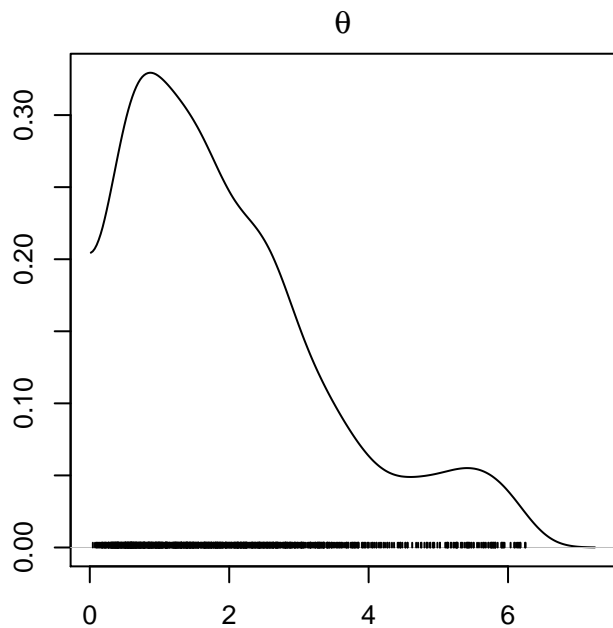

**State 1 ('directed')**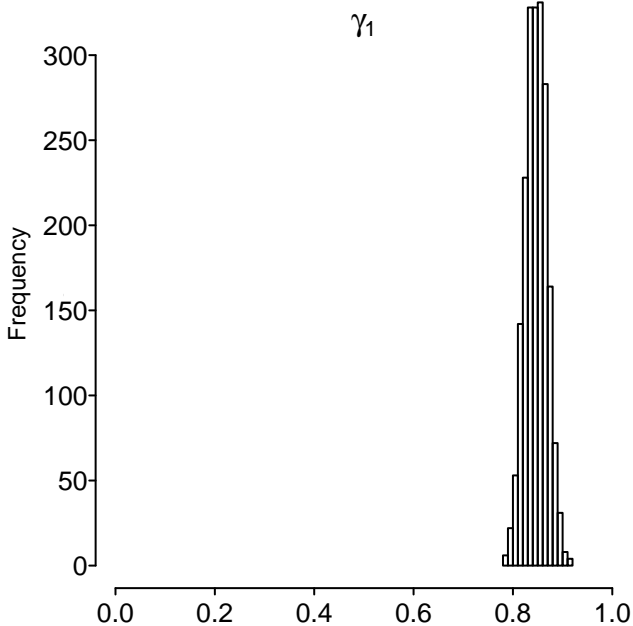**State 2 ('resident')**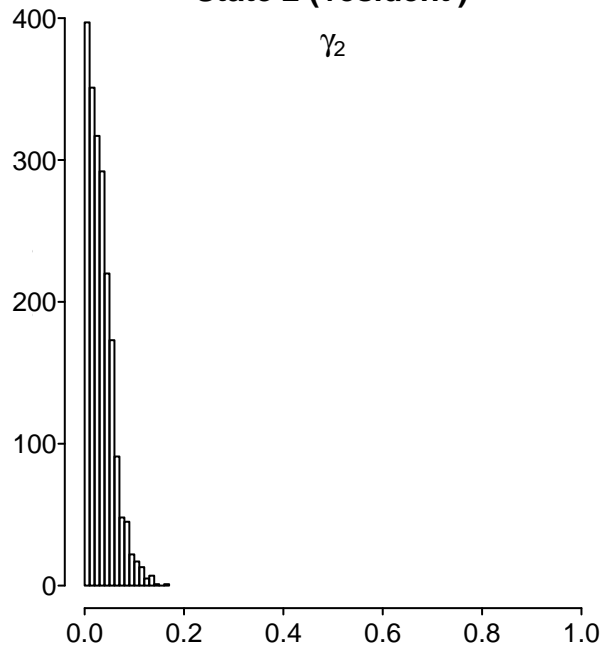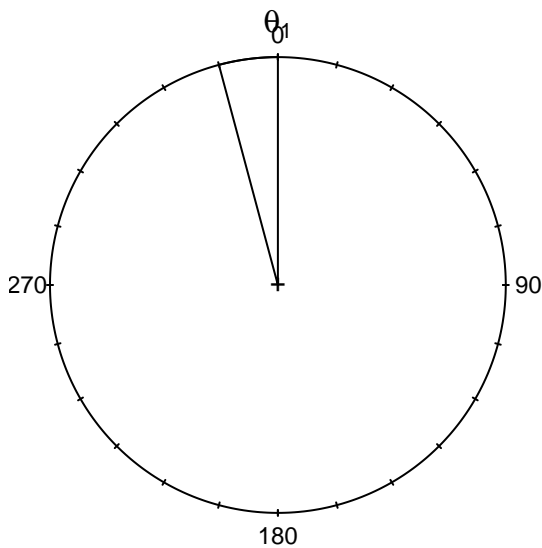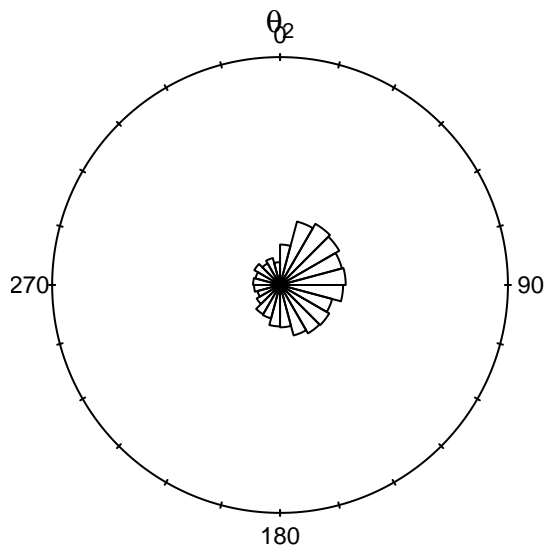

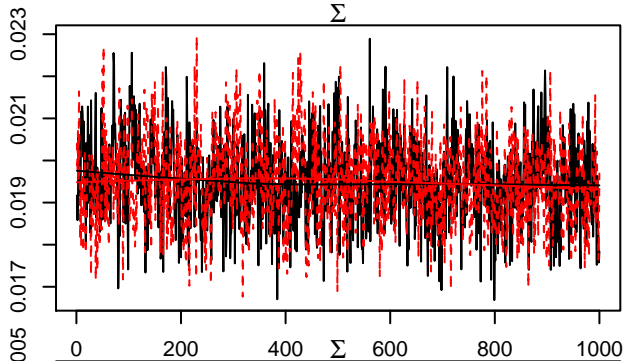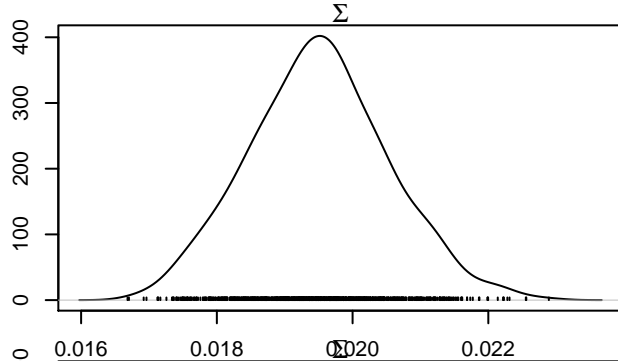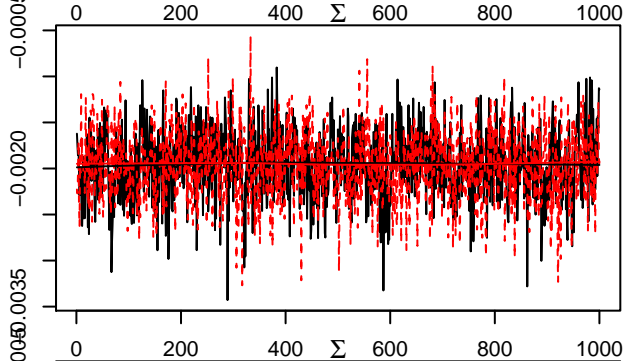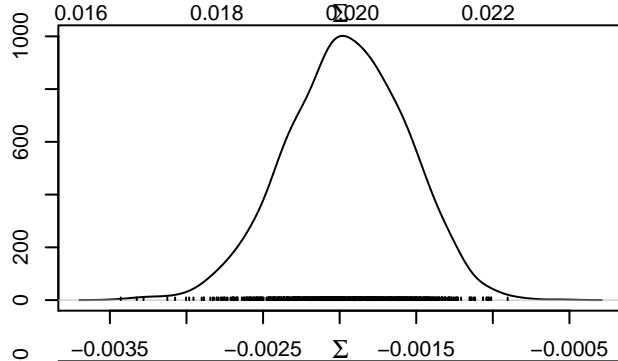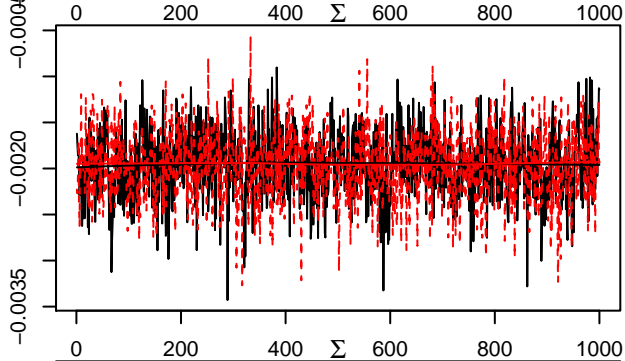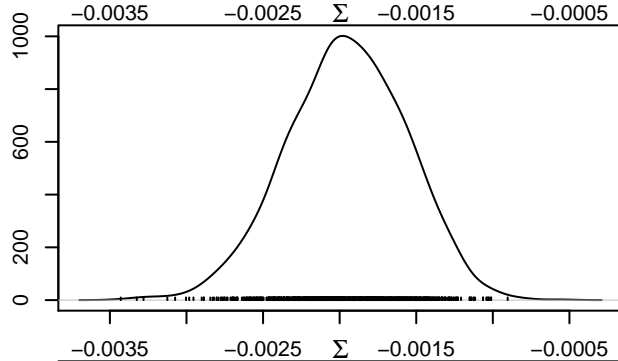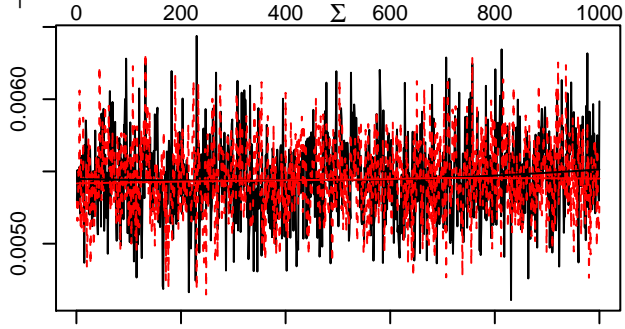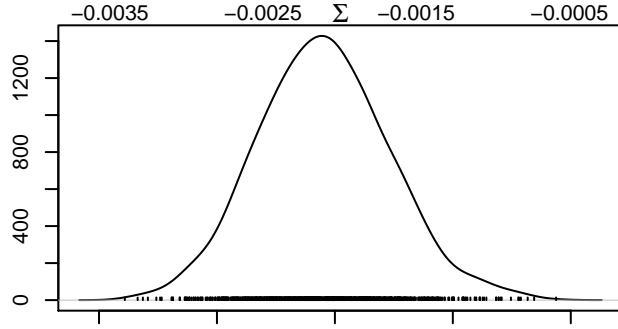

Supplement: Supplementary file 8 [file ECE3-6-8243-s008.pdf]

**FM07-S**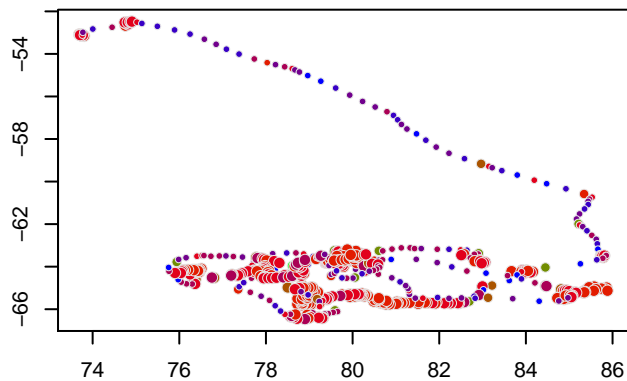**FM10-S**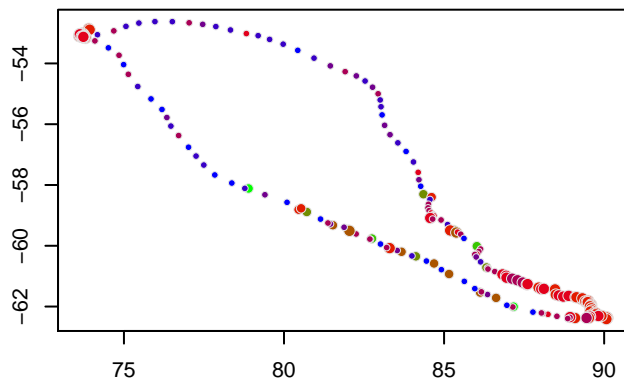**FM11-S**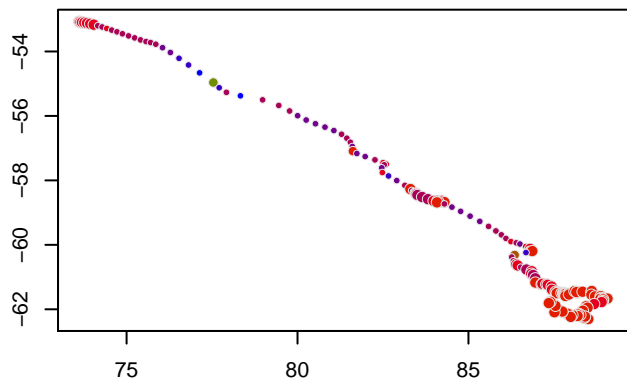**FM17-S**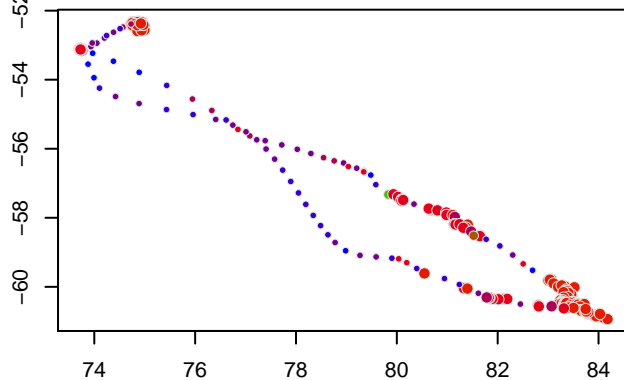**FM19-S**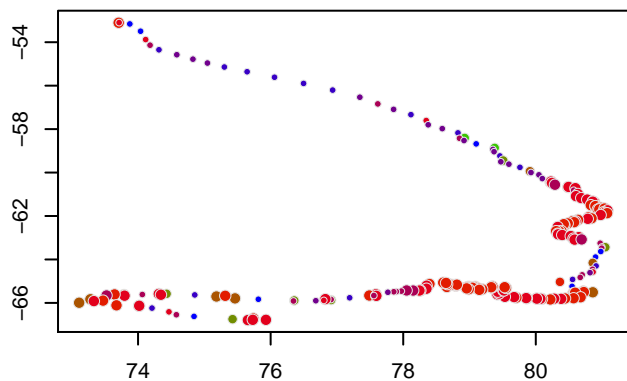

**FM07-S**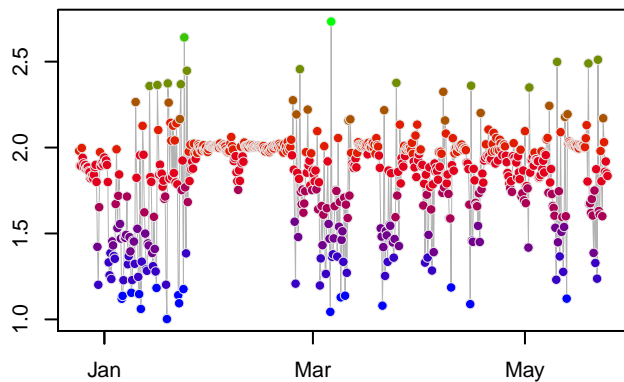**FM10-S**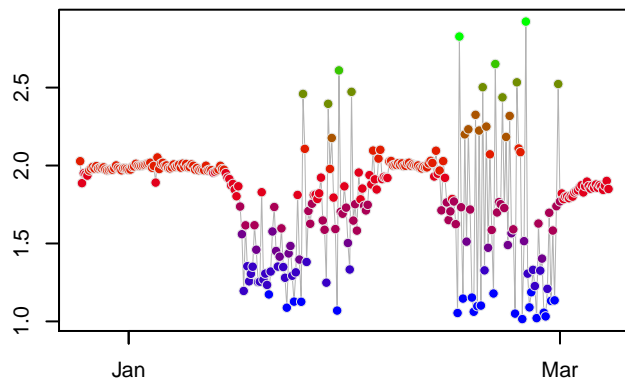**FM11-S**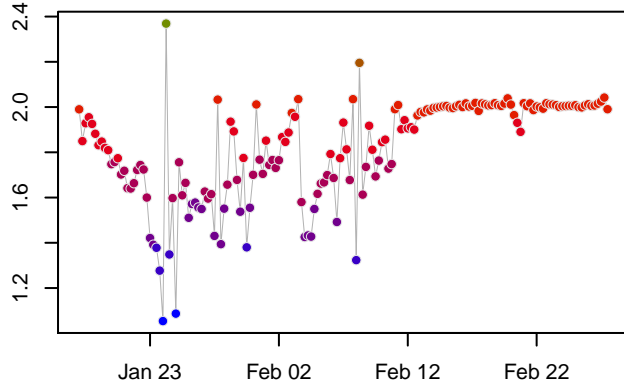**FM17-S**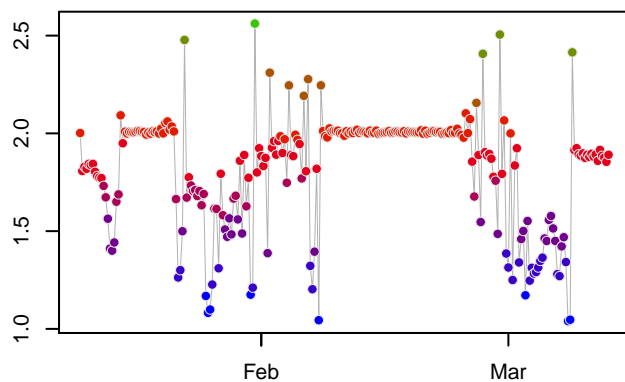**FM19-S**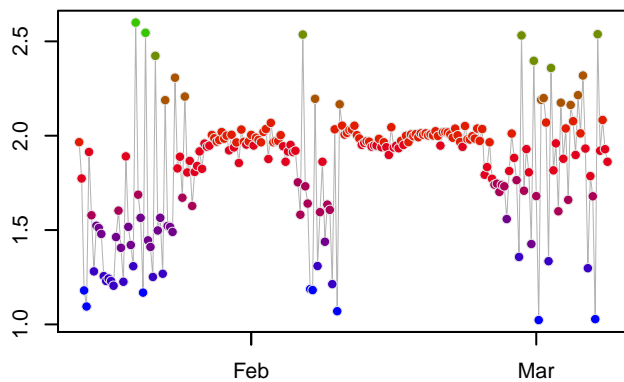

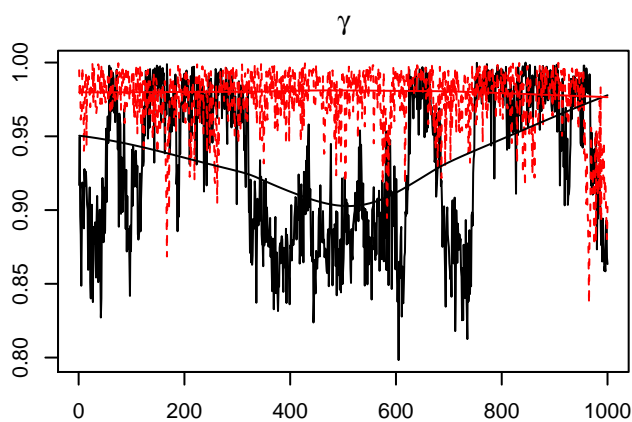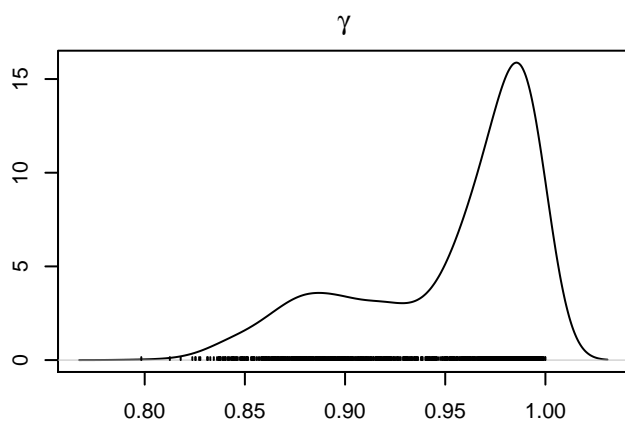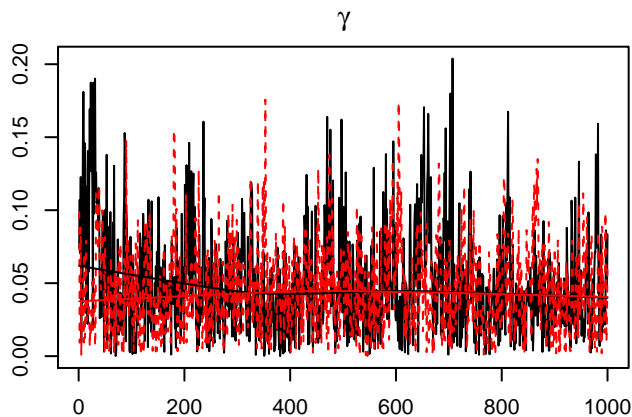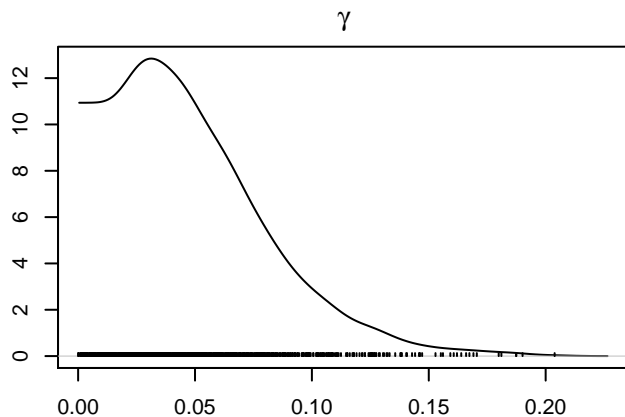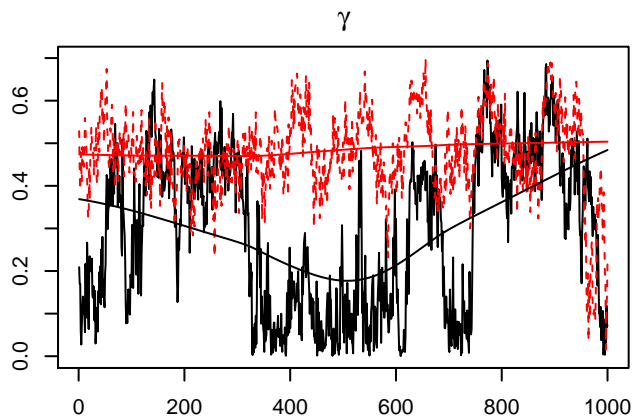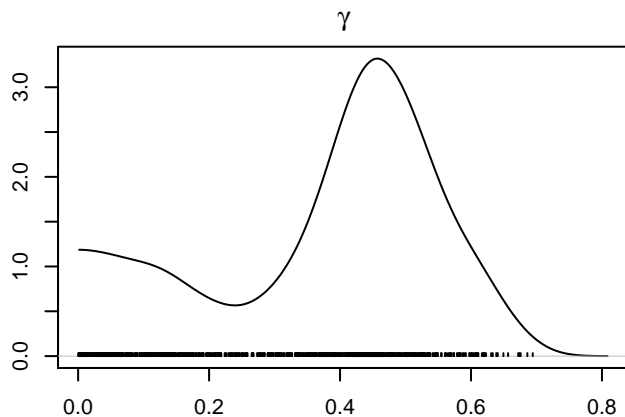

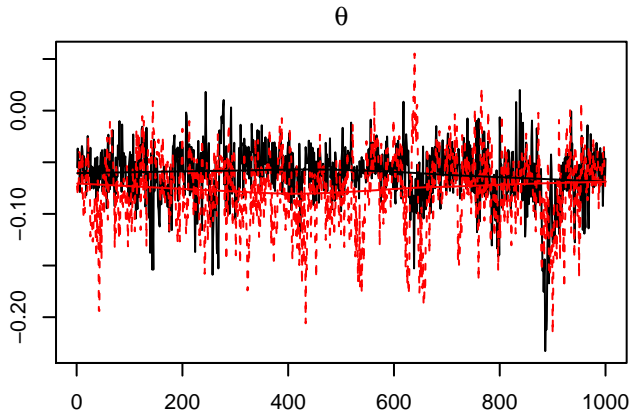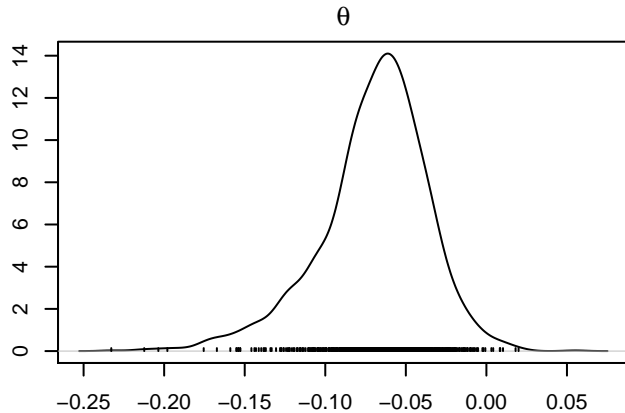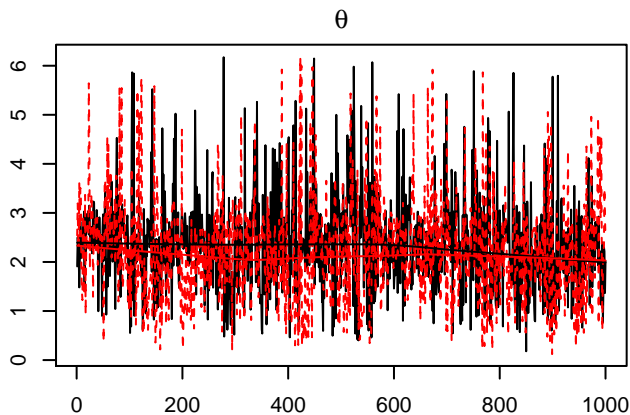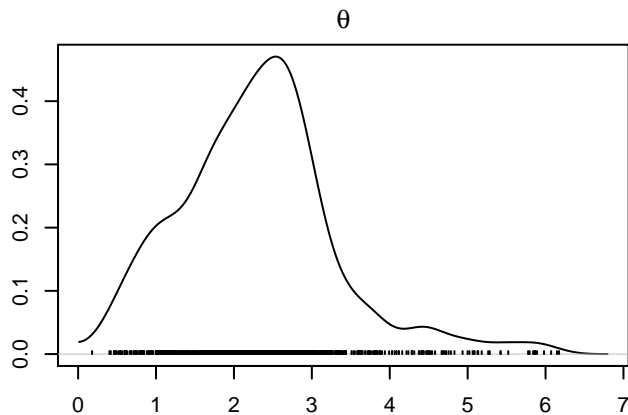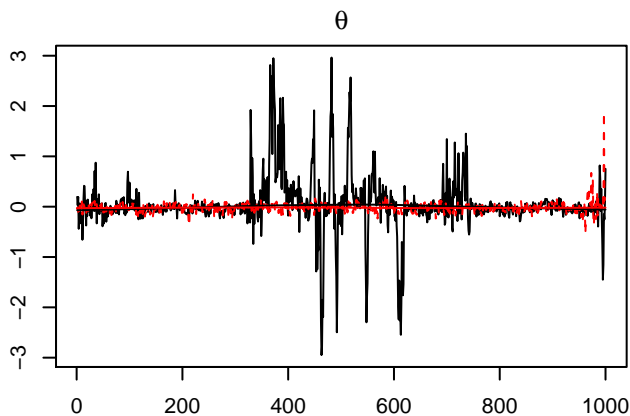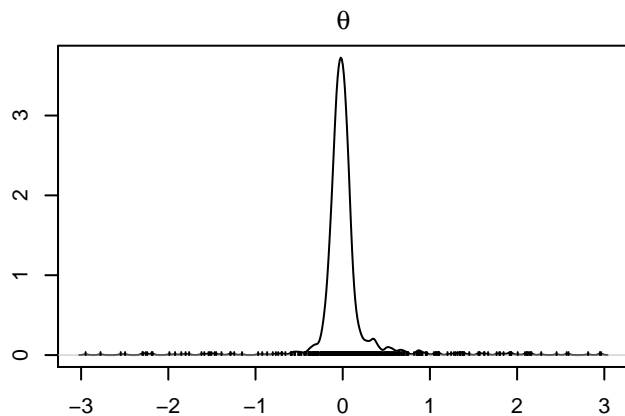

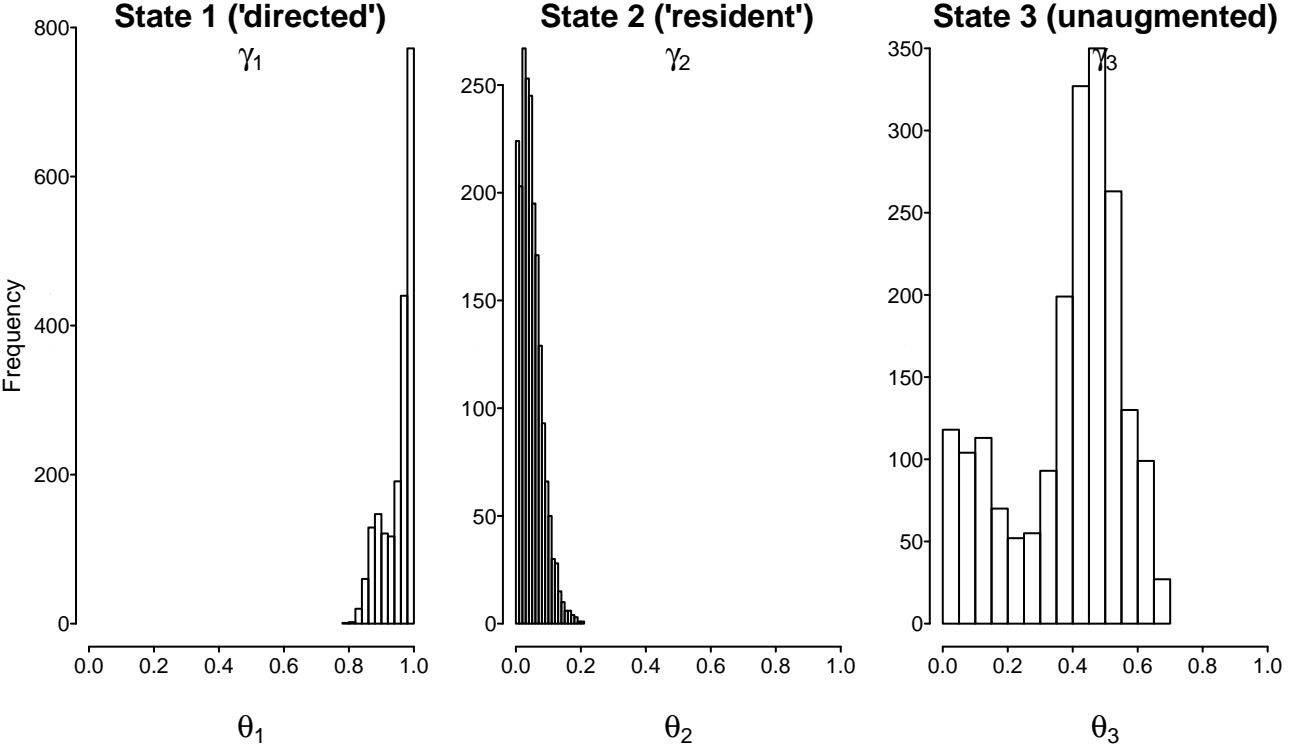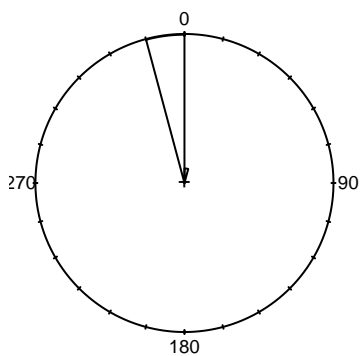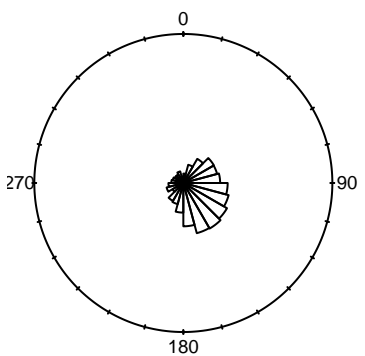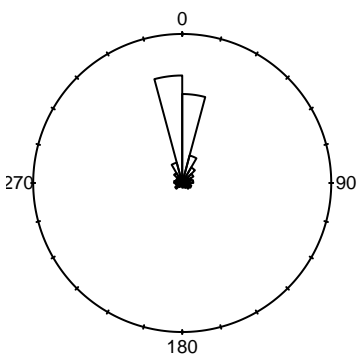

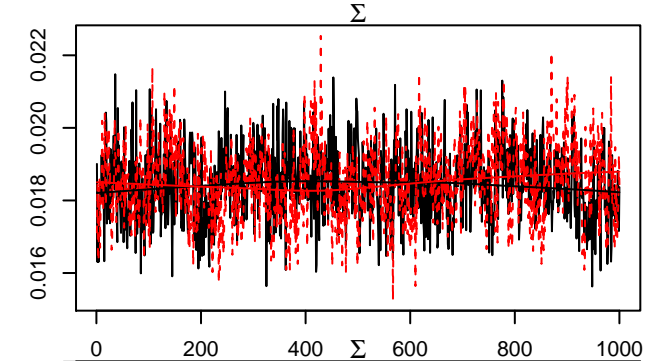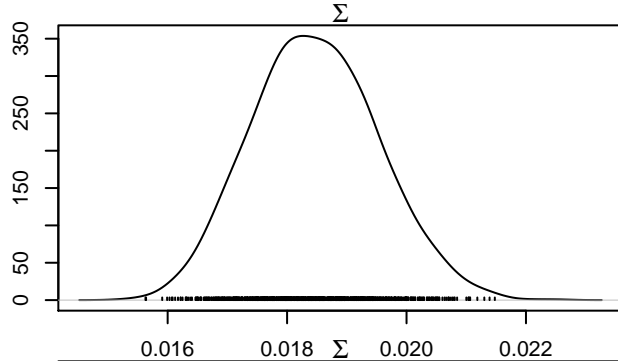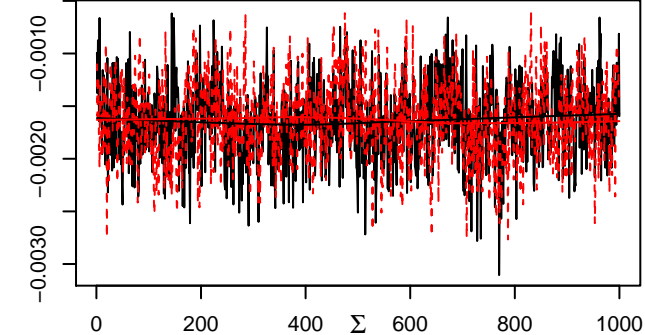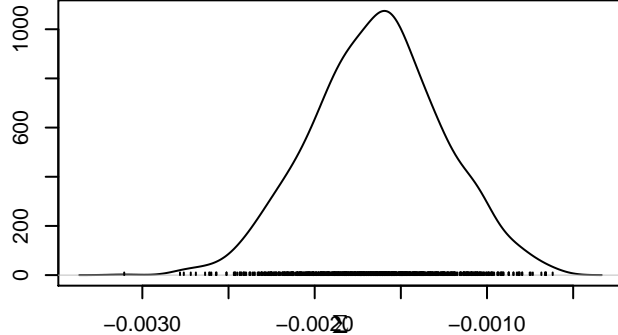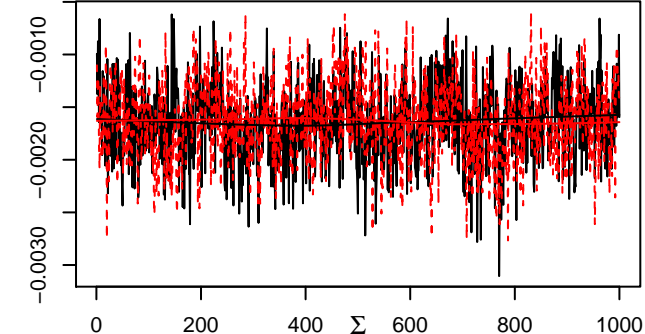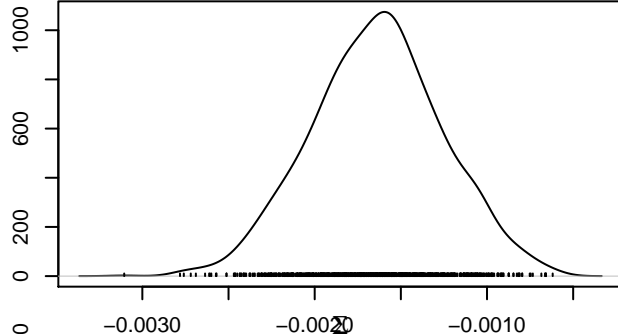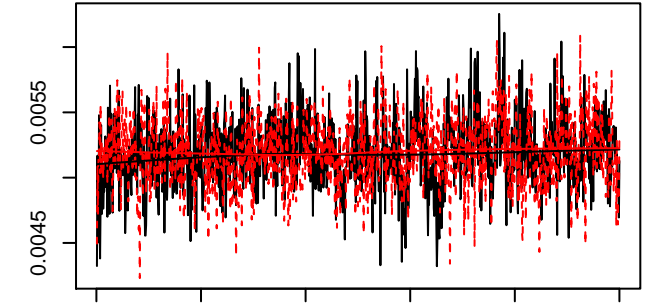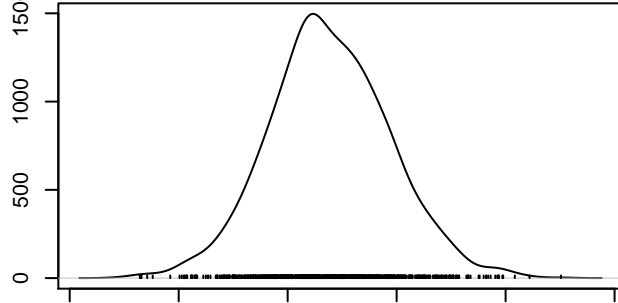

Supplement: Supplementary file 9 [file ECE3-6-8243-s009.pdf]

**FM07-S**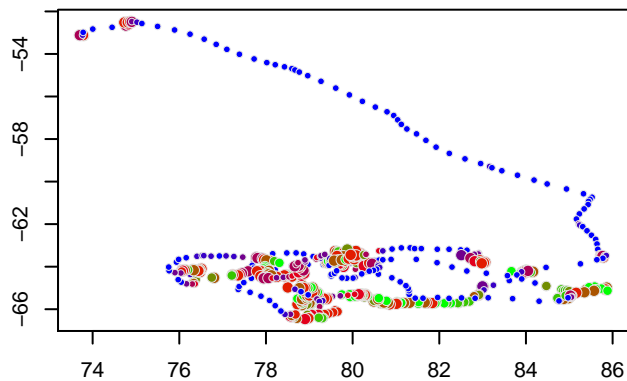**FM10-S**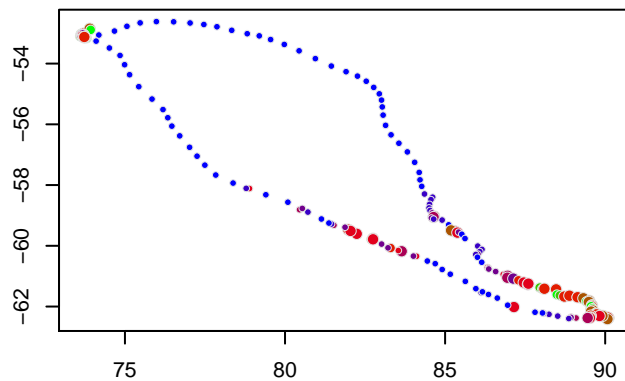**FM11-S**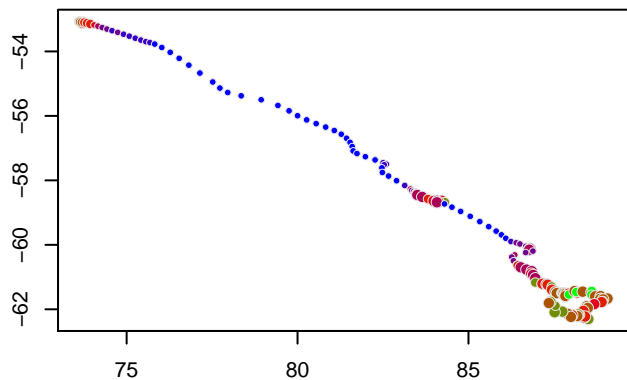**FM17-S**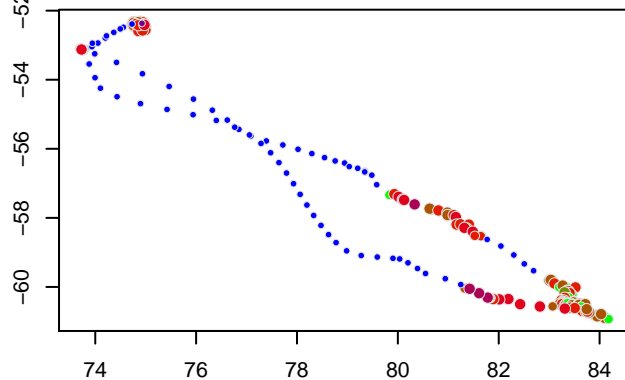**FM19-S**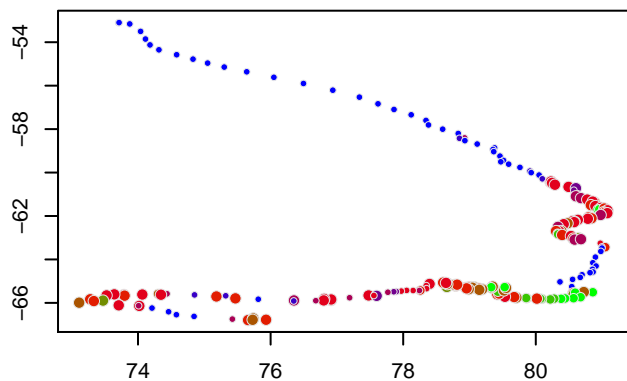

**FM07-S**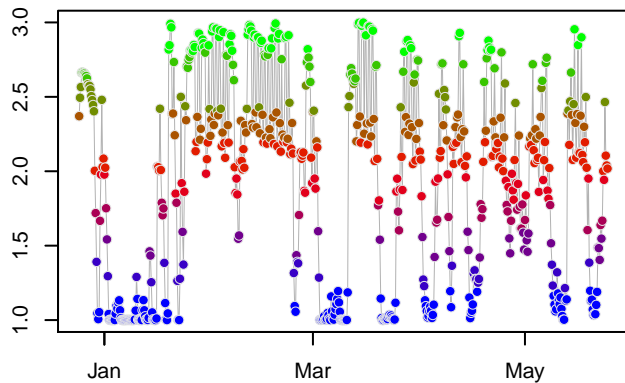**FM10-S**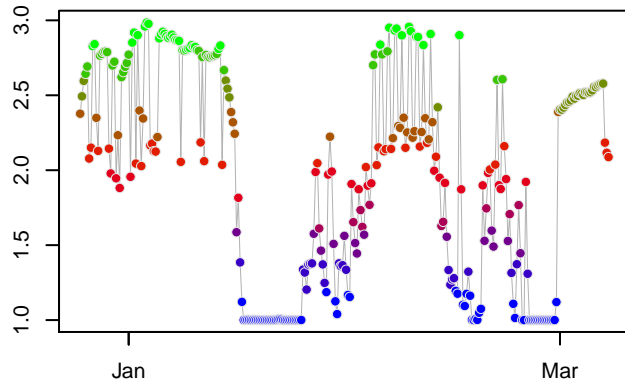**FM11-S**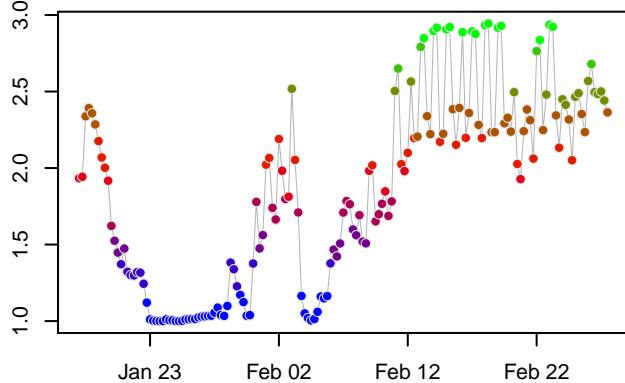**FM17-S**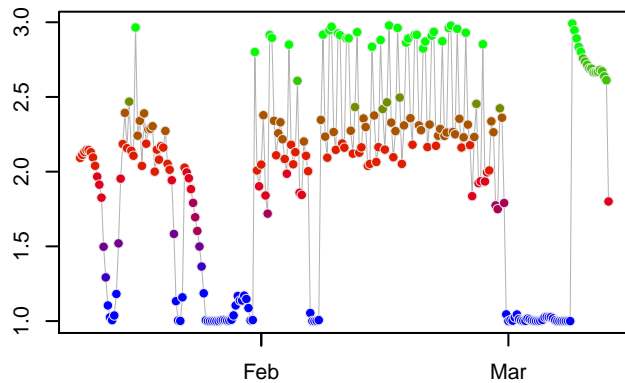**FM19-S**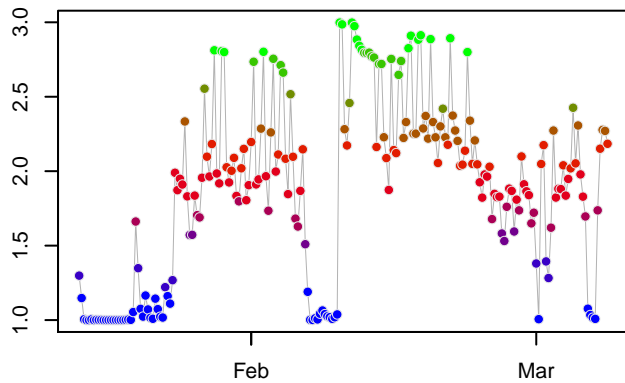

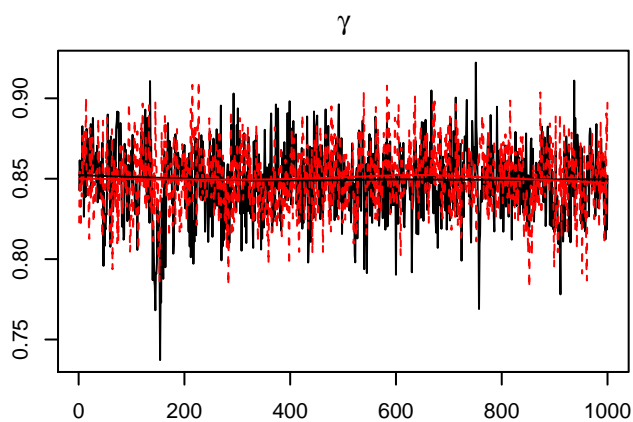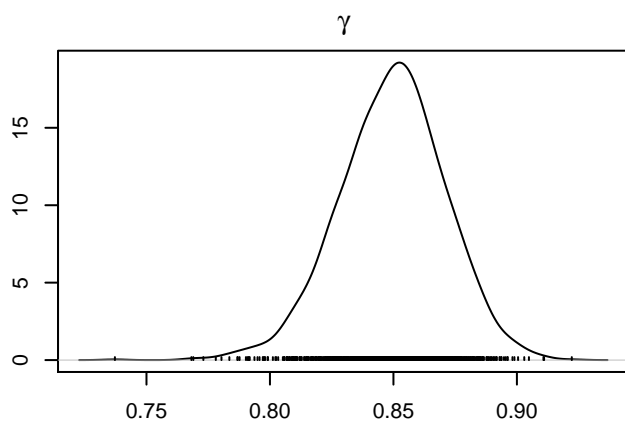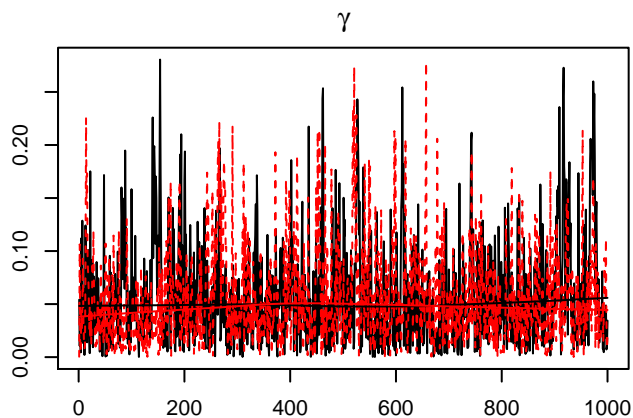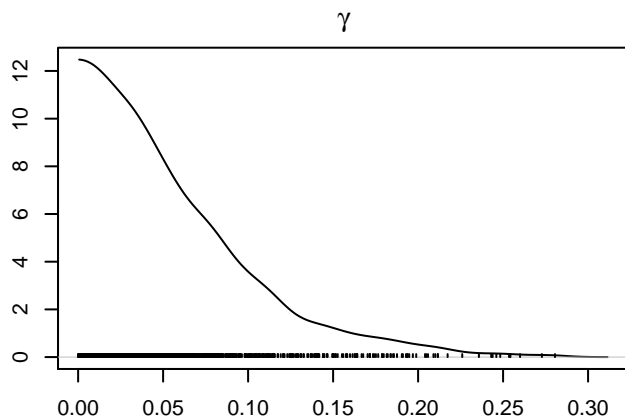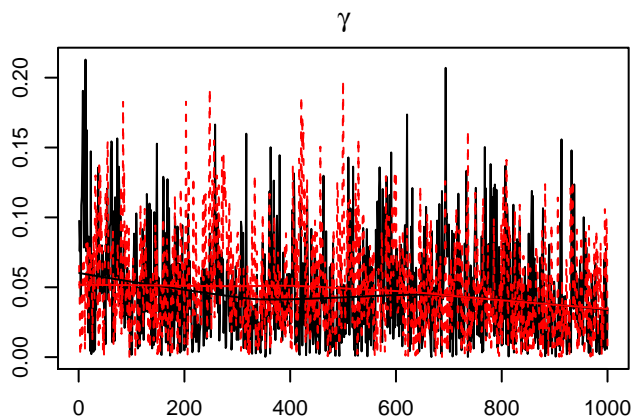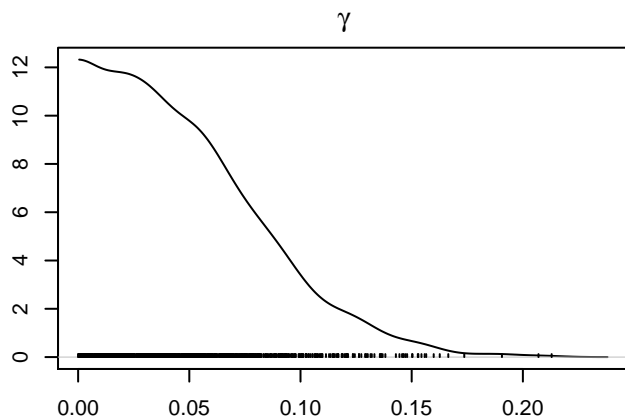

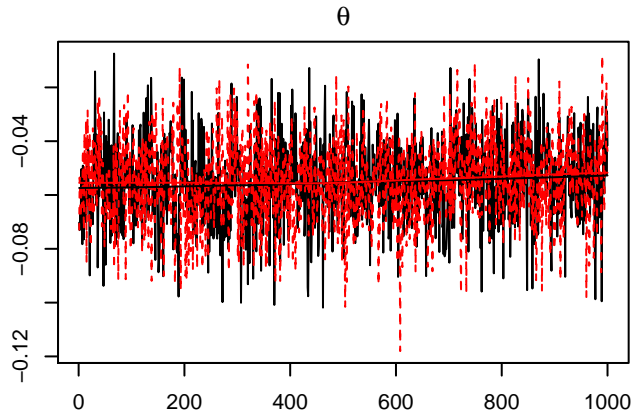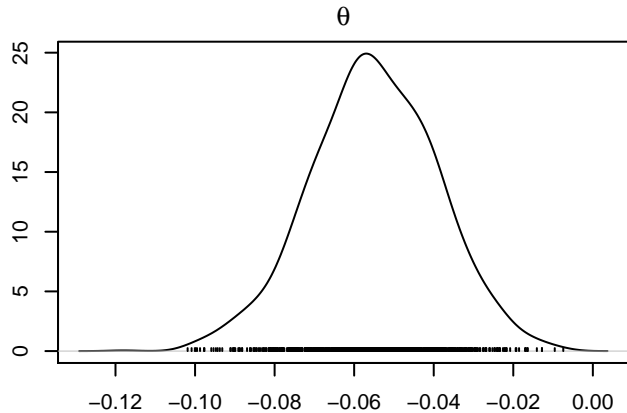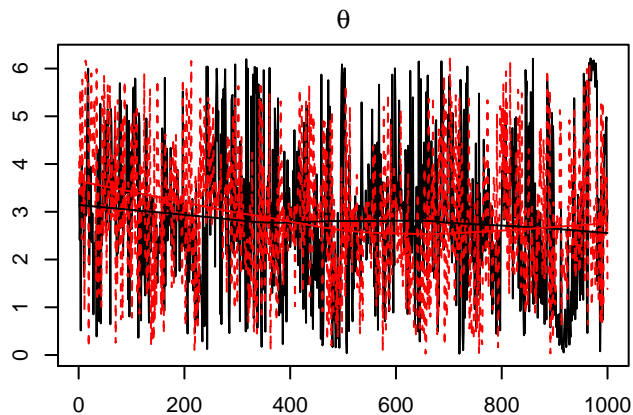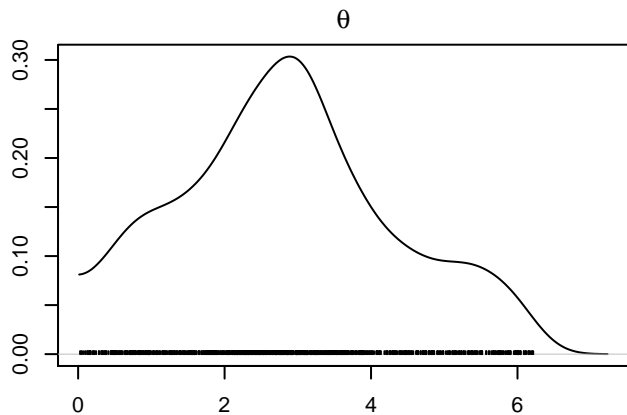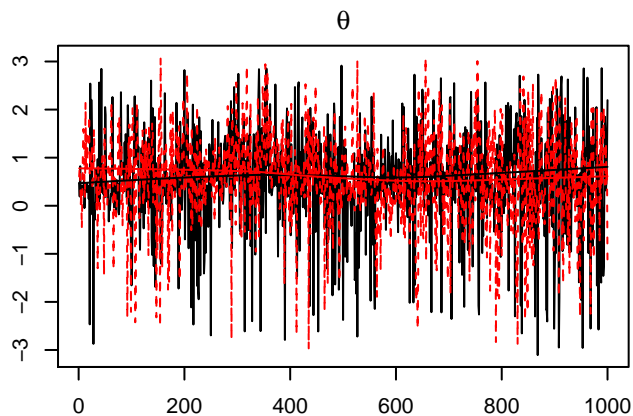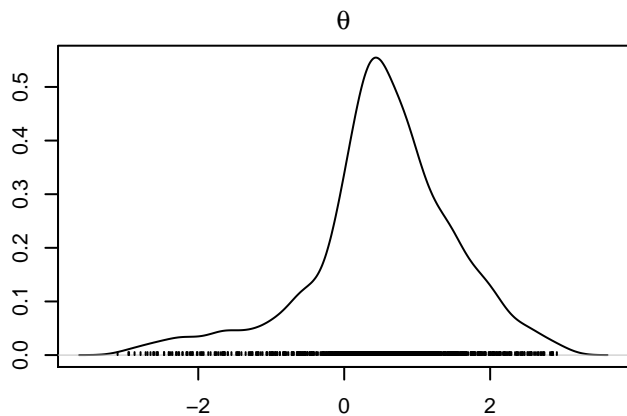

**State 1 ('directed')**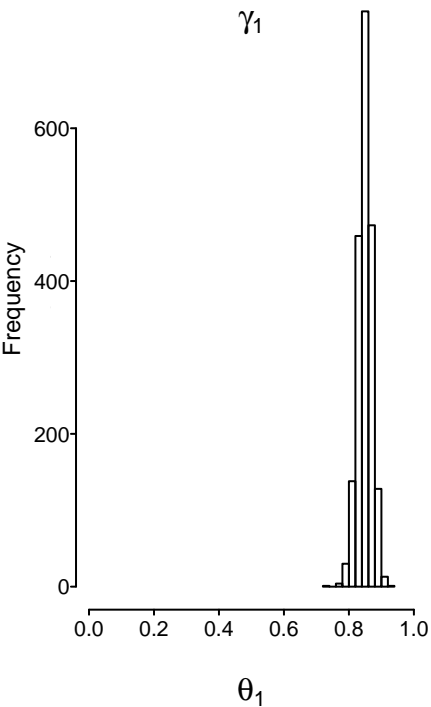**State 2 ('resident')**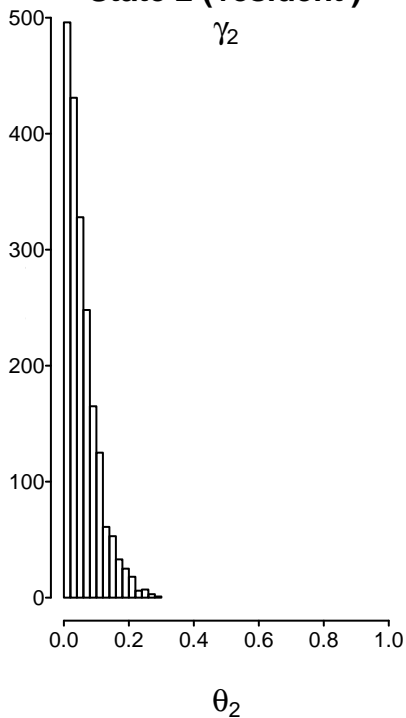**State 3 ('inactive')**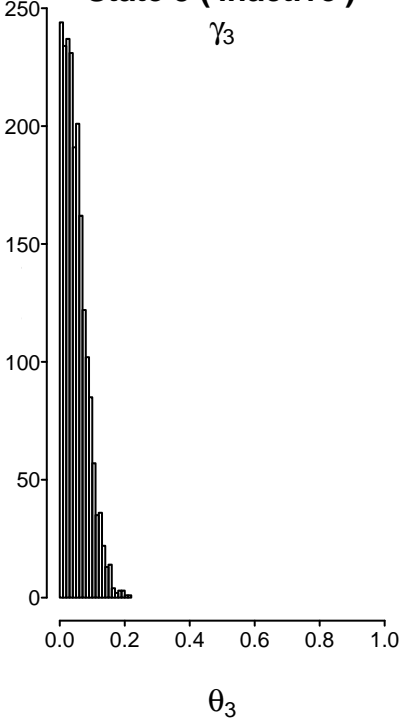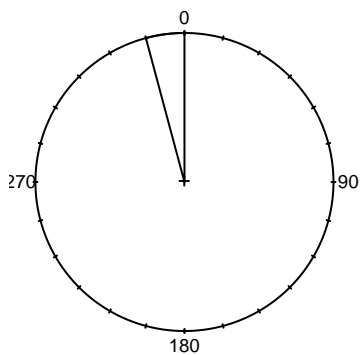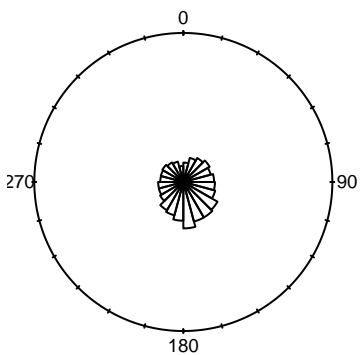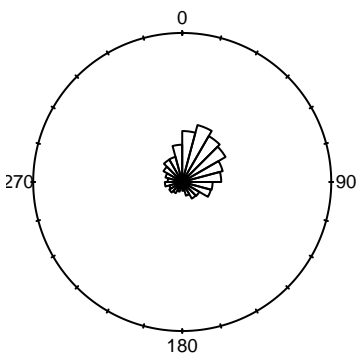

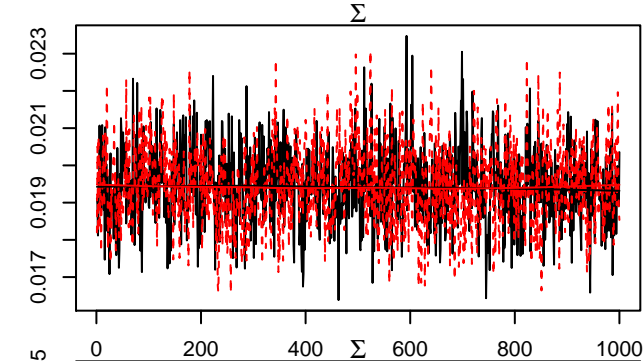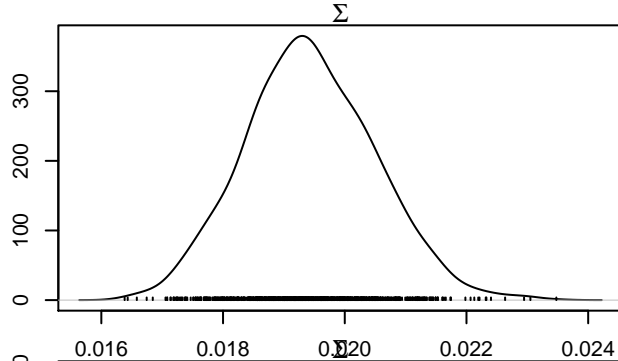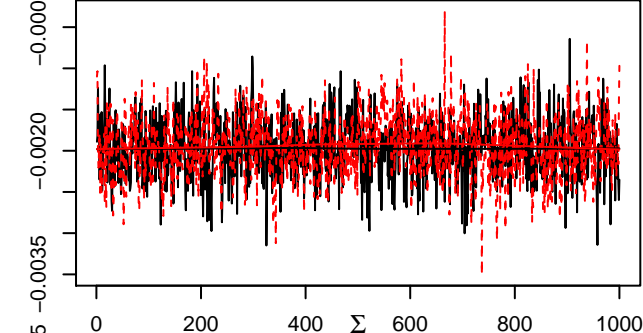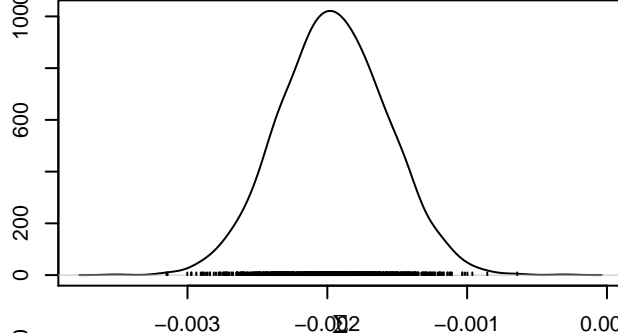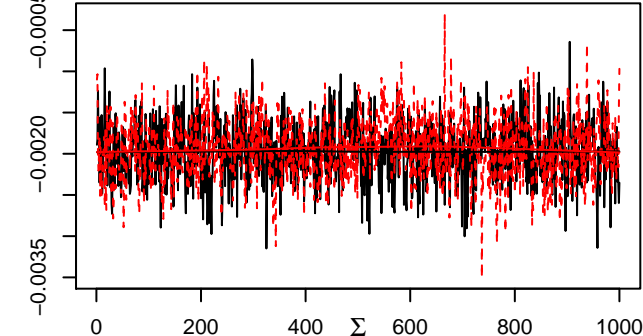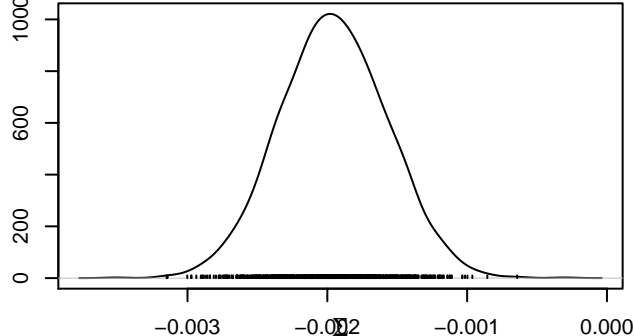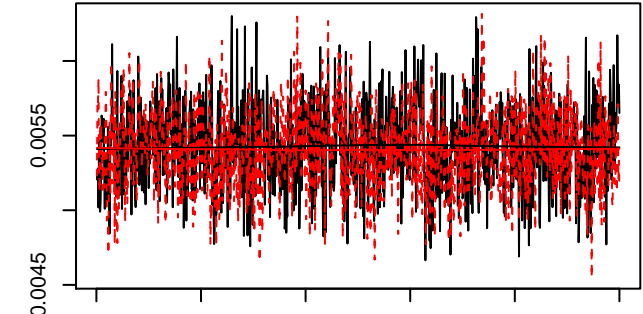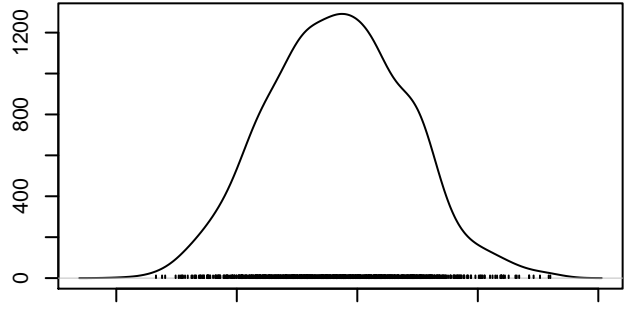

Supplement: Supplementary file 10 [file ECE3-6-8243-s010.pdf]
