# Supplementary material for: Putting the behavior into animal movement modeling: Improved activity budgets from use of ancillary tag information
Source: Ecol Evol. 2016 Oct 20;6(22):8243–55. doi: 10.1002/ece3.2530 (PMC5108274; doi:10.1002/ece3.2530)
Supplement: Supplementary file 11 [file ECE3-6-8243-s011.docx]

**Data S1: Source code and data for implementing the various state space model formulations.** Includes preparation of the location and ancillary tag data and complete worked case-study examples.

**File list**

example.r

dat4jags_reg.r

check.haulouts.tstep.r

check.divetime.tstep.r

hssm.r

hactssm.plt.r

WED_location.csv

WED_haulout.csv

AFS_location.csv

AFS_summary.csv

AFS_dive.csv

**Description**

Following the worked example given in the example.R file should enable readers to run each of the state-space model formulations presented in this paper, as applied to the two case study species; and/or fit their own Argos and ancillary tag data, using the R and JAGS codes provided. The JAGS code for each of the state-space model formulations are as from Appendix S1.

This supplement contains sample data, functions and scripts to prepare the data (dat4jags_reg.R, check.haulouts.tstep.R and check.dive.tstep.r), fit the state-space model formulations (hssm.R) and examine the output (hactssm.plt.r) as shown in Appendix S3.

Raw location data files contain Argos locations for Weddell and Antarctic fur seals (WED_location.csv, AFS_location.csv). Ancillary tag data files include haulout summaries (WED_haulout.csv), dive summaries (AFS_summary.csv) and individual dive records (AFS_dive.csv).

**example.r** contains R code for loading the data (.csv files) and running the case-study examples

**dat4jags_reg.r** is an R function which prepares ARGOS position data and writes input data for upload into JAGS

**check.haulouts.tstep.r** is an R function which processes the ancillary haulout summary data (Weddell case study)

**check.divetime.tstep.r** is an R function which processes the ancillary dive summary data (AFS case study)

**hssm.r** is an R function which implements the state-space models

**hactssm.plt.r** contains R functions for plotting the state-space model output and diagnostics

The Spp_location.csv file is a comma-separated file containing the raw Argos tracking data.

Column definitions

1. "id" – is a unique identifier for the animal from which the tracking dataset came.
2. "gmt" – is the GMT date-time of each observation with the following format: "2001-11-13 07:59:59".
3. "lc" – is the Argos location quality class of each observation. Values in ascending order of quality are: "Z", "B", "A", "0", "1", "2", "3".
4. "lon" – is the observed longitude in decimal degrees.
5. "lat" – is the observed latitude in decimal degrees.

The WED_haulout.csv file is a comma-separated file containing the Weddell seal summaries of haulout activity.

Column definitions

1. "ref" – is a unique identifier for the seal from which the dive dataset came (same as “id” for the location.csv file).
2. "S_DATE" – is the GMT date-time for the start of each recorded period of haulout activity with the following format: "2001-11-13 07:59:59".
3. "E_DATE" – is the GMT date-time for the end of each recorded period of haulout activity with the following format: "2001-11-13 07:59:59".

The AFS_summary.csv file is a comma-separated file containing the Antarctic fur seal 6h summaries of diving activity.

Column definitions

1. "ref" – is a unique identifier for the seal from which the dive dataset came (same as “id” for the location.csv file).
2. "gmt" – is the GMT date-time for the start of each 6h observation period with the following format: "2001-11-13 06:00:00".
3. "DIVE_TM" – the summary record for the 6h observation period giving the percent of time spent diving (0-100%).

The AFS_dive.csv file is a comma-separated file containing the Antarctic fur seal individual dive records (if available to supplement the 6h summary records).

Column definitions

1. "ref" – is a unique identifier for the seal from which the dive dataset came (same as “id” for the location.csv file).
2. "gmt" – is the GMT date-time of each observation with the following format: "2001-11-13 07:59:59".
3. "DIVE_DUR" – is the duration of the dive in seconds. Any dive variable (with any name) can occur here, since strictly only the timestamp is used to indicate some diving activity.
